# A large-scale binding and functional map of human RNA-binding proteins

**DOI:** 10.1038/s41586-020-2077-3

**Published:** 2020-07-29

**Authors:** Eric L. Van Nostrand, Peter Freese, Gabriel A. Pratt, Xiaofeng Wang, Xintao Wei, Rui Xiao, Steven M. Blue, Jia-Yu Chen, Neal A. L. Cody, Daniel Dominguez, Sara Olson, Balaji Sundararaman, Lijun Zhan, Cassandra Bazile, Louis Philip Benoit Bouvrette, Julie Bergalet, Michael O. Duff, Keri E. Garcia, Chelsea Gelboin-Burkhart, Myles Hochman, Nicole J. Lambert, Hairi Li, Michael P. McGurk, Thai B. Nguyen, Tsultrim Palden, Ines Rabano, Shashank Sathe, Rebecca Stanton, Amanda Su, Ruth Wang, Brian A. Yee, Bing Zhou, Ashley L. Louie, Stefan Aigner, Xiang-Dong Fu, Eric Lécuyer, Christopher B. Burge, Brenton R. Graveley, Gene W. Yeo

**Affiliations:** 1 Department of Cellular and Molecular Medicine, University of California San Diego, La Jolla, CA USA; 2 Institute for Genomic Medicine, University of California San Diego, La Jolla, CA USA; 3 Program in Computational and Systems Biology, Massachusetts Institute of Technology, Cambridge, MA USA; 4 Bioinformatics and Systems Biology Graduate Program, University of California San Diego, La Jolla, CA USA; 5 Institut de Recherches Cliniques de Montréal (IRCM), Montreal, Quebec Canada; 6 Department of Genetics and Genome Sciences, Institute for Systems Genomics, UConn Health, Farmington, CT USA; 7 Medical Research Institute, Wuhan University, Wuhan, China; 8 Department of Biology, Massachusetts Institute of Technology, Cambridge, MA USA; 9 Department of Biochemistry and Molecular Medicine, Université de Montréal, Montreal, Quebec Canada; 10 Department of Biological Engineering, Massachusetts Institute of Technology, Cambridge, MA USA; 11 Division of Experimental Medicine, McGill University, Montreal, Quebec Canada

**Keywords:** Transcriptomics, Alternative splicing

## Abstract

Many proteins regulate the expression of genes by binding to specific regions encoded in the genome^1^. Here we introduce a new data set of RNA elements in the human genome that are recognized by RNA-binding proteins (RBPs), generated as part of the Encyclopedia of DNA Elements (ENCODE) project phase III. This class of regulatory elements functions only when transcribed into RNA, as they serve as the binding sites for RBPs that control post-transcriptional processes such as splicing, cleavage and polyadenylation, and the editing, localization, stability and translation of mRNAs. We describe the mapping and characterization of RNA elements recognized by a large collection of human RBPs in K562 and HepG2 cells. Integrative analyses using five assays identify RBP binding sites on RNA and chromatin in vivo, the in vitro binding preferences of RBPs, the function of RBP binding sites and the subcellular localization of RBPs, producing 1,223 replicated data sets for 356 RBPs. We describe the spectrum of RBP binding throughout the transcriptome and the connections between these interactions and various aspects of RNA biology, including RNA stability, splicing regulation and RNA localization. These data expand the catalogue of functional elements encoded in the human genome by the addition of a large set of elements that function at the RNA level by interacting with RBPs.

## Abstract (web summary)

A combination of five assays is used to produce a catalogue of RNA elements to which RNA-binding proteins bind in human cells.

## Main

RBPs are a diverse class of proteins that are involved in regulating gene expression^1^. They interact with RNA to form ribonucleoprotein complexes, which govern the maturation and fate of their target RNA substrates and regulate numerous aspects of gene expression, including pre-mRNA splicing, cleavage and polyadenylation, RNA stability, RNA localization, RNA editing, and translation. Many RBPs participate in more than one of these processes, such as regulation of both alternative splicing and poly(A) site usage by NOVA^2^. These roles are essential for normal human physiology, as defects in RBP function are associated with genetic and somatic disorders, such as neurodegeneration, autoimmunity and cancer^3^. The regulatory roles of RBPs are also affected by the subcellular localization of RBPs and their RNA substrates, as post-transcriptional steps are often carried out in both membrane- and phase-separated sub-cellular compartments.

Traditionally, RBPs were identified by the affinity purification of single proteins^4^. However, recent mass spectrometry-based methods have identified hundreds of proteins bound to RNA in human and mouse cells^5–8^, suggesting that the human genome may contain 1,542 or more RBP-encoding genes^1^. This large repertoire of RBPs is likely to underlie the tremendous complexity of post-transcriptional regulation, motivating efforts to systematically investigate the binding properties, RNA targets, and functional roles of these proteins.

The elucidation of RBP–RNA regulatory networks requires the integration of multiple data types, each viewing the RBP through a different lens. In vivo binding assays such as crosslinking and immunoprecipitation (IP) followed by sequencing (CLIP-seq) provide a set of candidate functional elements that are directly bound by each RBP. Assessments of in vitro binding affinity uncover the mechanisms that drive these interactions and improve the identification of functional associations. Functional assays that identify targets whose expression or alternative splicing responds to RBP perturbation can strengthen evidence of function. For example, the observation by CLIP-seq of protein binding within introns flanking exons whose splicing is sensitive to RBP levels provides support for the RBP as a splicing factor and for the binding sites as splicing regulatory elements. In vivo interactions of RBPs with chromatin can also be assayed to provide insight into the roles of some RBPs as transcriptional regulators and to provide evidence for co-transcriptional deposition of RBPs on target RNA substrates. Thus, integration of these data types can identify both factor-specific regulatory modules and the roles of RBPs in broader cellular regulatory networks.

• We report, to our knowledge, the largest effort to date to systematically map and study the functions of 356 human RBPs using integrative approaches consisting of 5 assays that focus on different aspects of RBP activity.
• We examine in vivo binding activity using enhanced CLIP (eCLIP) assays. Two hundred and twenty-three eCLIP data sets for 150 RBPs provide a set of candidate functional elements directly bound by each RBP and show a variety of in vivo RNA target classes.
• We infer the functions of the RNA elements identified by eCLIP through analyses of 472 knockdown followed by RNA sequencing (KD–RNA-seq) profiles of 263 RBPs, identifying RNA expression and splicing regulatory patterns.
• We decipher the in vitro binding specificities of 78 RBPs using RNA Bind-N-Seq assays and identify connections between in vitro and in vivo binding. We find that eCLIP peaks containing in vitro motifs are more strongly associated with regulation.
• We map the subcellular localization of 274 RBPs using immunofluorescence, indicating widespread organelle-specific regulation of RNA processing.
• We profile the DNA association patterns of 39 RBPs by chromatin IP and sequencing (ChIP–seq), suggesting that there is broad interconnectivity between chromatin association and RNA processing.

## Overview of data and processing

**Fig. 1:**
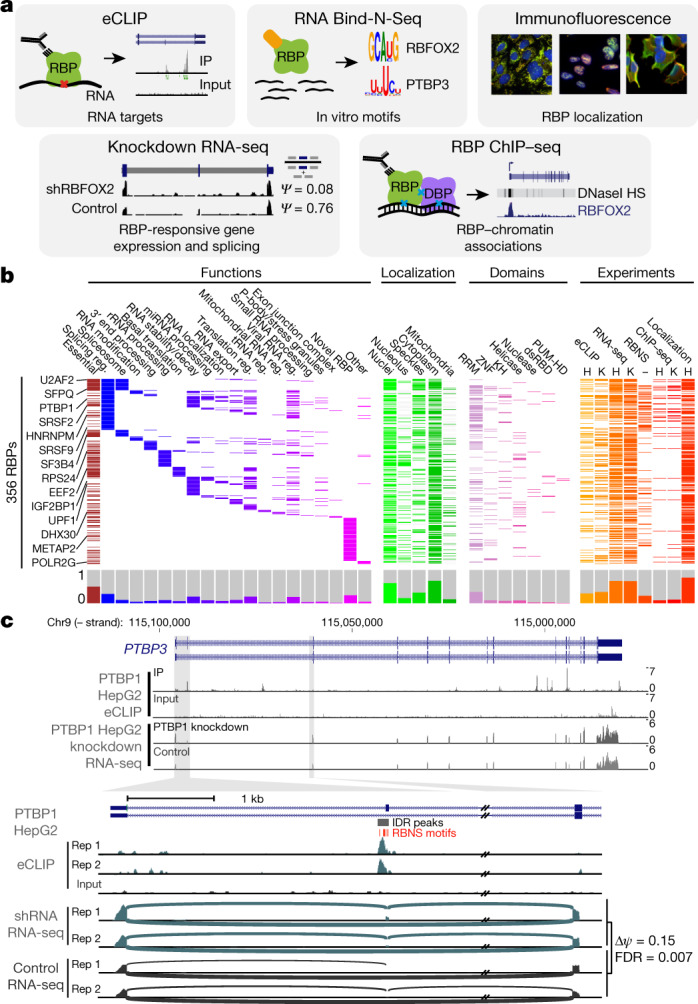
Overview of experiments and data types. **a**, The five assays performed to characterize RBPs. **b**, Three hundred and fifty-six RBPs profiled by at least one ENCODE experiment (orange or red) with localization by immunofluorescence (green), essential genes from CRISPR screening (maroon), manually annotated RBP functions (blue or purple), and annotated protein domains (pink; RRM, KH, zinc finger, RNA helicase, RNase, double-stranded RNA binding (dsRBD), and pumilio/FBF domain (PUM-HD)). Histograms for each category are shown at bottom. **c**, Combinatorial expression and splicing regulation of *PTBP3*. Tracks indicate eCLIP and RNA-seq read density (reads per million). Tracks are shown for replicate 1; eCLIP and KD–RNA-seq were performed in biological duplicate with similar results. Bottom, alternatively spliced exon 2, with lines indicating junction-spanning reads and indicated per cent spliced in (*ψ*). Boxes indicate reproducible (by IDR) PTBP1 peaks, with red boxes indicating RBNS motifs for the PTB family member PTBP3 located within (or up to 50 bases upstream of) peaks.

To work towards developing a comprehensive understanding of the binding and function of the human RBP repertoire, we used five assays to produce 1,223 replicated data sets for 356 RBPs that participate in diverse aspects of RNA biology and encompass diverse sequence and structural characteristics (Fig. 1a, b, Supplementary Data 1, 2). Functionally, these RBPs most commonly contribute to the regulation of RNA splicing (98 RBPs, 28%), and more than one function has been reported for 162 RBPs (46%), but 83 (23%) have no verified mechanistic RNA function in humans (Fig. 1b, Supplementary Data 1). Although 57% of the RBPs surveyed contain well-characterized RNA-binding domains, the remainder possess less well-studied domains or lack known RNA-binding domains altogether (Fig. 1b, Supplementary Data 1). Many RBPs, including the ribosomal protein RPL23A and splicing factor HNRNPC, are highly expressed in ENCODE cell lines and across a broad range of human tissues, but some have highly tissue-specific expression, indicating that the regulatory activity of these RBPs is likely to be modulated through cell type-specific gene expression programs (Supplementary Fig. 1, Supplementary Data 3).

Each of the five assays used focused on a distinct aspect of RBP activity (Fig. 1a), as described below.

## Transcriptome-wide RNA-binding sites of RBPs

We identified and validated hundreds of IP-grade antibodies that recognize human RBPs^9^ and developed enhanced CLIP (eCLIP)^10^. We generated high-quality eCLIP profiles for 120 RBPs in K562 cells and 103 in HepG2 cells (73 in both cell types, a total of 150 profiled RBPs; Supplementary Data 4). This effort identified 844,854 significantly enriched peaks that cover 18.5% of the annotated mRNA transcriptome and 2.6% of the pre-mRNA transcriptome.

## RBP-responsive genes and alternative splicing events

To obtain insight into the functions of eCLIP peaks, we used short hairpin RNA (shRNA) or CRISPR to deplete individual RBPs followed by RNA sequencing (RNA-seq). We depleted 235 RBPs in K562 cells and 237 RBPs in HepG2 cells (209 in both cell types, a total of 263 RBPs; Supplementary Data 5). Comparison against paired non-target control data sets identified 375,873 instances of differentially expressed genes with 20,542 genes affected upon knockdown of at least one RBP, as well as 221,612 cases of differential splicing involving 38,555 alternatively spliced events that were affected upon knockdown of at least one RBP (Supplementary Text, Supplementary Figs. 2–6). Further analysis indicated GC content-dependent effects on read density in some datasets, which was resolved by normalization with Salmon and CQN software tools (Supplementary Text, Supplementary Fig. 2). In addition to within-batch controls for each experiment, batch correction enabled integrated analyses across the entire data set (Supplementary Text, Supplementary Fig. 7).

## In vitro RBP binding motifs

We used RNA Bind-N-Seq (RBNS)^11^ with recombinant purified RBPs and pools of random RNA oligonucleotides to identify the RNA sequences and structural binding preferences of 78 RBPs in vitro^12^ (Supplementary Data 6). For about half of the RBPs assayed (37 of 78), we were able to identify highly enriched *k*mers of five nucleotides (nt) (*k* = 5) that could be clustered into a single motif. The remaining RBPs had more complex patterns of binding, best described by two motifs (32 of 78), or even three or more motifs (9 RBPs). These data also indicate that many RBPs are sensitive to the sequence and RNA structural context in which motifs are embedded.

## Subcellular localization of RBPs

To illuminate the functional properties of RBPs in intracellular space, we used our validated antibodies^9^ to conduct systematic immunofluorescence imaging of 274 RBPs in HepG2 cells and 268 RBPs in HeLa cells, in conjunction with 12 markers for specific organelles and subcellular structures (Supplementary Data 1). These data, encompassing 217,412 images and controlled vocabulary localization descriptors, have been organized within the RBP Image Database (http://rnabiology.ircm.qc.ca/RBPImage/).

## Association of RBPs with chromatin

To study the role that the association of RBP with chromatin has in transcription and co-transcriptional splicing^13,14^, we performed ChIP–seq to generate a resource of DNA elements associated with 37 RBPs (30 RBPs in HepG2 cells and 33 RBPs in K562 cells, with 26 in both cell types; Supplementary Data 7). These experiments identified 792,007 ChIP–seq peaks, covering 3.8% of the genome.

## Integrated data analysis

To facilitate integrated analyses, all data for each data type were processed by the same data processing pipeline, and consistent, stringent quality-control metrics and data standards were uniformly applied to all experiments. We studied 249 of the 356 RBPs (70%) using at least two different assays and 129 (37%) using at least three different assays, providing opportunities for integrated analysis using multiple data sets as shown for regulation of *PTBP3* by PTBP1 (Fig. 1c). The inclusion of exon 2 of *PTBP3* in mRNA alters start codon usage and increases cytoplasmic localization of PTBP3 protein, and *PTBP3* exon 2 was absent in control cells but increased upon PTBP1 knockdown, consistent with previous studies^15^. This splicing event is likely to be directly regulated by PTBP1, as we observed eCLIP peaks at the 3′ splice site of *PTBP3* exon 2 that contain U-rich motifs bound by PTB family proteins in RBNS. We also observed strong binding to *PTBP3* exon 10, which does not show alternative splicing itself but is orthologous to *PTBP1* exon 10 and *PTBP2* exon 11, which are alternatively spliced in a PTBP1-and PTBP2-regulated manner that triggers nonsense-mediated mRNA decay^16^. Thus, it appears that the absence of regulation of *PTBP3* exon 10 splicing by PTBP1 is not due to the loss of PTBP1 binding in this paralogue. As another example, we observed eCLIP enrichment for HNRNPL downstream of a cryptic exon of *GTPBP2* that contains repeats of the top HNRNPL RBNS motif, suggesting that HNRNPL represses splicing of the exon and contributes to the production of *GTPBP2* mRNA with a full-length open reading frame (Supplementary Fig. 8).

## Assessment and analysis of eCLIP data sets

**Extended Data Fig. 1:**
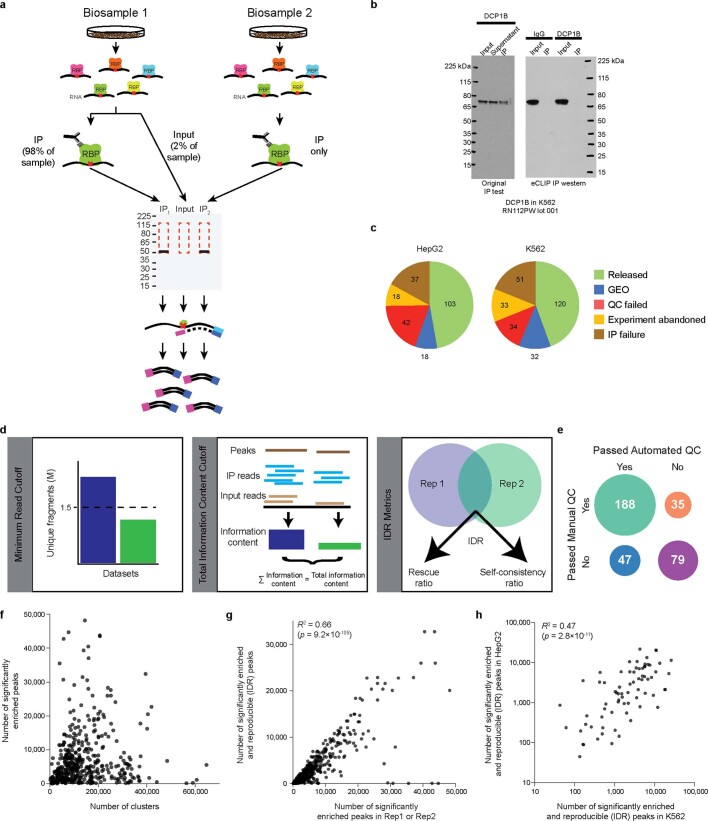
Experimental quality assessment of eCLIP assays. **a**, Model of ENCODE eCLIP experiments. Inputs were taken by sampling 2% of one of the two biosamples before IP. **b**, Example IP–western image for DCP1B IP success in K562 cells during initial IP tests performed without enzymatic steps (left) and IP failure in K562 cells during eCLIP experiments (right). This experiment was performed once. **c**, Pie charts indicate the number of eCLIP experiments that fell into the following categories: failure to successfully immunoprecipitate during eCLIP (IP failure), failure to yield amplifiable library in fewer than 20 PCR cycles (experiment abandoned), experiments that yielded immunoprecipitated library and were sequenced but failed quality assessment (QC failed), successful experiments that did not meet ENCODE standards but contained reproducible signal and have been released on the GEO, and successful experiments that met ENCODE standards and are available at the ENCODE Data Coordination Center (released). **d**, Schematic of eCLIP data quality standards. See Supplementary Text and Supplementary Fig. 11 for additional details. **e**, Confusion matrix of final classification scheme versus manual quality assessment. **f**, The number of CLIP per-identified clusters (*x*-axis) versus the number of significantly enriched peaks (*y*-axis) (fold enrichment ≥ 8 and *P* ≤ 0.001 from two-sided Fisher’s exact Test (or Yates’s *χ*^2^ test where appropriate) with no hypothesis testing correction (Methods)) identified for each of 446 eCLIP experimental replicates. **g**, The number of significantly enriched peaks (fold enrichment ≥ 8 and *P* ≤ 0.001 from two-sided Fisher’s exact rest (or Yates’ *χ*^2^ test where appropriate) with no hypothesis testing correction (Methods)) identified in each of replicate 1 and replicate 2 versus the number of reproducible peaks identified from IDR analysis for 223 eCLIP experiments. Pearson correlation and significance were determined in MATLAB. **h**, The number of significant and reproducible peaks identified in K562 cells (*x*-axis) versus HepG2 cells (*y*-axis) as in **g**, for all 73 RBPs with eCLIP in both cell types. Pearson correlation and significance were determined in MATLAB.

We performed 488 eCLIP experiments, each including biological duplicate IPs and a paired size-matched input (Extended Data Fig. 1, Supplementary Data 4, 8–10, Supplementary Figs. 9,10). Manual quality assessment was based on IP validation, library yield, presence of reproducible peak or repeat family signal, motif enrichment (for RBPs with known binding motifs) and consistency with established biological functions, and yielded the 223 high-quality eCLIP data sets described here and released at the ENCODE Data Coordination Center (https://www.encodeproject.org). An additional 50 data sets were not included in further analyses as they did not meet these stringent standards but contained a reproducible signal (Gene Expression Omnibus (GSE107768); Extended Data Fig. 1c, Supplementary Data 9). Automated metrics also accurately classified quality for 83% of eCLIP data sets (Extended Data Fig. 1d, e, Supplementary Text, Supplementary Fig. 11). Data sets that passed manual but not automated quality assessment were released with specific exceptions noted (Supplementary Data 8). Although we have observed that stringent IP wash conditions generally limit the recovery of indirect interactions, we note that the eCLIP experiments described here did not include visualization of protein-associated RNA and thus independent validation of eCLIP profiles through comparison with in vitro motifs and knockdown-responsive changes provides essential validation of authentic binding.

Standard CLIP-seq analyses often identify thousands to hundreds of thousands of clusters of enriched read density (Extended Data Fig. 1f, Supplementary Data 4). However, we have previously shown that requiring enrichment in IP versus paired input experiments improves specificity in identifying biologically relevant peaks by removing non-specific signal at abundant transcripts^10^. Thus, although data for all clusters identified from IP-only analysis are provided, in this study we required peaks to meet stringent criteria of enrichment relative to input (fold enrichment ≥8 and *P* ≤ 0.001). We further required that significant peaks be reproducibly identified across both biological replicates using an irreproducible discovery rate (IDR) approach (Methods, Supplementary Fig. 10c). Finally, we removed peaks that overlapped with 57 ‘blacklist’ regions (many of which contain either adaptor sequences or tRNA fragments) that show consistent artefactual signal (Supplementary Data 11). Downsampling analysis indicated that peaks were robustly detected at standard sequencing depth even in genes with low expression (transcripts per million (TPM) near or even below 1) (Supplementary Fig. 12).

**Fig. 2:**
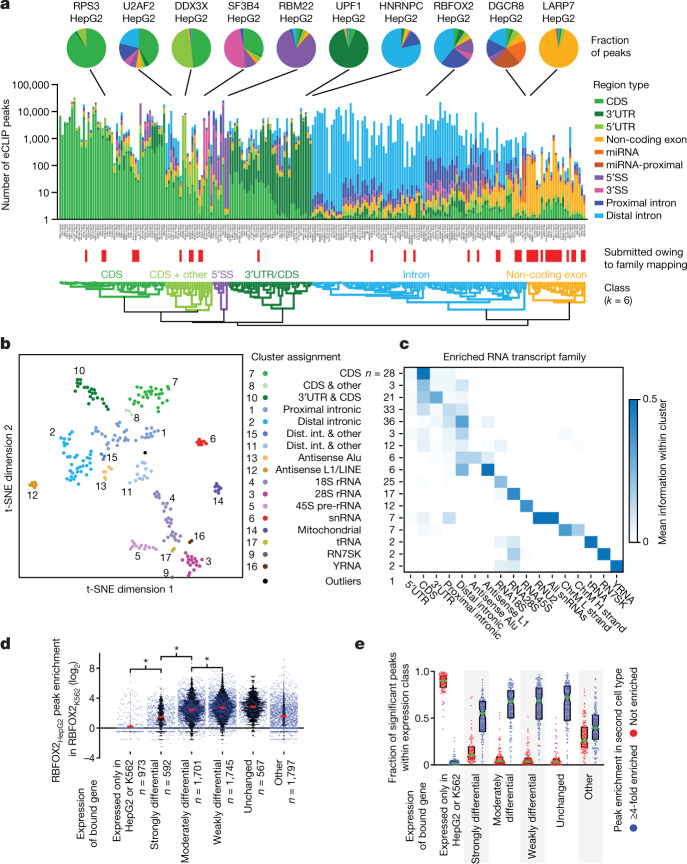
Integrated analysis of RBP–target association networks. **a**, Stacked bars indicate significant eCLIP peaks (fold enrichment ≥8, *P* ≤ 0.001, and biologically reproducible by IDR) for 223 eCLIP experiments. Number of peaks is shown on a logarithmic scale; bar heights are pseudo-coloured according to the linear fraction of peaks overlapping the indicated regions of pre-RNA, mRNA, and non-coding RNAs. Data sets were hierarchically clustered to identify six clusters based on similar region profiles (Extended Data Fig. 3a). **b**, Seventeen clusters and one outlier of RBPs based on *t*-distributed stochastic neighbour-embedding (*t*-SNE) clustering (performed in MATLAB with algorithm = exact, distance = correlation, and perplexity = 10) of unique genomic and multicopy element signal for 223 eCLIP experiments. **c**, For RBPs in clusters in **b**, heatmap indicates the average relative information for each listed RNA region or element. **d**, Each point indicates the fold enrichment in eCLIP of RBFOX2 in K562 cells (RBFOX2_K562_) for a reproducible RBFOX2 eCLIP peak in HepG2 cells (RBFOX2_HepG2_), with underlaid black histogram, separated by the difference in expression of the bound gene between K562 and HepG2 cells. Red lines indicate mean; two-sided Kolmogorov–Smirnov test. **e**, For each RBP profiled in both K562 and HepG2 cells (*n* = 73), points indicate the fraction of peaks in the first cell type associated with a given gene class that are (blue) at least fourfold enriched, or (red) not enriched (fold enrichment ≤1) in the second cell type. Boxes indicate quartiles, green lines show mean.

**Extended Data Fig. 2:**
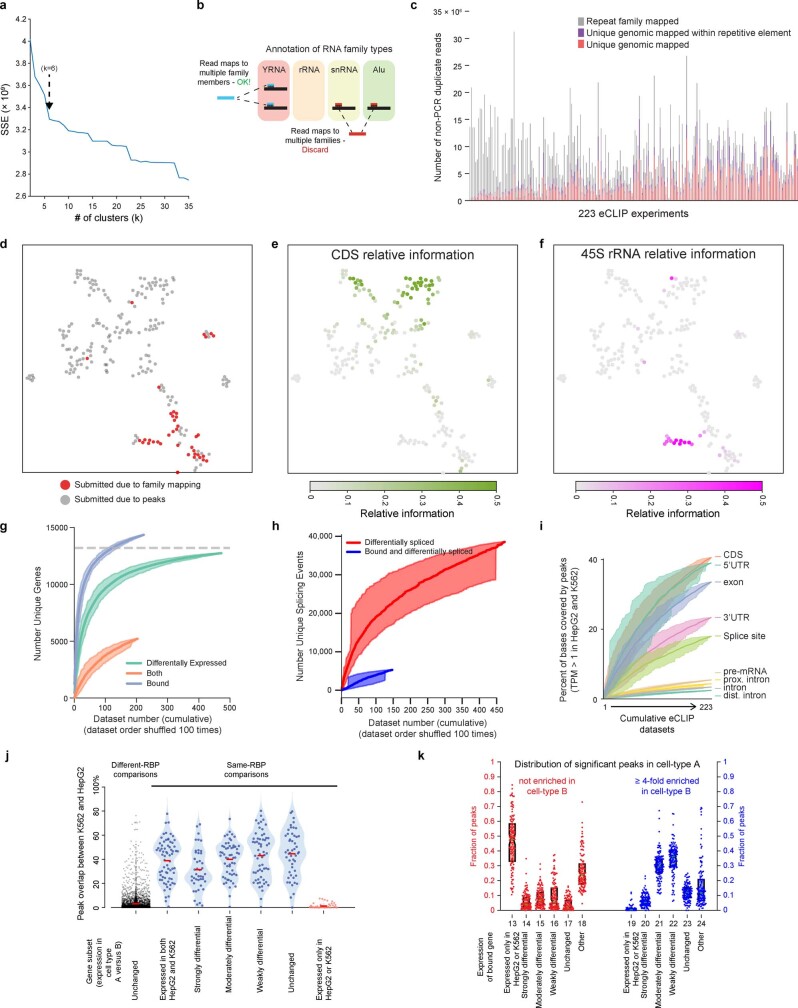
Integrated analysis of 223 eCLIP data sets identifies RBP clusters on the basis of binding patterns. **a**, The effect of cluster number on hierarchical clustering on the Euclidean distance between RBPs for the fraction of peaks overlapping each of the RNA region types as shown in Fig. 2a. For each number of clusters *k* between 2 and 35, the sum of squared error was calculated between the number of peaks annotated for each region versus the mean of all RBPs in that RBP’s cluster and summed across all RBPs. An inflection point was identified at *k* = 6 (indicated). **b**, Model of eCLIP analysis pipeline for quantification of eCLIP signal at RNA families with multiple transcript or pseudogene copies. **c**, Stacked bars indicate the number of reads from replicate 1 of all 223 eCLIP experiments, separated by whether they map uniquely to the genome (red), uniquely to the genome but within a repetitive element identified by RepeatMasker (purple), or to repetitive element families (grey). Data sets are sorted by the fraction of unique genomic reads. **d**–**f**, Each eCLIP data set is displayed as a point based on *t*-SNE clustering (Fig. 2b), with colour indicating whether the data set passed peak-based or family-mapping based quality assessment (**d**), the relative information at coding sequence (CDS) (**e**), or relative information at the 45S ribosomal RNA precursor (**f**). **g**, Means of 100 random orderings of each data type for the number of genes that were differentially expressed for all 472 KD–RNA-seq data sets (requiring FDR < 0.05 and *P* < 0.05 from DEseq analysis; Methods) (green), bound in 223 eCLIP data sets (overlapped by a IDR-reproducible peak with *P* ≤ 10^−3^ and fold enrichment ≥ 8 in IP versus input; Methods) (blue), or both bound and differentially expressed (considering 203 pairings of eCLIP and KD–RNA-seq for an RBP in the same cell type) (orange). The set of genes considered was all 57,645 genes in GENCODE v19; see Supplementary Fig. 13a, b for analyses of expressed genes only. Grey dotted line indicates the total number of expressed genes, defined as TPM > 1 in either K562 or HepG2 cells. Shaded regions indicate 10th to 90th percentiles. **h**, Means of 100 random orderings of data sets for the number of differential splicing events for all 472 RBP KD–RNA-seq experiments (including skipped exons, alternative 5′ and 3′ splice sites, retained introns, and mutually exclusive exons; requiring FDR < 0.05, *P* < 0.05, and |Δ*Ψ*| > 0.05) (red), and exons both bound by an RBP and differentially spliced upon RBP knockdown (considering 203 pairings of eCLIP and KD–RNA-seq for an RBP in the same cell type) (blue), with binding defined as a peak located anywhere between the upstream intron 5′ splice site and the downstream intron 3′ splice site. Shaded regions indicate 10th to 90th percentiles. **i**, Cumulative fraction of bases within peaks for 100 random orderings of the 223 eCLIP data sets, separated by transcript regions as indicated. Shaded region indicates 10th to 90th percentiles. See Supplementary Text and Supplementary Fig. 13c, d for additional analyses of all versus expressed genes only. **j**, Fraction of overlapping peaks identified from our standard eCLIP processing pipeline between K562 and HepG2 cells for RBPs profiled (blue or red) in both cell types, or (black) between one RBP in K562 cells and a second in HepG2 cells, for sets of genes separated by their relative expression change between K562 and HepG2 cells as follows: unchanged (fold-difference ≤ 1.2), weakly (1.2 < fold-difference ≤ 2), moderately (2 < fold-difference ≤ 5) or strongly (fold-difference > 5) differential, or cell type-specific genes (TPM < 0.1 in one cell type and TPM ≥ 1 in the other). Red line indicates mean. **k**, Each point represents one eCLIP data set compared with the same RBP profiled in the second cell type (73 total). For the set of peaks from the first cell type that are not enriched (fold enrichment < 1) in the second cell type, red points indicate the fraction that occur in genes with the indicated expression difference between HepG2 and K562 cells. Blue points similarly indicate the gene distribution of peaks that were fourfold enriched in the opposite cell type. Boxes, quartiles; green line, median.

When we overlaid peaks onto GENCODE transcript annotations, the peaks for most RBPs overlapped specific regions, consistent with previously identified functional roles of RBPs (Fig. 2a). We clustered these RBPs into six ‘RNA-type classes’ on the basis of the dominant transcript region type bound, which provided reference comparisons for later peak-based analyses (Fig. 2a, Extended Data Fig. 2a, Supplementary Data 4). Upon observing that uniquely mapped reads represented a minority of the total for many eCLIP data sets, we developed a family-aware mapping strategy that enabled us to accurately quantify relative enrichment at multi-copy elements, including gene families with multiple pseudogenes (such as ribosomal RNA or Y RNA), retrotransposons, and other repetitive elements (Extended Data Fig. 2b, c). Incorporating this approach, we observed clusters of RBPs dominated by rRNA or snRNA signal consistent with known functions, as well as clusters dominated by antisense Alu and L1/LINE signal (Fig. 2b, c, Extended Data Fig. 2d–f), in agreement with recent analysis indicating that binding to retrotransposable elements (particularly in the antisense orientation) comprises an underappreciated part of the global RBP binding landscape^17^.

## Saturation of RBP element discovery

Although most expressed genes showed differential expression and had eCLIP peaks in at least one data set, only 5,214 genes had eCLIP peaks from and were responsive to knockdown of the same RBP, suggesting that a large fraction of knockdown-responsive changes in expression result from indirect effects (Extended Data Fig. 2g, Supplementary Fig. 13a, b). Alternative splicing showed even greater variability, driven by more than 13,000 splicing changes identified upon knockdown of the RNA helicase and spliceosomal protein AQR (threefold more than any other RBP; Extended Data Fig. 2h). Considering eCLIP alone, 3.4% of expressed intronic and 33.5% of exonic sequences were covered by at least one peak (Extended Data Fig. 2i, Supplementary Text, Supplementary Fig. 13c–g), although many peaks reflected association of proteins that coat or transiently interact with RNAs, such as interaction of the RNA polymerase II component POLR2G with pre-mRNAs, rather than RNA-processing regulatory sites.

Next, we evaluated whether RBP regulation is consistent across cell types. We observed that RBFOX2 eCLIP peaks in HepG2 cells were also typically enriched in K562 cells (average enrichment 6.2-fold) if the overall target RNA expression was within a factor of five (Fig. 2d). Extending this to all 73 RBPs with eCLIP data in both cell types, most peaks in unchanging or moderately differentially expressed genes were enriched fourfold or more in the second cell type, and often overlapped a reproducible and significant (fold enrichment ≥8, *P* ≤ 0.001) peak in the other cell type (Fig. 2e, Extended Data Fig. 2j). By contrast, an average of 46.3% of RBP peaks that showed no enrichment in the second cell type occurred in genes with cell type-specific expression (threefold enrichment), whereas only 21.6% occurred in unchanging, weakly, or moderately differentially expressed genes (Extended Data Fig. 2k). Thus, these results suggest that most RBP eCLIP signal is preserved across cell types for similarly expressed genes, whereas peak discrepancies often reflect cell type-specific RNA expression rather than differential binding.

## In vitro specificity drives in vivo binding

**Fig. 3:**
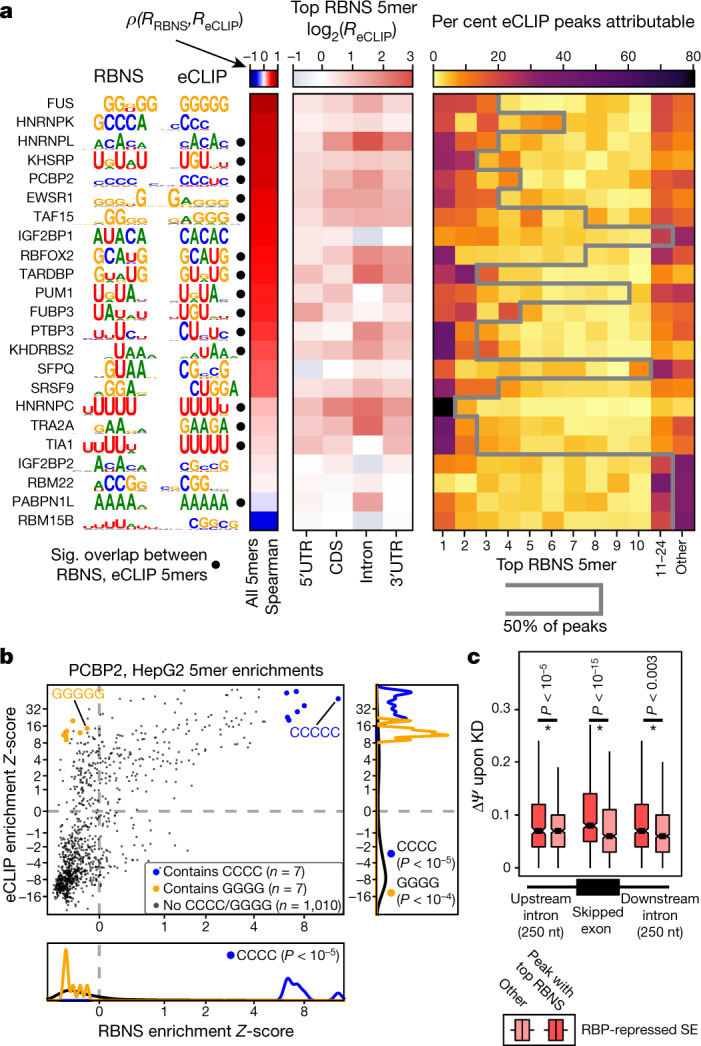
Sequence-specific binding in vivo is determined predominantly by intrinsic RNA affinity of RBPs. **a**, Left, top sequence motif of RBNS- and eCLIP-enriched 5mers ordered by decreasing correlation RBNS and eCLIP enrichments. Filled circles indicate significant RBNS and eCLIP motif overlap (hypergeometric *P* < 0.05). Left heatmap, Spearman correlation between RBNS and eCLIP enrichments for all 5mers. Centre heatmap, enrichment of the top RBNS 5mer in eCLIP peaks (*R*_eCLIP_) within different genomic regions. Right heatmap, proportion of eCLIP peaks attributed to each of the ten highest-affinity RBNS 5mers, as well as RBNS 5mers 11–24 combined. Grey line indicates the number of top RBNS 5mers required to explain more than 50% of eCLIP peaks for each RBP (maximum, 24 5mers). **b**, Comparison of PCBP2 in vivo versus in vitro 5mer enrichments, with 5mers containing CCCC and GGGG highlighted. Significance determined by one-sided Wilcoxon rank-sum test and indicated if *P* < 0.05.  The *x*- and *y*-axes are plotted on an arcsinh scale. Similar results were obtained when analysing 6mers. **c**, Comparison of splicing changes upon RBP knockdown for RBP-repressed cassette exons (skipped exons, SE) with exon peaks with RBNS motif (*n* = 368) or without RBNS (*n* = 1,758), upstream intron peaks with RBNS (*n* = 223) or without RBNS (*n* = 2,195), and downstream intron peaks with RBNS (*n* = 250) or without RBNS (*n* = 953). Boxes, 25th to 75th percentiles; notch, median; line, outliers. Significance determined by one-sided Wilcoxon rank-sum test and indicated if *P* < 0.05.

**Extended Data Fig. 3:**
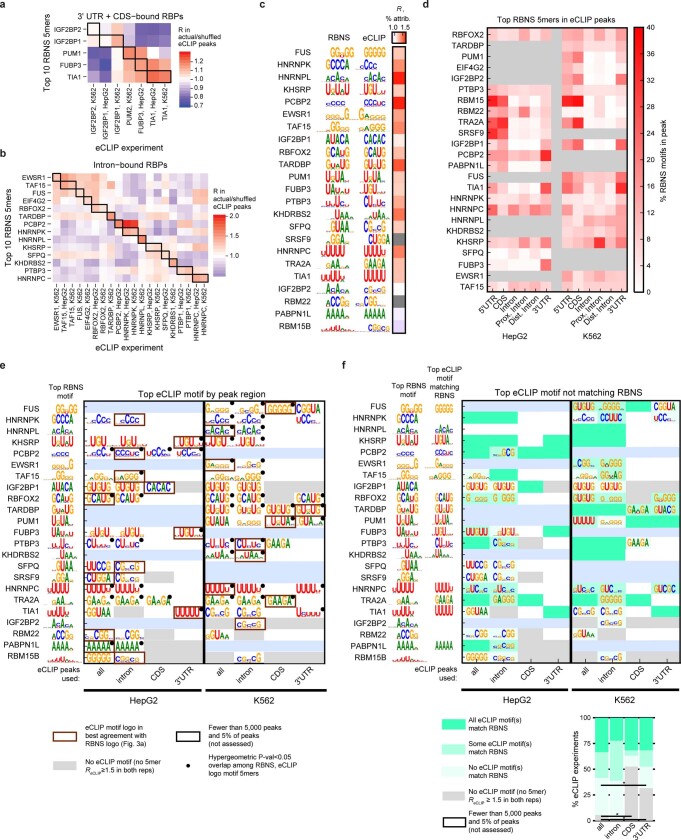
Enrichment of in vitro motifs in eCLIP peaks for different RNA types and comparison with in vivo eCLIP-derived motifs. **a**, **b**, The average enrichment (geometric mean) of the top ten RBNS 5mers for a given RBP in the peaks of an eCLIP experiment compared to shuffled eCLIP peaks, among all RBPs predominantly bound to 3′ UTR + CDS (**a**) or introns (**b**) by eCLIP. RBPs arranged by RBNS motif similarity along the *y*-axis, with corresponding RBPs between RBNS and eCLIP boxed along the diagonals. **c**, RBP order and RBNS and eCLIP motifs as in Fig. 3a. Right, ratio of the percentage of eCLIP peaks attributable to the top ten RBNS 5mers for each RBP compared to the percentage of eCLIP peaks attributable to the same ten 5mers, averaged over all other eCLIP experiments in the same RNA type class (from **a** and **b**). For 18 out of 21 RBPs, the RBNS motifs explain more (*R* > 1) of the corresponding eCLIP peaks than eCLIP peaks of proteins binding similar transcript regions (SRSF9 and RBM22, shown in grey, were excluded because there were insufficient numbers of RBPs in their type class to perform this analysis). **d**, The proportion of the top ten RBNS 5mers that fall within an eCLIP peak, separated by transcript region. RBPs arranged from top to bottom according to the proportion that fall within an eCLIP peak over all transcript regions (all motif occurrences in expressed transcripts). **e**, Top motifs derived from all eCLIP peaks as well as eCLIP peaks within intronic, CDS, and 3′UTR regions. Motifs were derived only if there were at least 5,000 peaks or 5% of total peaks in that region, averaged over the 2 eCLIP replicates. Blue boxes indicate that eCLIP was not performed in that cell line. Filled circles indicate significant overlap (*P* < 0.05 by one-sided hypergeometric test) between RBNS and eCLIP motifs. **f**, The top eCLIP motifs that do not match RBNS for the corresponding RBP (if any). The eCLIP motif was considered as matching RBNS if any of its constituent 5mers were among the RBNS *Z* ≥ 3 5mers (always using at least 10 RBNS 5mers if there were fewer than 10 with *Z* ≥ 3). Blue boxes indicate that eCLIP was not performed in that cell line. Below right, percentage of eCLIP experiments aggregated over all RBPs or cell types in each category of agreement with RBNS. Horizontal line indicates a significant difference in the proportion of a particular eCLIP–RBNS agreement category between eCLIP analysis of all peaks versus eCLIP analysis of intron, CDS, or 3′UTR peaks (*P* < 0.05 by one-sided Fisher’s exact test).

RBP binding in vivo is determined by intrinsic RNA-binding specificity and other influences, such as RNA structure and protein cofactors. To compare in vitro and in vivo specificities, we calculated the raw enrichment (*R* value) of each 5mer in RBNS-bound sequences and compared these to corresponding enrichments in eCLIP peaks (*R*_eCLIP_). We focused on 5mers because they were most robust^12^ and because most proteins analysed by RBNS contained RNA recognition motif (RRM) or hnRNP K homology (KH) domains, which bind about 3–5 bases of RNA^18,19^. Significantly enriched 5mers in vitro and in vivo were mostly in agreement, with 15 of the 23 RBPs having significant overlap (Fig. 3a, left). The top RBNS 5mer for an RBP was almost always enriched in eCLIP peaks of that RBP (Fig. 3a, centre), and RBNS motifs explained more of the corresponding eCLIP peaks than eCLIP peaks for other RBPs in the same RNA type class for 18 of 21 RBPs analysed (Extended Data Fig. 3a–c). In most cases, similar results were observed for eCLIP peaks in coding, intronic or UTR regions (Fig. 3a (centre), Extended Data Fig. 3d, e). Notably, the single most enriched RBNS 5mer occurred in 30% of peaks or more for several RBPs including SRSF9, TRA2A, RBFOX2, PTBP3, TIA1, and HNRNPC, and for most RBPs half of eCLIP peaks contained one of the top five RBNS 5mers (Fig. 3a, right). Therefore, instances of these 5mers provide candidate nucleotide-resolution binding locations, enabling the prediction of genetic variants that alter RNA processing. When two or more distinct motifs were enriched in both RBNS and eCLIP, the most enriched motif in vitro was usually also the most enriched in vivo (five out of seven cases). These observations are consistent with the idea that for RBPs that contain largely single-stranded RNA-binding domains such as those studied here, intrinsic binding specificity explains a substantial portion of in vivo binding preferences.

For slightly under half of the investigated RBPs (10 of 23), the top five RBNS 5mers explained fewer than half of eCLIP peaks. Some of these RBPs have affinities to RNA structural features or to extended RNA sequence elements that are not well represented by 5mers^12^, whereas for others, binding may be driven by interacting proteins. In some cases, RBNS revealed affinity to only a subset of the motifs that were enriched in eCLIP peaks. For example, C-rich 6mers were most enriched in the RBNS data for PCBP2 and also in PCBP2 eCLIP peaks (Fig. 3b), but a subset of eCLIP-enriched *k*mers were not enriched by RBNS (for example, G-rich 6mers, Fig. 3b). Such ‘eCLIP-only’ motifs, which were often G-, GC-, or GU-rich (Extended Data Fig. 3f), may represent RNA binding of other proteins that interact with the targeted RBP, or could represent biases in co-purification or crosslinking positions or biases in sequences near crosslink sites^20^.

**Extended Data Fig. 4:**
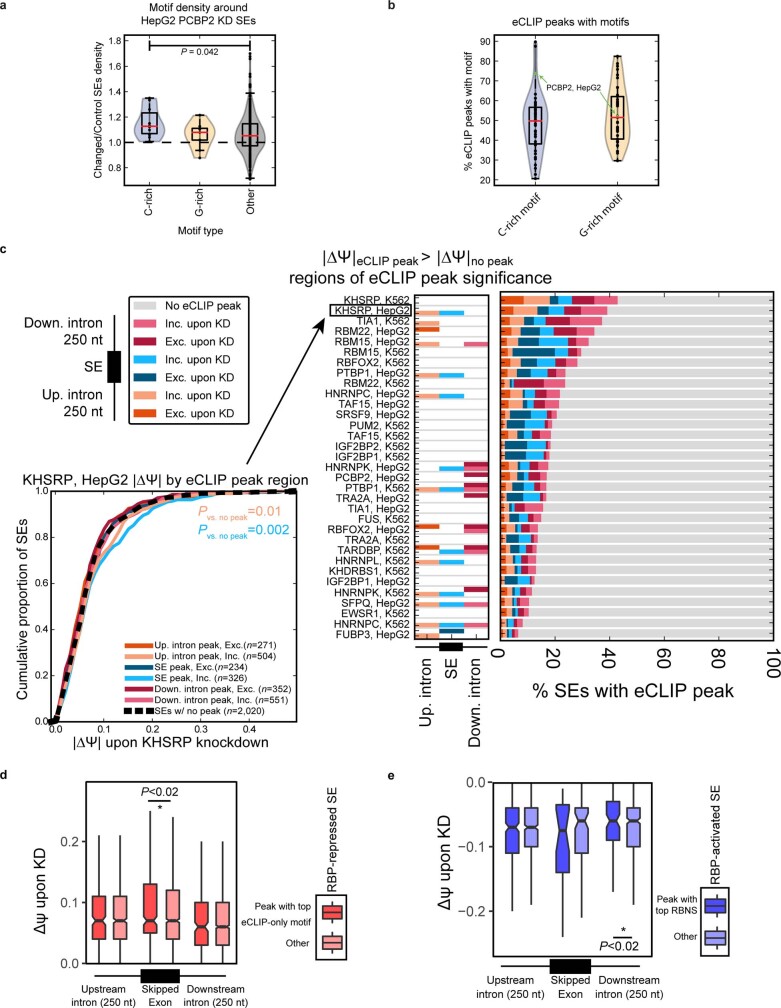
Splicing regulatory activity of RBNS+ and RBNS− eCLIP peaks. **a**, Density of 5mers in skipped exons and their flanking intronic up/downstream 100 nt in 24 changed versus control skipped exons upon PCBP2 knockdown in HepG2 cells. The ratio of changed and control frequency was computed for each 5mer with the ratio plotted as density on the *y*-axis, and 5mers were separated by C-rich (contain 4–5 Cs), G-rich (contain 4–5 Gs), or ‘other’. Significance determined by one-sided Kolmogorov–Smirnov test. Box, 25th to 75th percentiles; notch, median. **b**, Percentage of eCLIP peaks that contain a C- or G-rich motif (5mer with 4 or 5 of the respective base). PCBP2 eCLIP in HepG2 cells is noted (eCLIP with the third highest proportion of peaks with C-rich motifs; median for peaks containing G-rich motifs). Box, 25th to 75th percentiles; notch, median. **c**, Bottom left, distribution of change Δ*Ψ* upon knockdown in each of the six eCLIP+ peak region–skipped exon splicing change types compared to that of eCLIP− skipped exons for KHSRP in HepG2 cells (significant if *P* < 0.05 by one-sided Wilcoxon rank-sum test). Right, regions of significance for eCLIP+ versus eCLIP− skipped exons for each eCLIP experiment and proportion of skipped exons in each of the six eCLIP+ types for each eCLIP experiment. **d**, Same set of RBPs and corresponding eCLIP+ peak region–skipped exon splicing change types as in Fig. 3c, but separating eCLIP peaks on whether they contain the top ‘eCLIP-only’ 5mer (based on the motifs from Extended Data Fig. 3f) instead of the top RBNS 5mer. Box, 25th to 75th percentiles; notch, median; line, outliers. Significance was determined by one-sided Wilcoxon rank-sum test and indicated if *P* < 0.05. **e**, As in Fig. 3c, but shown for RBP-activated skipped exons (decreased inclusion upon RBP knockdown). Box, 25th to 75th percentiles; notch, median; line, outliers. Significance was determined by one-sided Wilcoxon rank-sum test and indicated if *P* < 0.05.

In the case of PCBP2, C-rich (RBNS) motifs but not G-rich (eCLIP-only) motifs were enriched adjacent to PCBP2-regulated exons (Extended Data Fig. 4a, b), suggesting that RBNS motifs might help to determine which eCLIP peaks correspond to factor-specific regulation. Considering RBPs with eCLIP, RBNS, and KD–RNA-seq data, eCLIP enrichment near alternative exons was associated with increased splicing changes upon knockdown for 18 out of 28 known splicing regulatory RBPs as compared to 1 out of 7 others (hypergeometric *P* < 0.05, Extended Data Fig. 4c). To explore the relationship between sequence-specific binding and regulation, we classified whether eCLIP peaks contained (RBNS+) or lacked (RBNS–) the highest-affinity RBNS motif (Methods). In exon-proximal regions, RBNS+ eCLIP peaks were associated with stronger repression of exon skipping, with an average increase of about 25% in change of exon inclusion over RBNS– peaks (Fig. 3c). Thus, eCLIP peaks that reflect sequence-specific in vitro binding appear to confer stronger regulation than other eCLIP peaks, perhaps because they represent interactions that last longer. Similar analysis of eCLIP peaks classified by the presence or absence of the top eCLIP-only 5mer yielded minimal differences in splicing regulatory activity (Extended Data Fig. 4d). Unlike RBP-repressed exons, RBP-activated exons showed only a marginally significant (*P* < 0.02) difference between RBNS+ and RBNS– peaks (in the opposite direction) in the downstream intron region and no significant difference elsewhere (Extended Data Fig. 4e). Why a stronger effect is observed for RBP-repressed than RBP-activated exons is not clear; perhaps longer-duration binding is more critical for repression than for activation of splicing.

## Functional characterization of RBP targets

**Fig. 4:**
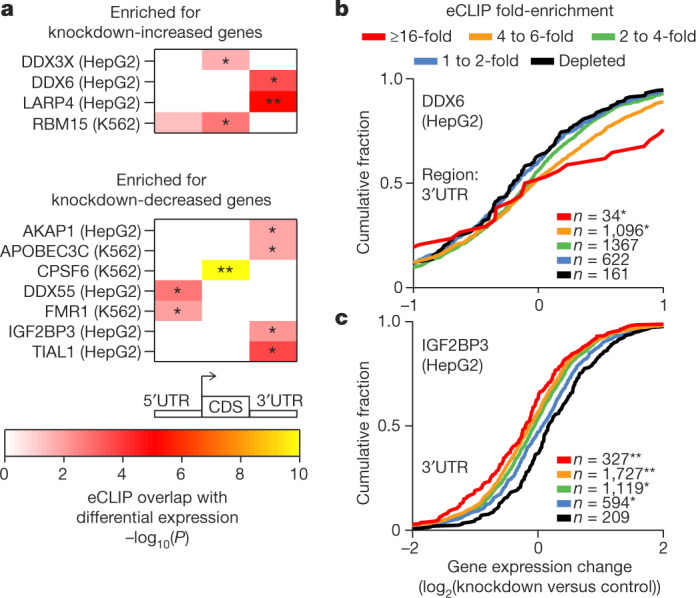
Association between RBP binding and RNA expression upon knockdown. **a**, Heatmap indicates significance of overlap between genes with regions that were significantly enriched (*P* ≤ 10^−5^ and ≥4-fold enriched in eCLIP versus input) and genes that were significantly (top) increased or (bottom) decreased (*P* < 0.05 and false discovery rate (FDR) <0.05) in RBP knockdown RNA-seq experiments. Significance determined by two-sided Fisher’s exact test or Yates’ *χ*^2^ approximation where appropriate; *P < 0.05, **P < 10^−5^ after Bonferroni correction. Shown are all overlaps meeting a *P* < 0.05 threshold; see Extended Data Fig. 5b for all comparisons. **b**, **c**, Lines indicate cumulative distribution plots of gene expression fold-change (knockdown versus control) for indicated categories of eCLIP enrichment of DDX6 in HepG2 cells (**b**), and IGF2BP3 in HepG2 cells (**c**). **P < 10^−5^, *P < 0.05; two-sided Kolmogorov–Smirnov test.

**Extended Data Fig. 5:**
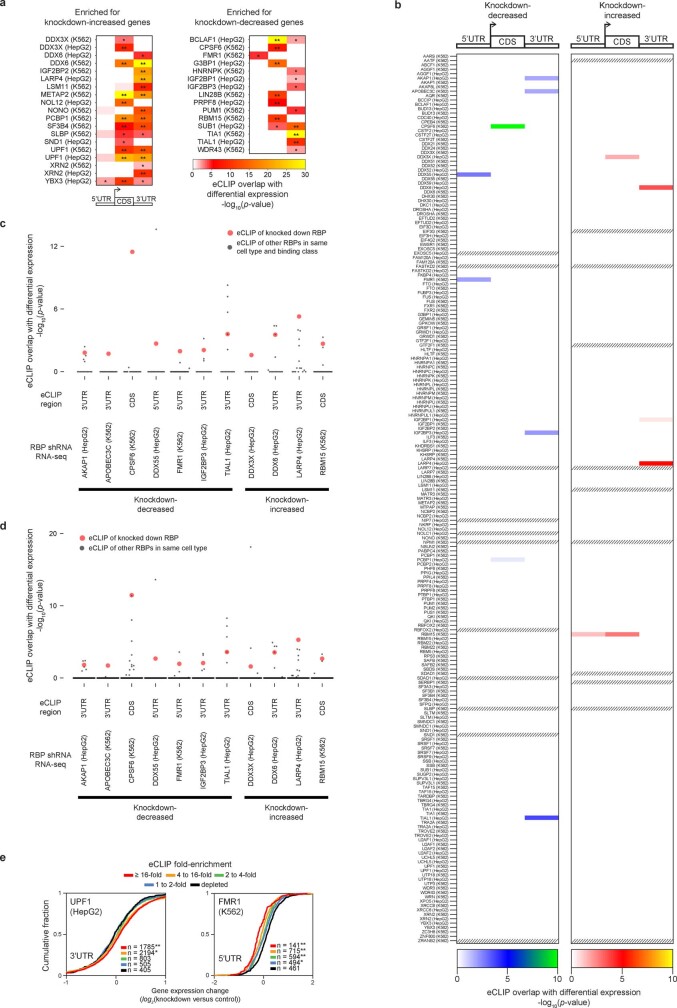
Association between RBP binding and RNA expression changes upon RBP knockdown. **a**, Heatmap indicates significance of overlap between genes with regions that were significantly enriched (*P* ≤ 10^−5^ and ≥fourfold enriched in eCLIP versus input) and genes that were significantly (top) increased or (bottom) decreased (*P* < 0.05 and false discovery rate (FDR)  < 0.05) in RBP knockdown RNA-seq experiments using DESeq analysis with no G/C content normalization (see Supplementary Methods and Supplementary Fig.  2). Significance determined by two-sided Fisher’s exact test or Yates’ *χ*^2^ approximation where appropriate; *P < 0.05, **P < 10^−5^ after Bonferroni correction. Shown are all overlaps meeting a *P* < 0.05 threshold. **b**, Colour indicates the significance of overlap between genes that were differentially expressed upon knockdown of an RBP and target genes with significant enrichment for 5′UTR, CDS, or 3′UTR regions in eCLIP of the same RBP in the same cell type. Shown are all 203 pairings of KD–RNA-seq and eCLIP performed in the same cell type. Hatched boxes indicate comparisons with fewer than ten genes altered in RNA-seq. The background gene set for each comparison was chosen by taking genes with at least ten reads in one of IP or input, and where at least ten reads would be expected in the comparison data set given the total number of usable reads. Significance was determined by two-sided Fisher’s exact test (or Yates’s *χ*^2^ test where appropriate) with no hypothesis testing correction (Methods). **c**, **d**, Red points indicate significance of overlap between eCLIP and KD–RNA-seq for the 13 significant overlaps (multiple hypothesis-corrected *P* ≤ 0.05), showing only the most significantly enriched region from **b**. Black points indicate knockdown RNA-seq data sets compared against enrichments for the same transcript region for eCLIP data sets for RBPs within the same binding type class (**c**) (as identified in Fig. 2a), or all eCLIP data sets in the same cell type (**d**). **e**, Cumulative distribution plots of gene expression fold-change for UPF1 knockdown in HepG2 (left) and FMR1 knockdown in K562 (right) for indicated categories of eCLIP enrichment. *P < 0.05, **P < 10^−5^; two-sided Kolmogorov–Smirnov test.

Analysis of the KD–RNA-seq data enables us to infer the functions of RNA elements identified by eCLIP. First, we considered significant changes in transcript abundance identified upon RBP KD–RNA-seq, as regulation of RNA stability alters steady-state mRNA levels (Supplementary Figs. 3, 4). To identify potential regulators of RNA stability, we compared genes that were differentially expressed upon RBP knockdown with eCLIP enrichment in 5′UTRs, coding sequences (CDSs), and 3′UTRs. Although comparison with standard DESeq analysis of the KD-RNA-seq indicated many instances of significant overlap with eCLIP enrichment (Extended Data Fig. 5a), we found it challenging to disentangle gene-level GC content biases in library preparation from sequence biases (including AU-rich elements) that correlate with RNA stability regulation. Thus, we performed a conservative analysis that fully removed potential GC-content bias in KD-RNA-seq fold changes using the Salmon and CQN tools (see Supplementary Information and Supplementary Fig. 2). We identified 4 RBPs that had correlation between eCLIP enrichment and increased expression upon knockdown, and 7 RBPs that had eCLIP correlation with decreased expression (Fig. 4a, Extended Data Fig. 5b). When compared with RBPs of the same binding class (Fig. 2a), the targeted RBP showed the greatest enrichment in 5 out of 11 cases and was among the top RBPs for most comparisons (Extended Data Fig. 5c, d).

RBPs that showed a correlation between eCLIP and increased target expression upon RBP knockdown included previously identified RNA decay factors (for example, DDX6; Fig. 4b). Other RBPs showed correlation between eCLIP and decreased expression upon knockdown, including IGF2BP3 and FMR1, which have previously been characterized to increase the stability of RNA targets^21,22^ (Fig.  4c, Extended Data Fig. 5e). In addition to these 11 RBPs, others such as UPF1 showed significant correlation at higher eCLIP enrichment cutoffs (Extended Data Fig.  5e), suggesting that more complex models may reveal additional overlaps.

## RBP association with splicing regulation

**Fig. 5:**
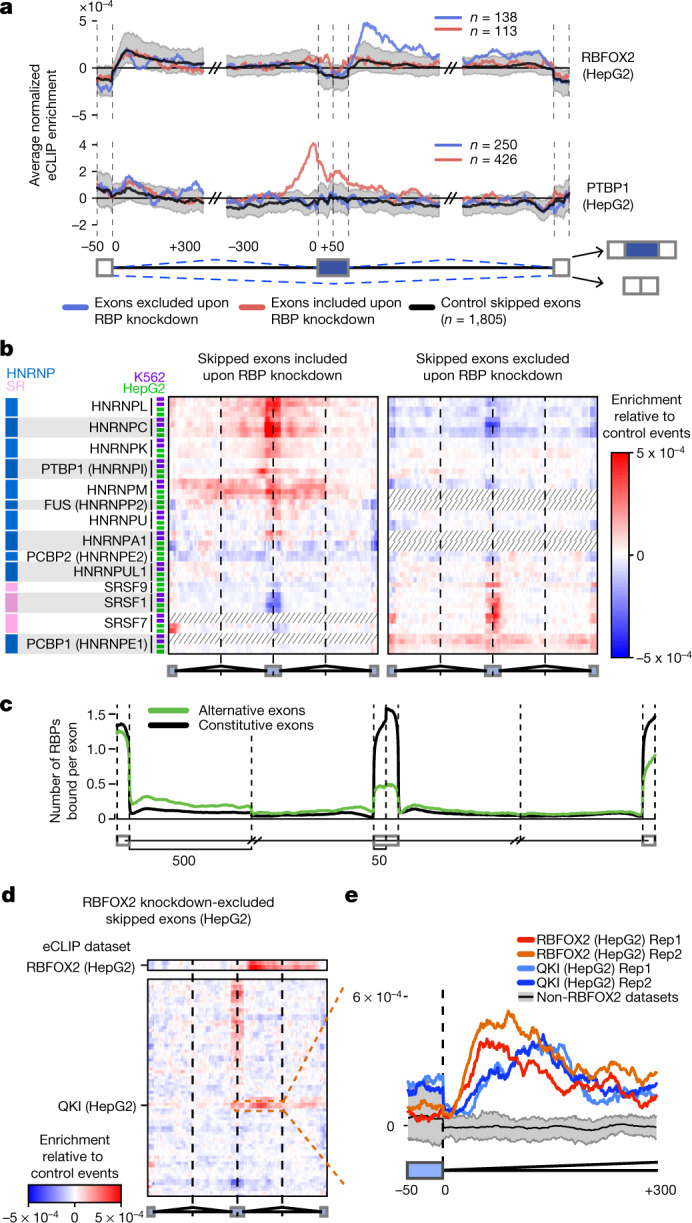
Integration of eCLIP and RNA-seq identifies splicing regulatory patterns. **a**, Normalized splicing maps of RBFOX2 and PTBP1 for skipped exons that were excluded (blue) or included (red) upon knockdown, relative to a set of ‘native’ skipped exons (nSEs) for which the inclusion rate was between 0.05 and 0.95 in controls. Lines indicate average eCLIP read density in IP versus input for indicated exon categories. Shaded area indicates 0.5th and 99.5th percentiles observed from 1,000 random samplings of native events. **b**, Heatmap indicates the difference between nSE-normalized eCLIP read density at skipped exons that were included (left) or excluded (right) upon RBP knockdown for all profiled HNRNP and SR proteins (see Extended Data Fig. 6a for all RBPs). **c**, Lines indicate the average number of RBPs with eCLIP peaks at skipped (green) versus constitutive (grey) exons and flanking introns. Spliceosome machinery RBPs were excluded from this analysis. **d**, Heatmap indicates normalized eCLIP signal at RBFOX2 knockdown-excluded exons in HepG2 cells relative to nSEs for RBFOX2 (top) and all other RBPs within the same binding class and cell type (bottom). See Extended Data Fig. 8c for all labels. **e**, Lines indicate normalized signal tracks for eCLIP replicates of RBFOX2 and QKI in downstream proximal introns. Black line, mean of 37 non-RBFOX2 data sets in the same binding class; grey, 10th to 90th percentiles.

**Extended Data Fig. 6:**
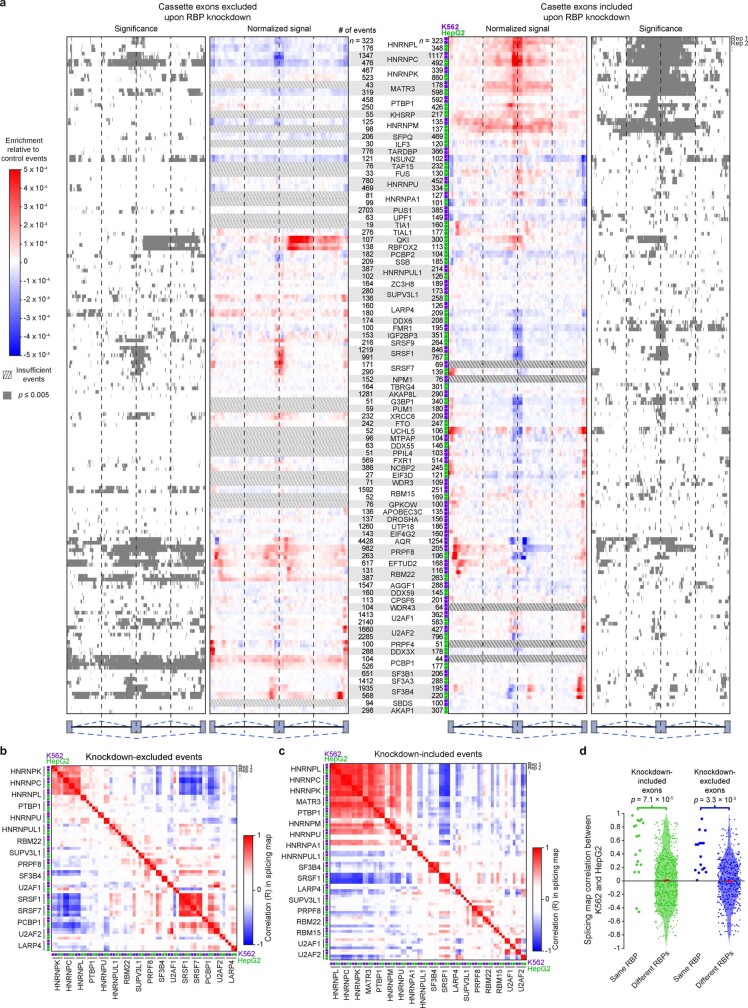
Integration of eCLIP and KD–RNA-seq to identify splicing regulatory patterns. **a**, Inner heatmap indicates the difference between normalized eCLIP read density at skipped exons that were excluded (left) or included (right) upon RBP knockdown, versus nSEs (as described in Supplementary Fig. 14). Out of 203 pairings of eCLIP and KD–RNA-seq in the same cell type (139 RBPs total), 92 pairings (72 RBPs) with at least 100 significantly included or excluded events are shown. Outer heatmap indicates positions at which the signal exceeds the 0.5–99.5% confidence interval obtained by 1,000 random samplings of the same number of events from the native skipped exon control set without multiple hypothesis testing correction. The number of RBP knockdown-altered skipped exons for each comparison is indicated. Data sets were hierarchically clustered at the RBP level, and data sets with fewer than 100 events are indicated by hatching. **b**, **c**, Heatmap indicates correlation (Pearson *R*) between splicing maps for knockdown-excluded (**b**) or knockdown-included (**c**) exons for RBPs profiled in both K562 and HepG2 cells, hierarchically clustered at the RBP level. **d**, Plot represents the distribution of Pearson correlations between splicing maps as shown in **b**, **c**, separated by whether the comparison is between the same RBP (*n* = 18 knockdown-included and 16 knockdown-excluded) or different RBPs (*n* = 612 knockdown-included and 480 knockdown-excluded comparisons, respectively) profiled in two cell types. Different RBPs are shown as smoothed histogram using a Normal kernel, and red line indicates mean. Significance was determined by two-sided Kolmogorov–Smirnov test.

**Extended Data Fig. 7:**
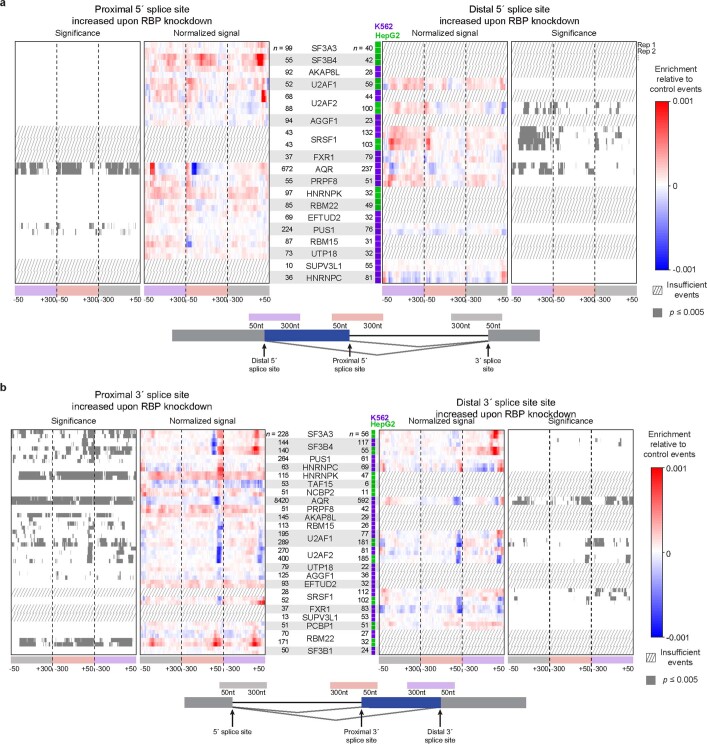
RNA maps for alternative 5′ and 3′ splice sites. **a**, **b**, Inner heatmaps indicate enrichment at RBP-responsive alternative 5′ splice site (**a**) and alternative 3′ splice site (**b**) events relative to native alternative 5′ splice site events for all RBPs with eCLIP and KD–RNA-seq data that showed a minimum of 50 significantly changing events upon knockdown. The region shown extends 50 nt into exons and 300 nt into introns. Outer heatmaps indicate positions at which the signal exceeds the 0.5–99.5% confidence interval obtained by 1,000 random samplings of the same number of events from the native alternative 5′ or 3′ splice site control sets, respectively, without multiple hypothesis correction.

Binding of an RBP to an exon (or its flanking introns) can regulate exon inclusion or exclusion or alternative 5′ or 3′ splice site usage. To consider how RBP enrichment is associated with splicing regulation, we identified all significant alternative splicing events by comparing RNA-seq data from cells in which RBPs were knocked down with data from paired non-target control cells (Supplementary Figs. 5, 6). Next, we generated an ‘RNA splicing map’ for each RBP^23^, which averages eCLIP enrichment for knockdown-responsive splicing events on a meta-exon using custom approaches to incorporate the paired input^24^ (Supplementary Fig. 14). RBFOX2 eCLIP enrichment at the downstream proximal intron correlated with exon exclusion and PTBP1 enrichment at the upstream proximal intron with exon inclusion upon RBP knockdown (Fig. 5a), consistent with previous studies of RBFOX2 and PTBP1 motif enrichment and CLIP binding^25^. Among 203 pairings of eCLIP and KD–RNA-seq performed in the same cell line (139 RBPs in total), we observed a wide variety of RNA maps for cassette exons (also referred to as skipped exons, or SE), alternative 3′ splice site events and alternative 5′ splice site events (Fig. 5a, Extended Data Figs. 6, 7). Binding of SR proteins was typically associated with decreased cassette exon inclusion upon knockdown, whereas binding of hnRNP proteins was associated with increased cassette exon inclusion upon knockdown, consistent with classical models in which SR and hnRNP proteins have antagonistic effects on splicing^26^ (Fig. 5b). When we compared data for the same RBP across cell types, we found higher splicing map correlation (particularly for knockdown-included exons) than when we looked at random pairings of RBPs (Extended Data Fig. 6b–d). Notably, many spliceosomal RBPs showed distinctive splicing map patterns, suggesting links between spliceosomal dwell time and sensitivity to knockdown that should be further explored (Extended Data Figs. 6, 7). For non-spliceosomal RBPs, RBP association was higher at intron regions bordering cassette exons than at those bordering constitutive exons that are always included, consistent with previous studies indicating that alternative events are more sensitive to modulation of splicing by individual RBPs. Notably, the upstream 5′ splice site showed even greater enrichment than the intronic regions directly flanking the alternative exon (Fig. 5c), suggesting that the 5′ splice site of the intron upstream of alternative exons represents an underappreciated region for splicing regulation.

**Extended Data Fig. 8:**
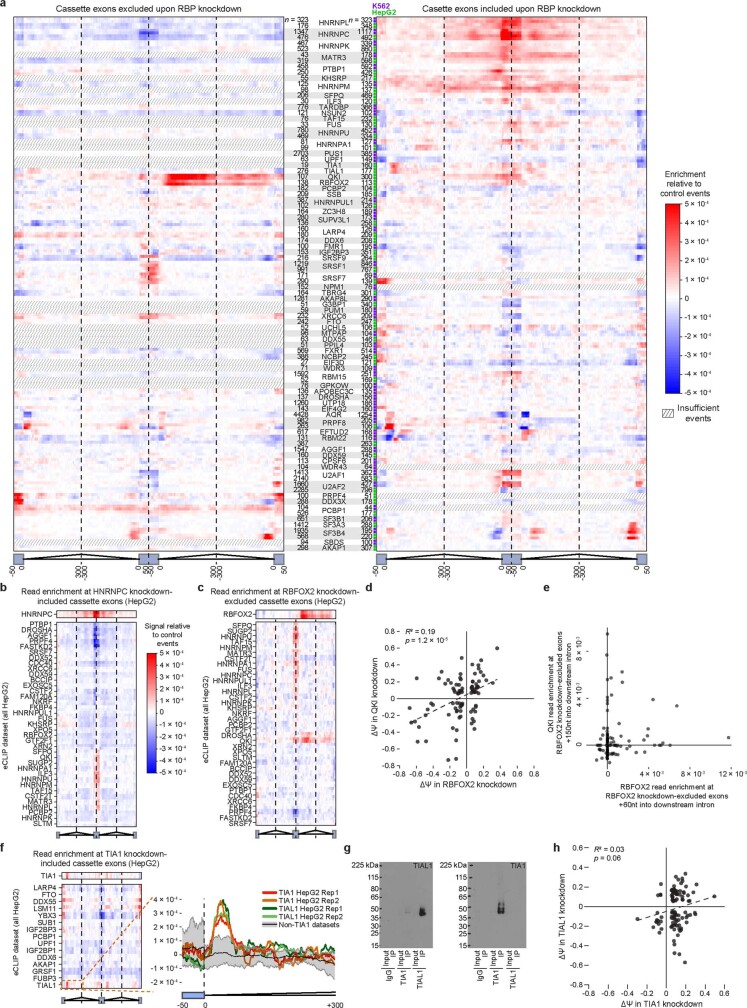
Cross-RBP splicing maps. **a**, Similar to Extended Data Fig. 6a, knockdown-altered skipped exons were identified for each RNA-seq experiment. However, for this analysis, normalized eCLIP read density at skipped exons that were excluded (left) or included (right) upon RBP knockdown versus nSEs was calculated separately for all RBPs within the same RBP class (identified in Fig. 2a). The heatmap then indicates the difference between the normalized eCLIP signal for the shRNA-targeted RBP and the mean of the normalized eCLIP signal for all other RBPs within that class. Shown are all 92 pairings of RBPs with eCLIP and KD–RNA-seq data and at least 100 included or excluded altered events, with hatching indicating data sets with fewer than 100 significantly altered events. **b**, Heatmap indicates normalized eCLIP signal at 492 HNRNPC knockdown-induced exons in HepG2 cells relative to nSEs for HNRNPC (top) and all other RBPs within the same binding class and cell type (bottom). **c**, As in **b**, for 138 RBFOX2 knockdown-excluded exons in HepG2 cells (as shown in Fig. 5d, but including all labels). **d**, Points indicate average change in Δ*Ψ* in two replicates of RBFOX2 knockdown (*x*-axis) and QKI knockdown (*y*-axis) in HepG2 cells. Shown are 93 exons that were significantly altered (*P* < 0.05, FDR < 0.1, and |ΔΨ| > 0.05) from rMATS analysis of either RBFOX2 or QKI, and had at least 30 inclusion or exclusion reads in both replicates and average |Δ*Ψ*| > 0.05 for both RBFOX2 and QKI knockdown. Significance was determined from correlation in MATLAB. **e**, For each of 138 RBFOX2 knockdown-excluded skipped exons in HepG2 cells, points indicate normalized RBFOX2 eCLIP enrichment at the +60 nt position of the downstream intron (*x*-axis) versus normalized QKI eCLIP enrichment at the +150 nt position of the downstream intron (*y*-axis). **f**, As in **b**, for 160 TIA1 knockdown-included exons in HepG2 cells. Right, black indicates mean of 15 non-TIA1 data sets in the same binding class, with the 10th–90th percentiles indicated in grey. **g**, Western blot for (left) TIAL1 and (right) TIA1 of IP performed with IgG, TIA1 (RN014P, MBLI), and TIAL1 (RN059PW, MBNL) primary antibodies. This experiment was performed once. **h**, As in **d**, for TIA1 and TIAL1 at 107 TIA1 knockdown-included exons in HepG2 cells.

As an additional control, we compared each knockdown data set against all eCLIP data sets within the same RNA type class (as defined in Fig. 2a) and observed generally similar splicing maps (Extended Data Fig. 8a). Although some individual RBPs (for example, HNRNPC) showed only same-RBP enrichment (Extended Data Fig. 8b), others indicated potential co-regulation. For example, QKI showed enriched eCLIP at RBFOX2 knockdown-excluded exons (Fig. 5d, e, Extended Data Fig. 8c), and there was significant correlation in splicing changes upon knockdown of RBFOX2 or QKI (*R*^2^ = 0.19, *P* = 1.2 × 10^−5^; Extended Data Fig. 8d), matching previous observations in SKOV3ip1 ovarian cancer cells^27^. This finding appears to reflect complex coordination, as RBFOX2 and QKI rarely have an enriched eCLIP signal for the same intron (Extended Data Fig. 8e). By contrast, TIA1 and TIAL1 show overlapping enrichment patterns at TIA1 knockdown-included exons (Extended Data Fig. 8f) despite little co-IP of the other factor (Extended Data Fig. 8g), consistent with previous studies of TIA1 and TIAL1^28^. However, exons that respond to knockdown of TIA1 and TIAL1 show little correlation in splicing change (*R*^2^ = 0.03, *P* = 0.06) (Extended Data Fig. 8h), suggesting that the regulatory effect of binding may not be shared at these sites.

## RBP association with chromatin

**Fig. 6:**
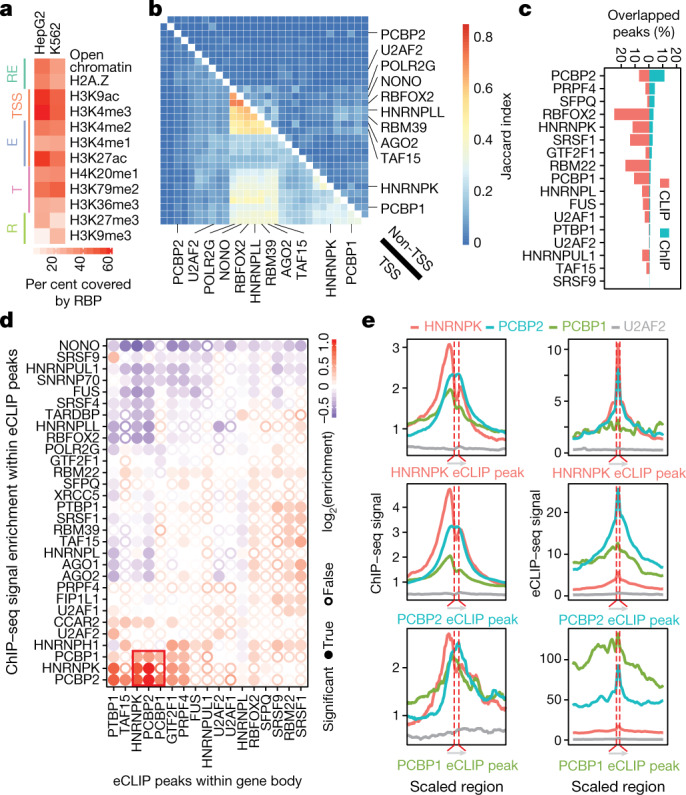
Chromatin association of RBPs and overlap with RNA binding. **a**, Overlap between RBP ChIP–seq and DNase I hypersensitive sites and various histone marks in HepG2 and K562 cells. Labels indicate marks associated with regulatory regions (RE), promoters (TSS), enhancers (E), transcribed regions (T) and repressive regions (R). **b**, Heatmap indicates the Jaccard indexes between ChIP–seq peaks of different RBPs at promoter regions (bottom left) or non-promoter regions (top right) for all HepG2 ChIP–seq data sets. See Extended Data Fig. 9b for all labels and Extended Data Fig. 9c for K562 cells. **c**, Percentage of RBP eCLIP peaks overlapped by ChIP–seq peaks (red) and percentage of RBP ChIP–seq peaks overlapped by eCLIP peaks (green) for the same RBP. RBPs are sorted by decreasing level of overlapped ChIP–seq peaks. **d**, Clustering of overlapping chromatin- and RNA-binding activities of different RBPs at non-promoter regions in HepG2. Colour indicates the degree of ChIP enrichment at eCLIP peaks relative to surrounding regions. Significant enrichments (*P* ≤ 0.001 by two-sided Wilcoxon rank-sum test with no multiple comparison correction) are indicated by filled circles. **e**, Cross-RBP comparison of chromatin and RNA-binding activities in HepG2 cells. Left, ChIP–seq density of indicated RBPs around HNRNPK, PCBP2 or PCBP1 eCLIP peaks. Right, eCLIP average read density of indicated RBPs around HNRNPK, PCBP2 or PCBP1 eCLIP peaks.

**Extended Data Fig. 9:**
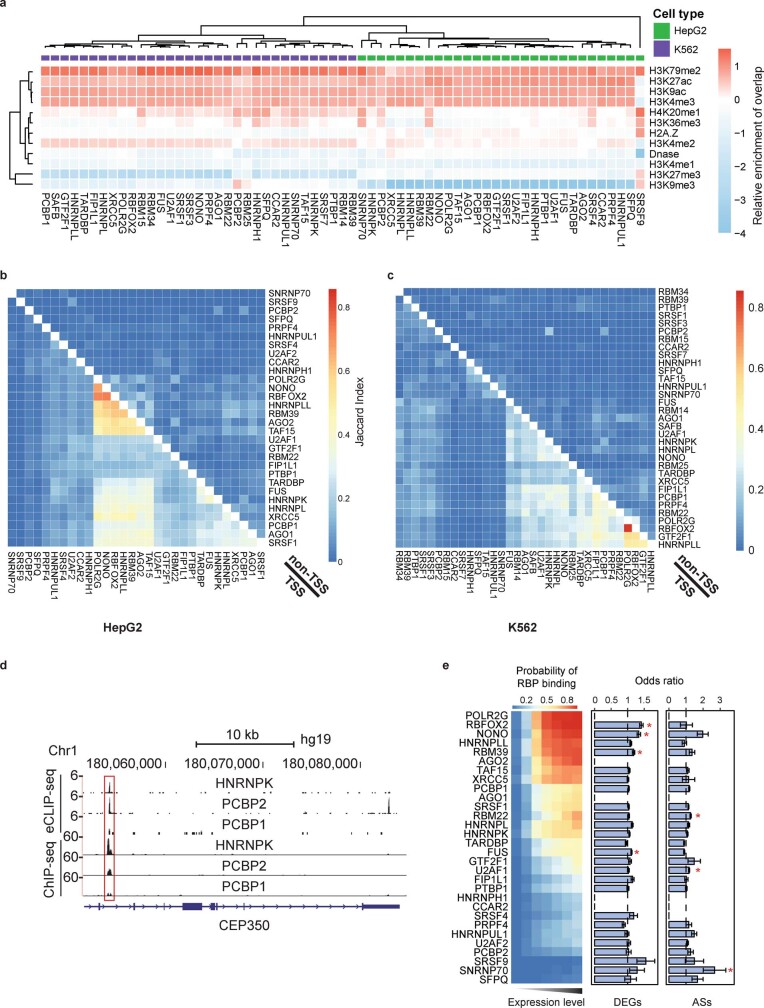
Comparison between RBP DNA and RNA association. **a**, Relative enrichment of overlap between RBP ChIP–seq peaks and peaks for indicated histone modifications, column-normalized by ‘scale’ in the R heatmap function. **b**, **c**, Jaccard indexes between ChIP–seq peaks of different RBPs at promoter regions (bottom left) or non-promoter regions (top right) are displayed as heatmaps for HepG2 (**b**) and K562 cells (**c**). **d**, A representative genomic region showing eCLIP and ChIP–seq signal for HNRNPK, PCBP2 and PCBP1 proteins in HepG2 cells. One replicate is shown; similar results were observed in a second biological replicate. **e**, Left, heatmap indicates the fraction of genes (extended 500 nt upstream of the TSS and 500 nt downstream of the TTS) overlapped by a ChIP–seq peak for each RBP for the set of genes in seven bins of increasing gene expression from RNA-seq (*x*-axis) in HepG2 cells. Middle, right, bars indicate the mean odds ratio for overlap between RBP ChIP–seq peak presence and differentially expressed genes (middle) or significant alternative splicing changes (right) upon knockdown of the same RBP relative to 100 random samplings of genes with similar expression levels. *P < 0.05 as determined by 100 random samplings of genes with similar expression levels, with no adjustment for multiple hypotheses.

Epigenetic marks affect RNA processing through co-transcriptional deposition of splicing regulators, and regulatory RNAs interact with chromatin and coordinate the regulation of epigenetic and transcriptional states^13,14^. To explore the association of specific RBPs with DNA, we performed ChIP–seq to survey 58 nuclear RBPs in HepG2 cells and 45 RBPs in K562 cells for their association with DNA. Thirty (52%) of the RBPs profiled in HepG2 cells and 33 (64%) in K562 cells showed a reproducible ChIP–seq peak, with at least 200 (up to more than 50,000) peaks (Supplementary Data 7). These RBPs belong to a wide range of functional categories, including SR and hnRNP proteins, spliceosomal components and RBPs considered to function as transcription factors, such as POLR2G and GTF2F1. With respect to established chromatin features, RBP ChIP–seq peaks showed greater overlap at euchromatin than at heterochromatin, especially at gene promoters, with variability among individual RBPs (Fig. 6a, Extended Data Fig. 9a). However, when we directly compared ChIP–seq peaks across RBPs we saw little overlap, with high concordance observed only for a small number of specific RBP pairs (Fig. 6b, Extended Data Fig. 9b, c). Collectively, these RBPs occupied about 30% of all DNase-hypersensitive or open chromatin regions and about 70% of annotated gene promoters in both cell types, which suggests that there are broad interconnections between RBPs and actively transcribed regions in the human genome.

Next, we queried the degree to which DNA targets identified from ChIP–seq and RNA targets identified by eCLIP overlapped for the same RBP, and observed an average overlap of only 6% of eCLIP peaks and 2.4% of ChIP–seq peaks (Fig. 6c, Supplementary Data 12). However, higher overlap was observed for a limited set of RBPs including the previously characterized DNA polymerase II-interacting splicing regulator RBFOX2^29^. At non-promoter regions, few RBPs displayed overlap between their ChIP and eCLIP signals, suggesting that the ChIP signal reflects interactions with DNA or DNA-binding proteins independent of direct RNA binding for most RBPs (Fig. 6d). However, we observed an association between the poly(rC) binding proteins HNRNPK and PCBP1/2. These RBPs share a common evolutionary history and domain composition but perform different functions^30^ and showed overlap in ChIP–seq and eCLIP peaks within gene bodies (Fig. 6d, Extended Data Fig. 9d). The ChIP–seq signals for PCBP1, PCBP2, and HNRNPK (but not U2AF2) were typically centred around eCLIP peaks, although for HNRNPK (and to a lesser degree PCBP1) they had a slight shift upstream of the eCLIP peak, which could reflect a specific topological arrangement of these potential RBP complexes on chromatin in a manner that depends on the direction of transcription (Fig. 6e). Thus, although ChIP–seq signals for many RBPs may simply reflect pre- or co-transcriptional association at promoter regions, a subset shows overlaps between both DNA and RNA targets within gene bodies that are likely to reflect distinct mechanisms of recruitment. The ChIP–seq targets of a limited number of RBPs also showed significant enrichment for genes that show differential expression or alternative splicing upon RBP knockdown, suggesting that the association of RBPs with chromatin may also be linked to downstream RNA processing (Extended Data Fig. 9e).

## RBP regulatory features in subcellular space

**Fig. 7:**
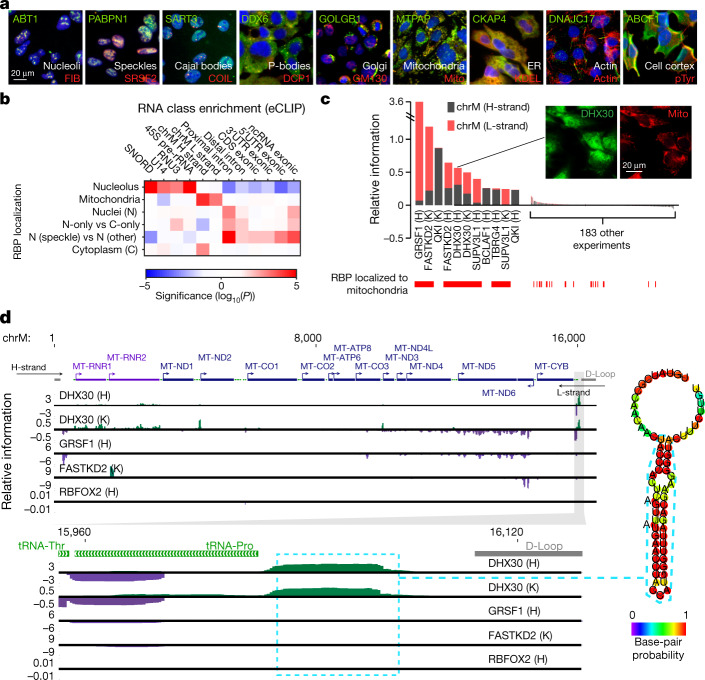
Subcellular localization of RBPs and links to transcriptome binding and regulation. **a**, Examples of RBPs (green) co-localized with nine investigated markers (red). RBPs were imaged at five or more sites per co-labelling marker with twelve co-labelled markers in total, and representative images are shown. **b**, For localization patterns with known localized RNA classes, heatmap indicates significance (from one-sided Wilcoxon rank-sum test) comparing eCLIP relative information for the indicated RNA class (*y*-axis) for RBPs with versus without the indicated localization (*x*-axis). **c**, Bars indicate eCLIP relative information content (IP versus input) for mitochondria H-strand (grey) or L-strand (red). RBPs with mitochondrial localization in HepG2 cells are indicated in red. Inset shows immunofluorescence imaging for DHX30 (representative of ten sites imaged). **d**, Genome browser tracks indicate eCLIP relative information content along the mitochondrial genome (top) or a roughly 300-nt region for indicated RBPs (bottom). Inset shows RNA secondary structure prediction (RNAfold) for the indicated region. Tracks are shown for replicate 1; eCLIP and KD–RNA-seq were performed in biological duplicate with similar results.

**Extended Data Fig. 10:**
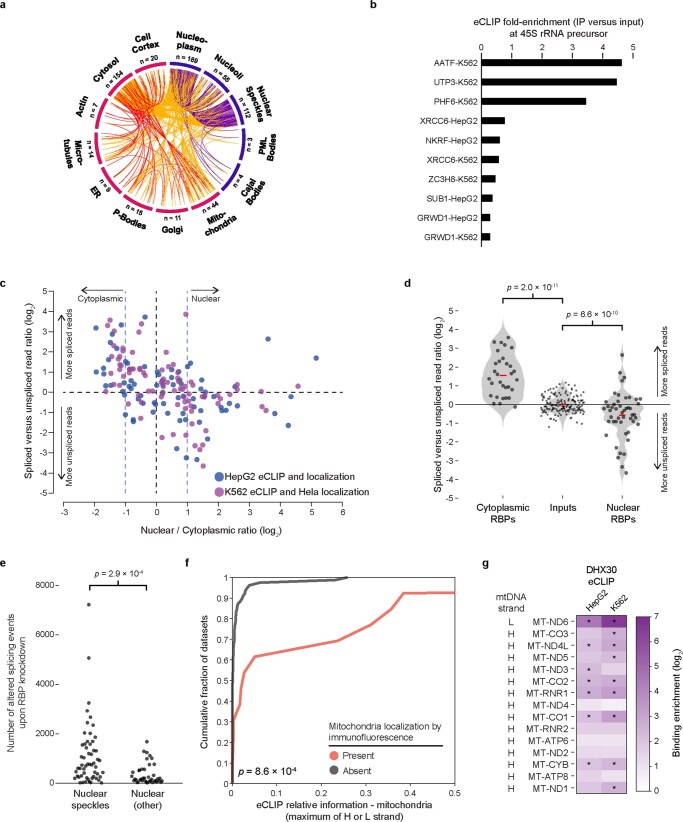
eCLIP binding patterns in subcellular space. **a**, Circos plot with lines indicating co-observed localization patterns (red, within cytoplasm; purple, within nucleus; orange, between cytoplasm and nucleus). **b**, Fold enrichment for the 45S ribosomal RNA precursor observed for eight RBPs with eCLIP data, nucleolar localization observed in immunofluorescence imaging, and no human RNA processing function identified in literature searches. **c**, Points indicate nuclear versus cytoplasmic ratio from immunofluorescence imaging (*x*-axis) versus ratio of spliced versus unspliced exon junction reads (*y*-axis), normalized to paired input. RBPs profiled by eCLIP and immunofluorescence in HepG2 cells are indicated in blue, and RBPs profiled by eCLIP in K562 cells (in purple) were paired with immunofluorescence experiments performed in Hela cells. eCLIP data shown are from replicate 1. **d**, As in **c**, with RBPs separated into nuclear (nuclear:cytoplasmic ratio ≥ 2; *n* = 48) and cytoplasmic (nuclear:cytoplasmic ratio ≤ 0.5; *n* = 31) RBPs along with inputs (*n* = 160). Significance was determined by two-sided Kolmogorov–Smirnov test. Red line indicates mean, and violin plot indicates density of data sets (with kernel smoothing). eCLIP data shown are from replicate 1. **e**, Points indicate the number of differential splicing events observed upon knockdown of each RBP, separated by the presence or absence of localization in nuclear speckles (left, *n* = 56) or nuclear but not nuclear speckles (right, *n* = 41). Significance was determined by two-sided Kolmogorov–Smirnov test. **f**, Cumulative distribution curves indicate total relative information content for the mitochondrial genome for RBPs with mitochondrial localization by immunofluorescence (red, *n* = 13) and all other RBPs (grey, *n* = 78). Significance was determined by two-sided Kolmogorov–Smirnov test. **g**, Heatmap indicates DHX30 eCLIP enrichment across all exons for all mitochondrial protein-coding and rRNA transcripts.*Significant eCLIP signal (fold enrichment ≥ 4 and *P* ≤ 0.00001 in IP versus input determined by two-sided Fisher’s exact test (or Yates’s *χ*^2^ test where appropriate) with no hypothesis testing correction; Methods). eCLIP data are shown for replicate 1; a second replicate showed similar enrichment patterns.

The subcellular localization of each RBP is important to interpret its biological function, as RNA processing occurs at multiple phase- and membrane-separated locations. Our systematic immunofluorescence imaging screen revealed diverse localization patterns (Fig. 7a), with most RBPs being associated with multiple structures in the nucleus and cytoplasm (Extended Data Fig. 10a). Considering organelles with known roles in processing specific types of RNA, localization of RBPs to nucleoli corresponded with eCLIP enrichment at 45S precursor rRNAs and small nucleolar RNAs, to mitochondria with enrichment at mitochondrial RNAs, and to nuclear speckles with enrichment at proximal intronic regions, confirming the link between localization and RNA targets (Fig. 7b). Nucleolar RBPs included 18 factors known to be involved in rRNA processing, such as BOP1, UTP18, and WDR3. Notably, 15 additional RBPs with no annotated human RNA-processing function showed nucleolar localization (Supplementary Table 1). Three of these showed an enriched eCLIP signal at the 45S rRNA: AATF and PHF6, which both showed rRNA-processing defects in a large-scale screening effort^31^, and UTP3, a human orthologue of the yeast rRNA processing factor SAS10 (Extended Data Fig. 10b). Similarly, in the nucleus, 14 out of 18 RBPs (78%) with at least fivefold enrichment for one or more small nuclear RNAs exhibited nuclear speckle localization, whereas only 51% of all RBPs with both eCLIP and immunofluorescence data in HepG2 cells colocalized with speckles (*P* = 0.016, Fisher’s exact test). We also observed increased eCLIP signal at unspliced transcripts for nuclear RBPs versus spliced transcripts for cytoplasmic RBPs (Extended Data Fig. 10c, d), and analysis of splicing changes associated with RBP depletion revealed that speckle-localized RBPs affected more splicing events than did non-speckle associated proteins (Extended Data Fig. 10e), consistent with key roles of nuclear speckles in the organization and regulation of the splicing machinery^32^.

Focusing on localization to specific cytoplasmic organelles, 42 RBPs exhibited localization to mitochondria, an organelle with unique transcriptional and RNA processing regulation. These mitochondrial-localized RBPs shared high overlap with RBPs with significant eCLIP enrichment on mitochondrial RNAs on the heavy (H) strand (QKI, TBRG4), the light (L) strand (GRSF1, SUPV3L1), or both strands (FASTKD2, DHX30), and mitochondrial localization by immunofluorescence was generally associated with significantly increased eCLIP enrichment on mitochondrial RNAs (Fig. 7b–d, Extended Data Fig. 10f). Next, we focused on DHX30, which is essential for proper mitochondrial ribosome assembly and oxidative phosphorylation^33^. As well as being associated with many mitochondrial transcripts, consistent with previous data from RNA IP and sequencing (RIP-seq)^33^ (Extended Data Fig. 10g), DHX30 was enriched at an unannotated H-strand region downstream of all annotated genes that has strong potential to form a stem–loop structure (Fig. 7d). As the termination signal for mitochondrial H-strand transcription is unknown, it is tempting to speculate that this site could mark such a signal. These examples illustrate how intracellular localization of RBPs, in combination with binding and loss-of-function data, can aid the inference of post-transcriptional regulation in different cellular compartments and organelles.

## Discussion

To our knowledge, this study represents the largest effort to date to systematically study the functions of human RBPs using integrative approaches. The resulting catalogue of functional RNA elements substantially expands the repertoire of known regulatory components encoded in the human genome. Although DNA binding proteins mostly influence gene expression levels, the functions of RBPs encompass a broader range of activities that expand transcriptome and proteome complexity, extending outside the nucleus and into the cytoplasm and organelles and contributing to multiple paths by which RNA substrates are altered (splicing, RNA editing or modification, RNA stability, localization and translation). We have demonstrated the effectiveness of combining in vivo maps of RNA-binding sites of 150 RBPs identified using eCLIP with orthogonal approaches, such as in vitro evaluation of RNA affinity for the same RBPs, chromatin association by ChIP–seq, and functional assessment of transcriptome changes by RBP depletion and RNA-seq. At the molecular level, we have confirmed that the in vivo and in vitro preferences of RBPs are highly correlated, and show that eCLIP peaks containing motifs that reflect intrinsic RNA affinity are more predictive of regulation than eCLIP peaks alone. We have confirmed, using unbiased genome-wide analyses, that SR and hnRNP proteins have broadly antagonistic effects on alternative splicing, and we have found evidence that the upstream 5′ splice sites of cassette exons have a larger role in splicing regulation than is generally appreciated. We have also implicated an RNA structure bound by an RBP in the processing of mitochondrial transcripts, and elucidated new RNA splicing maps for many RBPs. Furthermore, our data provide, to our knowledge, the first systematic investigation of chromatin-associated gene regulation and RNA processing at the level of RBP–nucleic acid interactions. At the cellular level, immunofluorescence analysis using our extensive repository of RBP-specific antibodies places these molecular interactions within particular subcellular contexts. We have confirmed that many RBPs are localized to nuclear speckles, mitochondria and other compartments, and have identified many new proteins that reside at these sites, emphasizing the necessity of localization data for interpreting RBP–RNA regulatory networks.

We have surveyed the in vivo binding patterns of 150 RBPs, comprising roughly 10% of human proteins that have been predicted to interact directly with RNA. We expect that the data reported here will provide a useful framework upon which to build analyses of other aspects of RNA regulation, such as microRNA processing^34^, RNA editing^35^, modifications such as pseudouridylation and m_6_A methylation, and translation efficiency. As we and others continue to embark on comprehensive characterization of functional RNA elements for remaining RBPs and across various cell-types and conditions, functional validation of these elements at large-scale using CRISPR–Cas9 genome-editing^36^, RNA modulation, and other technologies will become increasingly essential to study the functional roles these elements have in cellular and organismal phenotypes.

## Methods

### Cell lines

Cell lines were purchased from ATCC and were not formally authenticated, but confirmation of expected gene expression patterns were performed for RNA-seq and eCLIP experiments. Cell lines were routinely tested for mycoplasma contamination (MycoAlert, Lonza).

### RNA-binding protein annotations and domains

RBPs were chosen from a previously described list of 1,072 known RBPs, proteins containing RNA-binding domains, and proteins characterized as being associated with polyadenylated RNA, based on the availability of high-quality antibodies^9^. Annotation of RBP function was performed by integration of published literature, with manual inspection of references for less well-established annotations. Annotation of RNA-binding domain presence was determined by UniProt Domain Descriptions, and a database of cell-essential genes was obtained from published high-throughput CRISPR screening efforts^37^.

### eCLIP

#### Experimental methods

Antibodies for eCLIP were pre-screened using a set of defined metrics^9^. A ‘biosample’ of HepG2 or K562 cells was defined as a batch of cells starting from a single unfrozen stock, passaged for less than 30 days under standard ENCODE reference conditions, and validated for high viability and non-confluence at the time of crosslinking. All cells within a biosample were pooled and UV crosslinked on ice at 400 mJoules/cm^2^ with 254 nm radiation. The biosample was then split into 20-million-cell aliquots for eCLIP experiments.

eCLIP experiments were performed as previously described in a detailed standard operating procedure^10^, which is provided as associated documentation with each eCLIP experiment on the ENCODE portal (https://www.encodeproject.org/documents/fa2a3246-6039-46ba-b960-17fe06e7876a/@@download/attachment/CLIP_SOP_v1.0.pdf). In brief, 20 million crosslinked cells were lysed and sonicated, followed by treatment with RNase I (Thermo Fisher) to fragment RNA. Antibodies were pre-coupled to species-specific (anti-rabbit IgG or anti-mouse IgG) Dynabeads (Thermo Fisher), added to lysate, and incubated overnight at 4 °C. Prior to IP washes, 2% of sample was removed to serve as the paired input sample. For IP samples, high- and low-salt washes were performed, after which RNA was dephosphorylated with FastAP (Thermo Fisher) and T4 PNK (NEB) at low pH, and a 3′ RNA adaptor was ligated with T4 RNA ligase (NEB). Ten per cent of IP and input samples were run on an analytical PAGE Bis-Tris protein gel, transferred to PVDF membrane, blocked in 5% dry milk in TBST, incubated with the same primary antibody used for IP (typically at 1:4,000 dilution), washed, incubated with secondary HRP-conjugated species-specific TrueBlot antibody (Rockland), and visualized with standard enhanced chemiluminescence imaging to validate successful IP. Ninety per cent of IP and input samples were run on an analytical PAGE Bis-Tris protein gel and transferred to nitrocellulose membranes, after which the region from the protein size to 75 kDa above protein size was excised from the membrane, treated with proteinase K (NEB) to release RNA, and concentrated by column purification (Zymo). Input samples were then dephosphorylated with FastAP (Thermo Fisher) and T4 PNK (NEB) at low pH, and a 3′ RNA adaptor was ligated with T4 RNA ligase (NEB) to synchronize with IP samples. Reverse transcription was then performed with AffinityScript (Agilent), followed by ExoSAP-IT (Affymetrix) treatment to remove unincorporated primer. RNA was then degraded by alkaline hydrolysis, and a 3′ DNA adaptor was ligated with T4 RNA ligase (NEB). qPCR was then used to determine the required amplification, followed by PCR with Q5 (NEB) and gel electrophoresis to size-select the final library. Libraries were sequenced on the HiSeq 2000, 2500, or 4000 platform (Illumina). Each ENCODE eCLIP experiment consisted of IP from two independent biosamples, along with one paired size-matched input (sampled from one of the two IP lysates before IP washes).

### Experimental quality control

eCLIP experiments for the ENCODE project were performed using two biological replicates, paired with a size-matched input control subsampled from one of the two replicate samples (Extended Data Fig. 1a). Prior to sequencing, two metrics were used for assessing the quality of eCLIP experiments: successful IP of the desired RBP, and successful library generation and sequencing.

Successful IP of the targeted RBP was assayed by IP-western blot analysis. This prerequisite first requires the identification of a RBP-specific IP-grade antibody, which was previously addressed by screening over 700 antibodies to identify 438 ‘IP-grade’ antibodies against 365 RBPs in K562 cells^9^. Using these and other RBP antibodies validated by the RNA community, 488 eCLIP experiments were performed in K562 and HepG2 cell lines, yielding successful IP during the eCLIP procedure for 400 (82%). Fifty-one out of 270 (19%) and 37 out of 218 (17%) experiments gave failed IP-western blot results in K562 or HepG2 cells, respectively, indicating either potential sensitivity to enzymatic steps and additional buffer exchanges performed during the eCLIP procedure, or a lack of expression in HepG2 cells (Extended Data Fig. 1b, c). IP-western blot images are provided for each ENCODE eCLIP experiment as part of the antibody metadata available at https://www.encodeproject.org.

Failure to obtain high-quality amplified libraries from both replicates can indicate a failed experiment, lack of RNA binding, or lack of RBP–RNA crosslinking. First, 15 experiments (4%) that generated adaptor-only sequencing libraries in either replicate were abandoned. Next, an extrapolated PCR cycles required (eCT) metric was used to quantify library yield^10^. The previous eCT metric using twofold amplification per PCR cycle was modified to an accurate eCT (a-eCT) using 1.84-fold amplification per cycle on the basis of analysis of the eCLIP data resource (Supplementary Text, Supplementary Fig. 9). Thirty-six experiments that showed lower a-eCT than the average of IgG control experiments and showed no significant binding in low-depth sequencing were abandoned, leaving 349 data sets for analysis (Extended Data Fig. 1c).

### Data processing and peak identification

Processing of raw eCLIP sequencing data is complex, as adaptor sequences, double-adaptor ligation products, retrotransposable elements and other multi-copy sequences, PCR duplicates, and underlying differences in RNA abundances all contribute to false negatives and false positives at both the read mapping and peak identification stages. To address these issues, a rigorous standard eCLIP processing and analysis pipeline was developed and previously published^10^ and is provided (including description of steps as well as commands run) as a ‘Pipeline protocol’ attached to each eCLIP data set available on the ENCODE website at https://www.encodeproject.org/documents/3b1b2762-269a-4978-902e-0e1f91615782/@@download/attachment/eCLIP_analysisSOP_v2.0.pdf (Supplementary Fig. 10a). See Supplementary Text for additional details.

To identify reproducible and significantly enriched peaks across biological replicates, a modified IDR method was used (Supplementary Text, Supplementary Fig. 10). Unless otherwise noted, the final set of reproducible and significant peaks was identified by requiring that the replicate-merged peak meet an IDR cutoff of 0.01 as well as *P* ≤ 0.001 and fold enrichment ≥8 (using the geometric mean of log_2_(fold enrichment) and –log_10_(*P*) between the two biological replicates). Finally, 57 ‘blacklist’ regions were identified that were common artefacts across multiple data sets and lacked normal peak shapes (manual inspection indicated these often contain either adaptor sequences or tRNA fragments; Supplementary Data 11). IDR peaks that overlapped these blacklist regions were removed to yield the final set of reproducible peaks used in all analyses in this manuscript (unless otherwise indicated) (Supplementary Data 4).

Annotation of peaks was based on overlap with GENCODE v19 transcripts. If a peak overlapped multiple annotation types within a single annotated gene (across one or several isoform annotations), the peak annotation was chosen in the following priority order: tRNA, miRNA, miRNA-proximal (within 500 nt), CDS, 3′UTR, 5′UTR, 5′ splice site (within 100 nt of exon), 3′ splice site (within 100 nt of exon), proximal intron (within 500 nt of splice site region), distal intron (further than 500 nt from the splice site region), followed by noncoding exonic. If the peak overlapped multiple gene annotations, the final annotation was chosen as follows: tRNA, miRNA, CDS, 3′UTR, 5′UTR, miRNA-proximal, noncoding exonic, 5′ splice site, 3′ splice site, proximal intron, distal intron. To identify RBP clusters, the fraction of peaks annotated to each class out of the total number of peaks was calculated, and hierarchical clustering was performed in MATLAB (2018a) using correlation distance and average linkage. Clusters were obtained by cutting the tree at six clusters (chosen by comparing the sum of squared error between each data set and the mean of all data sets within the cluster containing that data set, which showed a leveling off after six clusters; Extended Data Fig. 2a).

### Quantification of eCLIP signal at multi-copy and other repetitive elements

A separate pipeline was developed to quantify enrichment for retrotransposable and other multi-copy elements. A database of multicopy elements was generated, including 5,606 transcripts obtained from GENCODE v19 covering 34 abundant non-coding RNAs including rRNA, snRNA, and vault RNAs as well as their pseudogenes, 606 tRNA transcripts obtained from GtRNAdb (including versions with both genome flanking sequences and including the canonical CCA tail)^38^, 705 human repetitive elements obtained from the RepBase database (v. 18.05)^39^, 501 60mer sequences containing simple repeats of all 1 to 6-nt *k*mers, and the rRNA precursor transcript NR_046235.1 obtained from GenBank. Each transcript was assigned to one of 185 families of multi-copy elements (for example, RNA18S, Alu, antisense Alu, simple repeat, and so on). Within each family, transcripts were given a priority value, with primary transcripts prioritized over pseudogenes.

Post-adaptor trimming paired-end sequencing reads were mapped to this repetitive element database using bowtie2 (v. 2.2.6) with options ‘-q–sensitive -a -p 3–no-mixed –reorder’ to output all mappings. Read mappings were then processed according to the following rules. First, for each read pair only mappings with the lowest mismatch score (fewest mismatches and insertions or deletions) were kept. Next, for equally scoring mappings within a repeat family described above, the mapping to the transcript with the highest priority was identified as the ‘primary’ match. Only read pairs that mapped to a single repeat family were considered, whereas read pairs that mapped with equal scores to multiple repeat families were discarded from quantification at this stage. Mapping to the reverse strand of a transcript was considered distinct from forward strand mapping, such that each family paired with a separate antisense family composed of the same transcripts with the same priority order (except for simple repeats, which were all combined into one family).

Next, repeat mappings were integrated with unique genomic mappings identified from the standard eCLIP processing pipeline (described above) as follows. If a read pair mapped both uniquely to the genome and to a repetitive element, the mapping scores were compared; if the unique genome mapping was more than two mismatches per read (24 alignment score for the read pair) better than to the repeat element, the unique genomic mapping was used; otherwise, it was discarded and only the repeat mapping was kept. Next, PCR duplicate removal was performed (similar to the standard eCLIP processing pipeline) by comparing all read pairs based on their mapping start and stop position (either within the genome or within the mapped primary repeat) and unique molecular identifier sequence, removing all but one read pair for read pairs that shared these three values. Finally, the number of post PCR-duplicate removal read pairs mapping to each multi-copy family was counted in both IP and paired input sample and normalized for sequencing depth (counting post-PCR duplicate read pairs from both unique genomic mapping and repeat mapping). In addition, to better quantify signal to RepBase elements, RepeatMasker-identified repetitive elements in the hg19 genome were obtained from the UCSC Genome Browser. Element counts for RepBase elements were determined as the sum of repeat family-mapped read pairs plus uniquely genome-mapped read pairs that overlapped RepeatMasked RepBase elements. After removing repeat-mapping elements, the remaining reads were grouped and quantified on the basis of transcript region annotations (CDS, 3′UTR, 5′UTR, proximal or distal intronic, non-coding exonic, intergenic, or antisense to GENCODE transcripts). Significance was determined by Fisher’s exact test, or Pearson’s *χ*^2^ test where appropriate.

To summarize overall eCLIP signal, a relative information content metric was applied. The relative information content of each element in each replicate was calculated as *p*_*i*_ × log_2_(*p*_*i*_/*q*_*i*_), where *p*_*i*_ and *q*_*i*_ are the fraction of total reads in IP and input, respectively, that map to element *i*. A merged relative information for both replicates was calculated by defining *p*_*i*_ as the average fraction of total reads between the two biological replicates. To cluster data sets, dimensionality reduction was performed on element-relative information from the combination of both replicates using the *t*-SNE algorithm in MATLAB (2018a) with correlation distance, ‘exact’ algorithm, and perplexity = 10. To identify clusters, clustering was performed in using the DBSCAN (v1.0) MATLAB package, with options epsilon = 3 and MinPts = 2.

### Quantification of eCLIP signal at region level

For analyses that used binding considered at the level of regions (for example, 3′UTR, CDS, or proximal intronic), read density was counted for the indicated region for both IP and paired input, and significance was determined by Fisher’s exact test (or Yates’s *χ*^2^ test if all observed and expected values were above 5). Only regions with at least 10 reads in one of IP or input, and where at least 10 reads would be expected in the comparison data set given the total number of usable reads, were considered, and significant regions were defined as those with fold enrichment ≥4 and *P* ≤ 0.00001.

### KD–RNA-seq

#### Experimental methods

Individual RBPs were depleted from HepG2 or K562 cells by either RNA interference (RNAi) or CRISPR-mediated gene disruption. RNAi was performed by transducing cells with lentiviruses expressing shRNAs (TRC collection) targeting an RBP followed by puromycin selection for 5 days. CRISPR-mediated gene disruption was performed by transfecting cells with a plasmid expressing Cas9 and a guide RNA (gRNA) targeting an RBP, followed by puromycin selection for 5 days. In each case, knockdowns were performed in biological duplicate along with a pair of control knockdowns using a scrambled shRNA or gRNA. Protein was extracted from half of each sample and used to confirm knockdown of the target RBP by western blotting. RNA was extracted from half of each sample and used to perform qRT–PCR to confirm knockdown of the targeted RBP transcript. We strived to obtain a knockdown efficiency of the target protein and/or RNA of at least 50% compared to the scrambled control, and for the knockdown efficiency to be within 10% between replicates. We used the extracted RNA to prepare RNA-seq libraries with the Illumina Tru-seq stranded mRNA library preparation kit. Paired-end 100-bp reads were generated from the RNA-seq libraries to an average depth of 63 million reads per replicate, and a minimum of 20 million reads per replicate, on an Illumina HiSeq 2500.

### Primary data processing

Reads were aligned to both GRCh37 using the GENCODE v19 annotations and GRCh38 using the GENCODE v24 annotations using both TopHat version 2.0.8^40^ with Bowtie2 version 2.1.0^41^, and STAR version 2.4.0^42^. All analyses described in this manuscript used the GRCh37/GENCODE v19 alignments, but the GRCh38/GENCODE v24 alignments are also available at the ENCODE portal. In all cases, alignments were performed against the male reference genome sequence for HepG2 cells or the female reference genome for K562 cells and simultaneously to the ERCC spike-in sequences. The command line parameters for the TopHat alignments were: -a 8 -m 0–min-intron-length 20–max-intron-length 1000000–read-edit-dist 4–read-mismatches 4 -g 20–no-novel-juncs–no-discordant–no-mixed. In some rare cases, TopHat 2.0.8 misassigned some reads to both strands or did not assign reads to either strand. To correct these errors, we used a custom script, tophat_bam_xsA_tag_fix.pl, to properly assign the SAM flag values. Gene expression levels were quantified using RSEM (v1.2.23)^43^ and Cufflinks (v2.0.2)^44^. Only samples with a Pearson correlation coefficient on FPKM values of 0.9 or greater between replicates were used for further analysis. Samples with a correlation below 0.9 were repeated. We used the custom script (makewigglefromBAM-NH.py) to convert the single .bam alignment files into plus or minus strand and unique and multi-mapped .bam files, and then converted the intermediate .bam files into bigwig files. A single, final .bam file was generated for each RNA-seq sample by merging the .bam files that contained the aligned read with the one that contained the unmapped reads. The merged .bam and bigwig files were submitted to the ENCODE Data Coordination Center (https://www.encodeproject.org/). In total, 237 HepG2 knockdown experiments (223 shRNA and 14 CRISPR) and 235 K562 knockdown experiments (217 shRNA and 18 CRISPR) were used for further analysis.

### Gene expression quantification

Salmon (v1.1.0)^45^ was used with the –gcBias option to normalize for local GC content and quantify transcript abundance. Transcripts were then merged to genes using tximport (v1.14.2)^46^, after which CQN (v1.32.0)^47^ was used to normalize for gene-level GC content and length biases. DESeq2 (v1.26.0)^48^ was then used to quantify differential expression, with differentially expressed (DE) genes defined as those with a *P* value < 0.05 and adjusted *P* (*P*_adj_) < 0.05.

 For the purposes of simplifying the analysis, we considered significant differential expression to be strong if |log_2_(fold-change)| ≥ 2, moderate when 1< |log_2_(fold-change)| < 2, and weak when |log_2_(fold-change)| ≤ 1.

### Splicing quantification

Differential alternative splicing (AS) events were analysed using rMATS (v 3.2.1.beta)^49^. The knockdown replicate bam files and their control replicate bam files with the Gencode v19 annotation file were analysed using rMATS, to report five types of the differential AS events: SE (skipped exon), MXE (mutually exclusive exons), A3SS (alternative 3′ splice site), A5SS (alternative 5′ splice site) and RI (retained intron). Events with |inclusion level difference| > 0.05, *P* < 0.05 and FDR < 0.05 were identified as significantly differentially expressed AS events.

MISO (mixture of isoforms; v misopy-0.5.2)^50^ was used to detect differentially processed tandem 3′ UTR events (alternatively poly(A) site usage). Four pairwise comparisons between the two knockdown samples and two controls were run using compare-miso: KD-rep1 versus CN-rep1, KD-rep1 versus CN-rep2, KD-rep2 versus CN-rep1 and KD-rep2 versus CN-rep2. Significant tandem 3′ UTR events were identified if abs(Bayes factor) ≥5 and *P* < 0.05 on both the more_reads(KD-rep1, KD-rep2) versus fewer_reads(CN-rep1, CN-rep2) comparison and the fewer_reads(KD-rep1, KD-rep2) versus more_reads(CN-rep1, CN-rep2) comparison.

For the purposes of simplifying the analysis, we considered significant differential alternative splicing levels to be strong if |Δ*Ψ*| ≥ 30%, moderate when 15% ≤ |Δ*Ψ*| < 30%, and weak if 5% < |Δ*Ψ*| < 15%.

### Batch normalization of RBP KD–RNA-seq data

Batch effects are common in large data sets and must be corrected and accounted for^51^. To correct for batch effects, for each batch of experiments performed on a given day, the same scrambled shRNA or gRNA was used as a non-specific control alongside a batch of experimental shRNAs or gRNAs that targeted a set of RBPs. This provided a consistent, non-specific control experiment in every batch that could be used to normalize data downstream. In addition to biological controls, if a given batch of biological samples was too large to make all the RNA-seq libraries in parallel, libraries were made from the non-specific control RNA samples in each subset of libraries made from a given biological batch. Analyses that compared eCLIP peaks with gene expression or alternative splicing changes in RNA-seq upon RBP knockdown used changes identified relative to these within-batch paired controls. However, to enable further integrated analyses, additional batch correction was performed (Supplementary Text, Supplementary Fig. 7).

### RNA Bind-N-Seq

#### Experimental methods

RBNS experiments were performed as indicated in the protocol included on each experiment at the ENCODE portal. In brief, randomized RNA oligonucleotides (20 or 40 nt) flanked by constant adaptor sequences were synthesized and incubated with an SBP-tagged recombinant RBP (consisting minimally of all annotated RNA-binding domains) at several concentrations (typically five, ranging from 5 to 1,300 nM). RNA–protein complexes were isolated with streptavidin-conjugated affinity resin and eluted RNA was prepared for deep sequencing, resulting in 10–20 million reads per RBP pulldown concentration with a similar number of input reads sequenced per in vitro transcription reaction.

### Data processing

RBNS *k*mer enrichments (*R* values) were calculated as the frequency of each *k*mer in the pulldown library reads divided by its frequency in the input library; enrichments from the pulldown library with the highest individual *k*mer *R* value were used for each RBP. The mean and s.d. of *R* values were calculated across all *k*mers for a given *k* to calculate the RBNS *Z*-score for each *k*mer. 

RBNS motif logos were made using the following iterative procedure for *k* = 5: the most enriched 5mer was given a weight equal to its excess enrichment over the input library (= *R *– 1), and all occurrences of that 5mer were masked in both the pulldown and input libraries to eliminate subsequent counting of lower-affinity ‘shadow’ 5mers (for example, GGGGA, shifted by 1 from GGGGG). All enrichments were then recalculated on the masked read sets to obtain the most enriched 5mer and its corresponding weight, with this process continuing until the enrichment *Z*-score (calculated from the original *R* values) was less than 3. All 5mers determined from this procedure were aligned to minimize mismatches to the most enriched 5mer, with a new motif initiated if the number of mismatches plus offsets exceeded two. The frequencies of each nucleotide in the position weight matrix, as well as the overall percentage of each motif, were determined from the weights of the individual aligned 5mers that went into that motif; empty unaligned positions before or after each aligned 5mer were assigned pseudocounts of 25% of each nucleotide, and outermost positions of the motif logo were trimmed if they had >75% unaligned positions. To improve the robustness of the motif logos, the pulldown and input read sets were each divided in half and the above procedure was performed independently on each half; only 5mers identified in corresponding motif logos from both halves were included in the alignments to make the final motif logo. In Fig. 3a, only the top RBNS motif logo is shown if there were multiple logos (all motifs displayed on the ENCODE portal within the ‘Documents’ box for each experiment).

### Immunofluorescence, microscopy imaging and data processing

HepG2 cells were seeded in poly-l-lysine-coated 96-well clear bottom plates (Corning; plate number 3882 half-area microplates), at a concentration of 2,000 cells per well in DMEM + 10% FBS. After 72 h in standard growth conditions (37 °C and 5% CO_2_), cells were fixed with 3.7% formaldehyde, permeabilized in PBS + 0.5% Triton X-100 and blocked in PBS + 0.2% Tween-20 + 2% BSA (PBTB), all conducted for 20 min at room temperature. Primary antibodies directed against specific RBPs (all rabbit antibodies) and marker proteins were subsequently applied to the cells at a final concentration of 2 μg/ml in PBTB and incubated overnight at 4 °C. The cells were next washed three times for 10 min each in PBST and incubated with secondary antibodies (Alexa647 donkey anti-rabbit and Alexa488 donkey anti-mouse, both diluted 1:500 in PBTB) for 90 min at room temperature. After three PBTB washes, the cells were counterstained with DAPI for 5 min, washed three times in PBS and stored in PBS at 4 °C. Subcellular marker antibodies and dilutions used were as follows: rat anti-α-tubulin, MCA78G, 1:200 (Serotec, Bio-Rad); mouse anti-CD63, ab8219, 1:200 (Abcam); mouse anti-coilin, GTX11822, 1:100 (GeneTex); mouse anti-DCP1a, sc100706, 1:200 (Santa Cruz Biotechnology); mouse anti-fibrillarin, ab4566, 1:200 dilution (Abcam); mouse anti-GM130, #610822, 1:200 (Becton Dickinson); mouse anti-KDEL, ENZSPA827D, 1:200 (Enzo Life Sciences); mouse anti-phosphotyrosine, #9411S, 1:200 (NEB); mouse anti-PML, sc-966, 1:50 (Santa Cruz Biotechnology); mouse anti-SC35, GTX11826, 1:200 (GeneTex). For staining with Mitotracker (Molecular Probes, M22426), cells were incubated with 100 nM dye in tissue culture medium for 45 min at 37 °C before fixation. For staining with phalloidin (Sigma, P5282), cells were incubated with 50 μg/ml of phalloidin for 20 min before DAPI staining.

Imaging was conducted on an ImageXpress Micro high content screening system (Molecular Devices). For each RBP–marker combination, 10–20 high-resolution images were acquired in the DAPI, FITC and Cy5 channels, using a 40× objective. Automated laser-based auto-focusing and auto-exposure functions were used for sample imaging, with exposure times ranging from 250 to 3,000 ms, 100 to 500 ms and 50 to 100 ms for RBP, marker and DAPI channels, respectively. Raw unprocessed greyscale images from individual channels were acquired as high-resolution TIF files of 726 kb each. An in-house MATLAB script was developed to batch normalize image intensity values and add blue, green or red colours to the respective channels, which were subsequently merged as colour JPEG files. The final images were uploaded on a server accessible through the RBP Image Database website. A MySQL relational database (version 5.1.73) was implemented, along with a MyISAM storage engine, to store the images, data annotations and characteristics. A controlled vocabulary of descriptors was devised to document RBP subcellular localization features.

Image analysis to quantify nuclear:cytoplasmic staining ratios, or to assess the degree of RBP targeting to punctate subcellular structures (for example, Cajal bodies, nuclear speckles, nuceloli, Golgi and P-bodies), was conducted using ‘Granularity’, ‘Colocalization’ and ‘Multi Wavelength Cell Scoring’ analysis modules from the MetaXpress v3.1 software (Molecular Devices), according to the manufacturer’s recommendations. For localization categories including microtubules, actin, cell cortex, ER, focal adhesions, mitochondria and mitotic apparatus, manual localization grading was conducted by ranking candidate RBPS as strongly or weakly co-localized with respective protein markers. The Circos plot of localization co-occurrance (Extended Data Fig. 10a) was generated by drawing one line between every pair of categories for each RBP that shared both localization annotations. Nuclear annotations are indicated in purple, cytoplasmic in red, and lines between nuclear and cytoplasmic annotations are indicated in yellow.

### ChIP–seq

#### Experimental methods

Chromatin IP was implemented according to the ChIP Protocol optimized for RNA-binding proteins (https://www.encodeproject.org/documents/e8a2fef1-580b-45ad-b29c-fffc3d527202/@@download/attachment/ChIP-seq_Protocol_for_RNA-Binding_Proteins_ENCODE_Fu_lab_RuiXiao.pdf). In brief, before coupling with RBP antibodies, magnetic beads were equilibrated by washing with ChIP dilution buffer and blocked with glycogen, BSA and tRNA in ChIP dilution buffer. Between ten million and twenty million HepG2 and K562 cells were crosslinked in 1% formaldehyde diluted in 1× PBS for 20 min and then quenched by adding glycine. Cell nuclei were extracted by resuspending the cell pellet with cell lysis buffer with occasional inversion. Nucleus pellets resuspended in nuclear lysis buffer were sonicated with a Branson Sonifier cell disruptor. Ninety-five per cent of nuclear lysate was diluted to a final concentration of 1% triton X-100, 0.1% sodium deoxycholate and 1× proteinase inhibitor cocktail and was subjected to IP with antibody-coupled beads; the other 5% of nuclear lysate was used as input chromatin. Stringent washes were performed before elution. Input and immunoprecipitated chromatin DNAs were recovered by decrosslinking, RNase A digestion, proteinase K treatment, phenol/chloroform extraction and precipitation with ethanol. Library construction was performed using the ChIP–seq Sample Prep Kit (Illumina). DNA libraries between 200 and 400 bp were gel-purified, quantified with Qubit and sequenced on the Illumina HiSeq 2000/2500. All RBP ChIP–seq experiments were performed in duplicate. Antibodies used in RBP ChIP–seq experiments were validated by IP and shRNA or CRISPR knockdown according to ENCODE RBP antibody characterization guidelines.

### Data processing

RBP ChIP–seq data sets used in this study were processed by the ENCODE Data Coordinating Center with the same uniform processing pipelines described previously for transcription factor ChIP–seq (https://www.encodeproject.org/chip-seq/transcription_factor/). After removal of low-quality and PCR duplicate reads, peaks were identified with SPP and reproducible peaks across biological replicates were identified with the IDR pipeline to yield two sets (optimal and conservative) of peaks at an IDR threshold of 0.05^52^.

### Integrated analysis

#### Saturation analysis

Saturation analysis of eCLIP and KD–RNA-seq data was performed by randomly shuffling the order of data sets 100 times, subsampling 1 through all data sets, and calculating the desired metrics. Gene level saturation analysis of RBP binding was calculated first by taking all unique genes that were bound by an IDR filtered peak in an eCLIP experiment. Then, each eCLIP experiment was iteratively added to the previous experiment, counting only unique genes in any experiment. Saturation analysis of differentially expressed genes from KD–RNA-seq was performed similarly, based on differentially expressed genes identified with DESeq2. Genes were identified as differentially expressed if they had a *P*_adj_ of <0.05 between knockdown and control. Alternative versions of this analysis used all genes (Extended Data Fig. 2g), only genes with TPM >1 in HepG2 and K562 cells (Supplementary Fig. 13a), or only genes with TPM >1 in either HepG2 or K562 cells (Supplementary Fig. 13b), using average gene-level expression from two rRNA-depleted RNA-seq experiments in HepG2 (ENCODE accession ENCFF533XPJ, ENCFF321JIT) and K562 cells (ENCFF286GLL, ENCFF986DBN). The set of differentially expressed and bound genes was determined by taking all genes that were differentially expressed upon RBP KD that contained at least one IDR-filtered peak in the corresponding eCLIP experiment in the same cell type.

Differentially spliced events were defined as those with *P* < 0.05, FDR < 0.1, and change in per cent spliced in (|Δ*Ψ*|) > 0.05 from rMATS analysis (described above). The number of unique events was defined as the number of non-overlapping events obtained upon combining all experiments for a given sampling. A differentially spliced event was considered bound if for any RBP in which the event was differentially included upon KD, there was an eCLIP peak for the same RBP in the same cell type between the start of the upstream flanking exon and the end of the downstream flanking exon for skipped exons and mutually exclusive exons, the start of the upstream flanking exon and end of the common exon region for A3SS, the start of the common exon and end of the common exon region for A5SS, and the start of the upstream and stop of the downstream exons for retained introns.

To perform saturation of transcript regions, the highest-expressed transcript for each gene was first identified using transcript-level quantification from the same rRNA-depleted RNA-seq experiments described above. The following regions were then identified: the entire unspliced transcript (pre-mRNA), all exons (exon), 5′ UTR, CDS, 3′UTR, all introns (intron), 100-nt intronic regions flanking the 5′ and 3′ splice sites (splice site), proximal intronic regions extending from 100 nt to 500 nt from the 5′ and 3′ splice site (proximal intron), and distal intronic regions extending from 500 nt and beyond from the 5′ and 3′ splice sites. Saturation calculations were then performed as described above for all genes (Supplementary Fig. 13c, e–g) or only genes with TPM > 1 in both K562 and HepG2 cells (Extended Data Fig. 2i, Supplementary Fig. 13d), and plotted as either the total number of bases covered (Supplementary Fig. 13c, d), or the fraction of covered bases divided by the total number of bases in that annotation across all genes (Extended Data Fig. 2i).

The fold-increase in bases covered was calculated by dividing the number of bases covered in a subsampling of *n* + 1 data sets divided by the number covered in subsampling *n* data sets. Analysis of the fold-increase between one and two data sets (Supplementary Fig. 13f) was determined by first taking all 73 RBPs profiled in both HepG2 and K562 cells, and calculating the fold-increase in covered bases by considering 146 comparisons including HepG2 followed by K562 and K562 followed by HepG2. Then, for each of the 146 comparisons, 10 other random data sets were chosen from the same cell type, and for each of the 10, the fold-increase in covered bases from adding that data set to the first was calculated.

To compare the fold-increase between profiling new RBPs in additional cell lines (Supplementary Fig. 13g), eCLIP data sets profiling RBFOX2, IGF2BP1, IGF2BP2, and IGF2BP3 in H9 human embryonic stem cells were obtained from the Gene Expression Omnibus (GSE78509)^53^, and added as the 224th data set. These were compared against profiling a new RBP in K562 or HepG2 cells (calculated by adding each of the 150 profiled RBPs as the 222nd (if it was profiled in both cell types) or 223rd (if it was profiled in only one cell type) data sets for other RBPs), or a profiled RBP done in second cell type (calculated by sampling 222 data sets and adding the 223rd).

### Preservation of RBP regulation across cell types

To consider binding across cell types, first the highest-expressed transcript for each gene was identified using transcript-level quantification from the same rRNA-depleted RNA-seq experiments described above and used as representative for that gene. Next, genes were categorized on the basis of the relative expression difference between K562 and HepG2 cells: unchanged (fold-difference ≤ 1.2), weakly (1.2 < fold-difference ≤ 2), moderately (2 < fold-difference ≤ 5) or strongly (fold-difference > 5) differential (each of which required expression TPM ≥ 1 in both K562 and HepG2 cells), cell type-specific genes (TPM < 0.1 in one cell type and TPM ≥ 1 in the other), or other (containing all other genes in GENCODE v19). Peaks were then categorized on the basis of the expression change of their associated gene (Supplementary Fig. 13h).

Analysis of preservation of binding across cell types was considered in three ways. First, for each peak identified in one cell type, the fold enrichment for that region in the other cell type was calculated and considered for each gene type (Fig. 2d). For further analyses, two groups of peaks were then identified: those that were ≥4-fold enriched in the other cell type, and those that were not enriched in the other cell type. The fraction of peaks associated with a gene class that were either ≥4-fold or not enriched were then considered for each gene class separately (Fig. 2e). Second, the set of peaks that were ≥4-fold enriched (and the set not enriched) was compiled across all genes, and the fraction associated with each gene class were then reported (Extended Data Fig. 2k). Finally, peak overlap between cell types (Extended Data Fig. 2j) was calculated by determining the fraction of IDR peaks identified in one cell type that overlap (requiring at least 1 nt overlap) IDR peaks identified in the second cell type. For all comparisons, significance between groups was determined by two-sided Kolmogorov–Smirnov test.

### Motif comparisons between RBNS and eCLIP

eCLIP 5mer and 6mer *Z*-scores (in Fig. 3b and elsewhere) were calculated as previously described^54^. In brief, peaks and a shuffled background set of peaks that preserved the region of binding (3′UTR, 5′UTR, CDS, exon, proximal and distal intron) were generated. EMBOSS compseq^55^ was used on these two peak sets and the *Z*-scores of the difference between real and background 5mer and 6mer frequencies were calculated.

To produce eCLIP logos in a similar manner for comparison with RBNS logos, an analogous procedure was carried out on the eCLIP peak sequences (for this analysis, eCLIP peaks with at least twofold enrichment were used): the two halves of the RBNS pulldown read set were replaced with the two eCLIP replicate peak sequence sets (each peak was extended 50 nt upstream of its 5′ end, as some RBPs have motif enrichments symmetrically around or only upstream of the peak starts), and the input RBNS sequences were replaced by random regions within the same gene as each peak that preserved peak length and transcript region (5′ and 3′ UTR peaks were chosen randomly within that region; intronic and CDS peaks were shuffled to a position within the same gene that preserved the peak start’s distance to the closest intron–exon boundary to match sequence biases resulting from CDS and splicing constraints). The enrichment *Z*-score threshold for 5mers included in eCLIP logos was 2.8, as this threshold produced eCLIP logos containing the most similar number of 5mers to that of the *Z* ≥ 3 5mer RBNS logos. Each eCLIP motif logo was filtered to include only 5mers that occurred in both of the corresponding eCLIP replicate logos. eCLIP motif logos were made separately for all eCLIP peaks, only 3′UTR peaks, only CDS peaks, and only intronic peaks, with the eCLIP logo of those 4 (or 8 if CLIP was performed in both cell types) with the highest similarity score to the RBNS logo shown in Fig. 3a, where the similarity score was the same as previously described to cluster RBNS logos (eCLIP logos for all transcript regions shown in Extended Data Fig. 3e). To determine the significance of overlap between RBNS and eCLIP, a hypergeometric test was performed with 5mers in all RBNS logos, eCLIP logo 5mers (for peaks in the region with highest similarity score to the RBNS logo), and 5mers in their intersection, relative to the background of all 1,024 5mers; overlap was deemed significant if *P* < 0.05. The top ‘eCLIP-only’ logo in each region was the highest eCLIP logo, if any, comprised of 5mers that had no overlap with any RBNS *Z* ≥ 3 5mers (always using at least the top ten RBNS 5mers if there were fewer than 10 with *Z* ≥ 3).

All eCLIP/RBNS comparisons were for the same RBP with the following exceptions in which the eCLIP RBP was compared to a closely related RBNS protein: KHDRBS2 eCLIP versus KHDRBS1 RBNS; PABPN1 eCLIP versus PABPN1L RBNS; PTBP1 eCLIP versus PTBP3 RBNS; PUM2 eCLIP versus PUM1 RBNS; and RBM15 eCLIP versus RBM15B RBNS.

### Splicing regulatory effects of RBNS+ and RBNS– eCLIP peaks

To assess the splicing regulatory effects of RBNS+ and RBNS– eCLIP peaks for Fig. 3c, only rMATS skipped exons with a *Ψ* between 0.05 and 0.95 in at least one of the controls or KDs were considered for each RBP. Each eCLIP peak (extended 50 nt 5′ of the peak start) was first checked for whether it overlapped the SE, and if not then for whether it overlapped the upstream or downstream flanking 250 nt. To compare the magnitude of splicing changes upon KD for eCLIP+ versus eCLIP– skipped exons while minimizing the confounding factors of different wild-type host gene expression level and skipped exon *Ψ* values among these two sets of skipped exons, we created a matched set of eCLIP– skipped exons by selecting for each eCLIP+ skipped exon a skipped exon in the same decile of wild-type gene expression and wild-type *Ψ* for each corresponding skipped exon with an eCLIP peak. A cumulative distribution function of the Δ*Ψ* changes upon KD was compared for the eCLIP+ versus eCLIP– skipped exons in each of the six skipped exon (SE) direction–eCLIP region combinations ([included, excluded SE] × [peak over SE, upstream intron, downstream intron]), with significance *P* < 0.05 for a one-sided Wilcoxon rank-sum test that |Δ*Ψ*|_SE, peak_ > |Δ*Ψ*|_SE, no peak_. If the eCLIP+ versus eCLIP– comparison was significant, the eCLIP peaks were divided into those that did and did not contain the top RBNS 5mer. The Δ*Ψ* values for all RBPs in each of the six skipped exon direction–eCLIP regions were combined for comparison in Fig. 3c; see Extended Data Figure 4c for RBPs that were significant in each region (12 included and 4 excluded upon KD, upstream intron eCLIP peak; 11 included and 2 excluded upon KD, skipped exon eCLIP peak; 7 included and 7 excluded upon KD, downstream intron eCLIP peak). To assess eCLIP peaks with or without the top ‘eCLIP-only’ *k*mer, the top 5mer from the aforementioned ‘eCLIP-only’logo was used from the first region with an eCLIP-only logo among: all peaks; CDS peaks; intron peaks; and 3′UTR peaks (the more highly enriched 5mer if eCLIP was performed in both cell types). The resulting ‘eCLIP-only’ 5mers for Extended Data Fig. 4d were: CELF1 (CUCUC), EIF4G2 (GUGUG), EWSR1 (CGCGG); FUBP3 (UUGUU); FUS (GUGUG); HNRNPC (GUCGC); HNRNPK (UCCCC); HNRNPL (none); IGF2BP1 (GUGUG); IGF2BP2 (CGCCG); KHDRBS2: (none); KHSRP (none); PABPN1L (CGCGG); PCBP2 (CGGCG); PTBP3 (GAAGA); PUM2 (UUUUU); RBFOX2 (GGGGG); RBM22 (GGUAA); SFPQ (UCCGG); SRSF5 (CGGCG); SRSF9 (CUGGA); TAF15 (AGGGA); TARDBP (GAAGA); TIA1 (CGCCG); TRA2A (GAGGG).

### Overlaps between RBP binding and gene expression perturbation upon KD–RNA-seq

To increase sensitivity for gene expression analysis, significant binding was determined at the level of transcript regions (including 5′UTR, CDS, 3′UTR, and introns) instead of using peaks. To identify significant enrichment between binding and expression changes, genes with significantly enriched eCLIP signal at regions (*P* ≤ 0.00001 and log_2_(fold enrichment) ≥ 4, as described above) were overlapped with the set of genes with significantly altered expression in KD–RNA-seq (*P*_adj_ < 0.05 between knockdown and control from DEseq analysis). Enrichment was calculated separately for knockdown-increased and knockdown-decreased genes, with significance determined by Fisher’s exact test (or Yates’s *χ*^2^ test if all observed and expected values were above 5). Comparisons with either knockdown-increased or knockdown-decreased genes from KD–RNA-seq were performed only if more than 10 genes showed significant changes. To avoid biases due to RNA abundance, for each comparison of a region type with each eCLIP data set, a background set of genes was created by identifying all genes for which the region type (5′UTR, CDS, 3′UTR) had at least 10 reads in one of IP or input; at least 10 reads would be expected in the opposite (IP or input) data set given the total number of usable reads. For cumulative distribution plots, genes were separated on the basis of their eCLIP fold enrichment in IP versus input for the indicated transcript region.

### RBP binding correlation with knockdown-perturbed splicing (splicing maps)

RBP binding or splicing maps were generated using eCLIP-normalized (reads per million) read densities overlapped with alternatively spliced (AS) regions from rMATS JunctionCountsOnly files from the same cell type using the RBP-Maps methodology^24^ (Supplementary Text, Supplementary Fig. 14). Analyses described used only events with rMATS *P* < 0.05, FDR < 0.1, and |Δ*Ψ*| > 0.05 in knockdown versus control RNA-seq.

Correlation between splicing maps was defined as the Pearson correlation (*R*) between a vector that contained both included-upon knockdown and excluded-upon knockdown RBP-responsive event eCLIP enrichment for each RBP. If an RBP had fewer than the minimum required number of events (100 for skipped exons or 50 for alternative 5′ or 3′ splice site events) for either knockdown-included or knockdown-excluded events, the correlation was calculated only using the other event type.

To generate cross-RBP splicing maps, the above approach was modified by taking the set of differentially included (or excluded) skipped exons identified in knockdown of RBP *A* and calculating the eCLIP splicing map separately for every other RBP within the same binding class (determined in Fig. 2a) as RBP *A*, including the normalization against a background of eCLIP signal for native skipped exon events (as shown for HNRNPC knockdown-included, RBFOX2 knockdown-excluded, and TIA1 knockdown-included skipped exons in Extended Data Fig. 8b, Fig. 5d, and Extended Data Fig. 8e, respectively). The average across all RBPs was then used to calculate the average cross-RBP enrichment (Extended Data Fig. 8a).

To calculate the number of RBPs bound per exon, the set of spliceosomal RBPs was taken from manual annotation of RBP functions (described above and listed in Supplementary Data 1). The number of reproducible (IDR) peaks at each position relative to splice sites was summed across all RBPs and divided by the total number of skipped or constitutive exons.

### Comparison of DNA- and RNA-binding properties of RBPs

For integrative analyses, DNaseI HS data (http://genome.ucsc.edu/cgi-bin/hgFileUi?db=hg19&g=wgEncodeOpenChromSynth), histone modifications by ChIP–seq from ENCODE or the Broad Institute (http://genome.ucsc.edu/cgi-bin/hgFileUi?db=hg19&g=wgEncodeBroadHistone) and eCLIP–seq data from ENCODE (https://www.encodeproject.org) were downloaded and compared with RBP ChIP–seq data.

To explore the possibility that some RBP chromatin association events might be coupled with their direct RNA-binding activities in cells, RNA binding peaks were compared with DNA binding signals as assayed by ChIP–seq to quantify enrichment. Only eCLIP peaks in gene body regions (excluding promoter and terminator regions, defined as the 1 kb surrounding regions of TSS and TTS) were considered. ChIP–seq signals were calculated for each eCLIP peak along with surrounding regions that are ten times the length of eCLIP peak on each side. Wilcoxon rank-sum tests were then performed to see whether ChIP–seq signals were enriched at the middle regions relative to the flanking regions.

To see whether those differentially expressed genes after RBP knockdown were enriched in RBP binding at chromatin level, equal numbers of genes with similar expression level either with or without binding to the TSS region were randomly sampled, the number of differentially expressed genes after knockdown of the RBP were counted (fold-change >1.5 or <2/3, *P*_adj_ < 0.05 by DESeq2), and one-tailed Fisher’s exact tests were then performed to test the dependence of RBP binding and differential expression. Odds ratio was defined as (*a*/*b*)/(*c*/*d*), where *a* is the number of genes with RBP ChIP–seq peaks and differential expression (or splicing) upon RBP knockdown, *b* is the number of genes with RBP ChIP–seq peaks but no differential expression, *c* is the number of genes without ChIP–seq peaks but with differential expression, and *d* is the number of genes without ChIP–seq peaks or differential expression. The above procedure was performed 100 times to give the distribution of the odds ratio, and a significant dependence was defined as when the null hypothesis was rejected at level of 0.05 at least 95 times. The correlation between RBP association and genes with regulated alternative splicing events (A3SS, A5SS, RI, MXE and skipped exon events) was investigated similarly.

### Analysis of RBP regulatory features in subcellular space

Localization annotations and calculation of nuclear versus cytoplasmic ratio were generated from immunofluorescence imaging as described above. ‘Nuclear RBPs’ were defined as those with a nuclear:cytoplasmic ratio ≥ 2, and ‘cytoplasmic RBPs’ were defined as those with a nuclear:cytoplasmic ratio ≤ 0.5. Spliced reads were defined as reads that mapped across an annotated GENCODE v19 splice junction (extending at least ten bases into each exon) and unspliced reads were defined as reads that overlapped an exon–intron junction (extending at least ten bases into both the exon and intron regions). Significance between groups was determined by Wilcoxon rank-sum test. Prediction of RNA secondary structure was performed using the RNAfold webserver (http://rna.tbi.univie.ac.at//cgi-bin/RNAWebSuite/RNAfold.cgi)^56^ with default parameters. Shown is the MFE secondary structure prediction.

### Reporting summary

Further information on research design is available in the Nature Research Reporting Summary linked to this paper.

## Online content

Any methods, additional references, Nature Research reporting summaries, source data, extended data, supplementary information, acknowledgements, peer review information; details of author contributions and competing interests; and statements of data and code availability are available at 10.1038/s41586-020-2077-3.

## Supplementary information

Supplementary Information This file contains Supplementary Methods, a Supplementary Discussion and 13 Supplementary Figures that provide further details of the analysis of RBP targets.

Reporting Summary

Supplementary Data 1 **Data summary and manual annotation of RBP functions**. For each RBP profiled by at least one assay in this study, table includes experiments performed, RNA localization observed, presence of annotated RNA binding domains, and manual literature-based annotation of RBP function.

Supplementary Data 2 **ENCODE accession identifiers of datasets used**. Tabs contain accession identifiers (for the ENCODE Data Coordination Center, https://www.encodeproject.org/) for all datasets generated in this study.

Supplementary Data 3 **RBP gene expression in ENCODE cell lines and tissues**. Contains RNA expression (in transcripts per million reads) for all RBPs profiled in this study for K562 cells, HepG2 cells, and 40 human tissues profiled by the GTEx consortium.

Supplementary Data 4 **Summary information for eCLIP experiments**. Contains summary information for all eCLIP experiments, including antibodies (accession identifiers, catalog, and lot numbers) as well as general sequencing library information (number of input reads, PCR duplication rate, and number of significantly enriched peaks).

Supplementary Data 5 **Summary information for RNA-seq experiments**. Contains summary information for RBP knockdown/RNA-seq experiments, including number of differentially expressed genes and alternative splicing events.

Supplementary Data 6 **Summary information for RBNS experiments**. Contains summary information for RNA Bind-N-Seq (RBNS) experiments, including reaction conditions (temperature and read length) as well as primary analysis results (enriched consensus motifs).

Supplementary Data 7 **Summary information for ChIP-seq experiments**. Contains summary information of ChIP-seq experiments, including number of usable reads and significantly enriched peaks, as well as experimental quality assessment metrics (PCR bottleneck coefficient, normalized and relative strand cross-correlation, and IDR reproducibility).

Supplementary Data 8 **Automated and manual quality assessment of eCLIP datasets**. Contains results from automated eCLIP quality assessment pipeline, including read number, total relative information, and IDR rescue and self-consistency ratio metrics. Tabs include 223 released datasets, 76 rejected datasets, and 50 datasets with reproducible signal of questionable reliability made available on the Gene Expression Omnibus.

Supplementary Data 9 **Summary information for questionable quality eCLIP experiments**. Contains summary information for 50 eCLIP experiments with reproducible signal of questionable reliability, including antibodies (accession identifiers, catalog, and lot numbers) as well as general sequencing library information (number of input reads, PCR duplication rate, and number of significantly enriched peaks).

Supplementary Data 10 **Summary information for eCLIP experiments failing quality assessment**. Contains summary information for 76 eCLIP experiments which failed manual quality assessment, including antibodies (accession identifiers, catalog, and lot numbers) as well as general sequencing library information (number of input reads, PCR duplication rate, and number of significantly enriched peaks).

Supplementary Data 11 **eCLIP blacklist regions**. 56 regions which showed consistent artifact signal across many eCLIP experiments and were excluded from analyses.

Supplementary Data 12 **Overlap between eCLIP and ChIP-seq peaks**. Contains statistics of overlap between eCLIP and ChIP-seq peaks.

Supplementary Data 13 **eCLIP adapters used**. Contains sequences of in-line barcoded RNA adapters used for eCLIP experiments.

## Data Availability

Raw and processed data sets are accessible using accession identifiers provided in Supplementary Data 2 or can be found using the following publication file set accession identifiers at the ENCODE Data Coordination Center (https://www.encodeproject.org): eCLIP (ENCSR456FVU), knockdown RNA-seq (HepG2: ENCSR369TWP; K562: ENCSR795JHH; secondary analysis files including DEseq, rMATS, MISO, and CUFFDIFF output: ENCSR413YAF; batch corrected gene expression and splicing analysis: ENCSR870OLK), RBNS (ENCSR876DCD), and ChIP–seq (ENCSR999WIC). In addition to the methods described below, expanded experimental and computational protocols are linked to each experiment on the ENCODE DCC (https://www.encodeproject.org). All analyses in this manuscript used the hg19 genome annotation and GENCODE v19 transcript annotations (unless otherwise noted), with hg38 processed data available at the ENCODE DCC. eCLIP data sets that did not pass quality control are available in the Gene Expression Omnibus under accession GSE107768.

## Code availability

Data processing pipeline descriptions are linked to each data set at the ENCODE Data Coordination centre (https://www.encodeproject.org) and contain links to publicly available and custom scripts. eCLIP data processing scripts are available at https://github.com/gpratt/gatk/releases/tag/2.3.2. RBNS analysis code is available at https://bitbucket.org/pfreese/rbns_pipeline/. Splicing map code is available at https://github.com/yeolab/rbp-maps. Other custom analysis scripts are available upon request.
