# Author Correction: A large-scale binding and functional map of human RNA-binding proteins

**DOI:** 10.1038/s41586-020-03067-w

**Published:** 2021-01-05

**Authors:** Eric L. Van Nostrand, Peter Freese, Gabriel A. Pratt, Xiaofeng Wang, Xintao Wei, Rui Xiao, Steven M. Blue, Jia-Yu Chen, Neal A. L. Cody, Daniel Dominguez, Sara Olson, Balaji Sundararaman, Lijun Zhan, Cassandra Bazile, Louis Philip Benoit Bouvrette, Julie Bergalet, Michael O. Duff, Keri E. Garcia, Chelsea Gelboin-Burkhart, Myles Hochman, Nicole J. Lambert, Hairi Li, Michael P. McGurk, Thai B. Nguyen, Tsultrim Palden, Ines Rabano, Shashank Sathe, Rebecca Stanton, Amanda Su, Ruth Wang, Brian A. Yee, Bing Zhou, Ashley L. Louie, Stefan Aigner, Xiang-Dong Fu, Eric Lécuyer, Christopher B. Burge, Brenton R. Graveley, Gene W. Yeo

**Affiliations:** 1 Department of Cellular and Molecular Medicine, University of California San Diego, La Jolla, CA USA; 2 Institute for Genomic Medicine, University of California San Diego, La Jolla, CA USA; 3 Program in Computational and Systems Biology, Massachusetts Institute of Technology, Cambridge, MA USA; 4 Bioinformatics and Systems Biology Graduate Program, University of California San Diego, La Jolla, CA USA; 5 Institut de Recherches Cliniques de Montréal (IRCM), Montreal, Quebec Canada; 6 Department of Genetics and Genome Sciences, Institute for Systems Genomics, UConn Health, Farmington, CT USA; 7 Medical Research Institute, Wuhan University, Wuhan, China; 8 Department of Biology, Massachusetts Institute of Technology, Cambridge, MA USA; 9 Department of Biochemistry and Molecular Medicine, Université de Montréal, Montreal, Quebec, Canada; 10 Department of Biological Engineering, Massachusetts Institute of Technology, Cambridge, MA USA; 11 Division of Experimental Medicine, McGill University, Montreal, Quebec Canada

**Keywords:** Transcriptomics, Alternative splicing

Correction to: *Nature* 10.1038/s41586-020-2077-3Published online 29 July 2020

In this Article, author Daniel Dominguez was incorrectly listed as having affiliation 7 ‘Medical Research Institute, Wuhan University, Wuhan, China’ instead of affiliation 8 ‘Department of Biology, Massachusetts Institute of Technology, Cambridge, MA, USA’. This error has been corrected online.

