# Supplementary material for: A large-scale binding and functional map of human RNA-binding proteins
Source: Nature. 2020 Jul 29;583(7818):711–9. doi: 10.1038/s41586-020-2077-3 (PMC7410833; doi:10.1038/s41586-020-2077-3)
Supplement: Supplementary file 1 — This file contains Supplementary Methods, a Supplementary Discussion and 13 Supplementary Figures that provide further details of the analysis of RBP targets. [file 41586_2020_2077_MOESM1_ESM.pdf]

nature

https://doi.org/10.1038/s41586-020-2077-3

Supplementary information

A large-scale binding and functional map of human RNA-binding proteins

In the format provided by the authors and unedited

Supplementary Information for:

A Large-Scale Binding and Functional Map of Human RNA Binding Proteins

Eric L Van Nostrand[§], Peter Freese[§], Gabriel A Pratt[§], Xiaofeng Wang[§], Xintao Wei[§], Rui Xiao[§], Steven M Blue, Jia-Yu Chen, Neal A.L. Cody, Daniel Dominguez, Sara Olson, Balaji Sundararaman, Lijun Zhan, Cassandra Bazile, Louis Philip Benoit Bouvrette, Julie Bergalet, Michael O Duff, Keri E. Garcia, Chelsea Gelboin-Burkhart, Myles Hochman, Nicole J Lambert, Hairi Li, Thai B Nguyen, Tsultrim Palden, Ines Rabano, Shashank Sathe, Rebecca Stanton, Amanda Su, Ruth Wang, Brian A. Yee, Bing Zhou, Ashley L Louie, Stefan Aigner, Xiang-Dong Fu[*], Eric Lécuyer[*], Christopher B. Burge[*] , Brenton R. Graveley[*], Gene W. Yeo[*]

[*]Correspondence and requests for materials should be addressed to Brenton Graveley (graveley@uchc.edu), Chris Burge (cburge@mit.edu), Xiang-Dong Fu (xdfu@ucsd.edu), Eric Lécuyer (eric.lecuyer@ircm.qc.ca) and Gene Yeo (geneyeo@ucsd.edu).

Supplementary Methods

RBP expression in tissues

Tissue specificity was measured as the entropy deviation from a uniform distribution among all tissues as in[1]. For each RBP, the log_2(TPM+1) was calculated for each of the 42 samples (HepG2, K562, and 40 tissues profiled by the GTEx consortium[2]), and the tissue specificity was computed as the difference between the logarithm of the total number of samples (N=42) and the Shannon entropy of the expression values for an RBP:

$$S = H_{max} - H_{obs} = \log_2(N) - \Sigma_{i=1 \ldots N}\, p_i \times log_2(p_i),$$

Where $p_i = x_i / \Sigma_{i=1 \ldots N} x_i$

for $x_i = \log_2(TPM_i + 1)$ in sample i.

The data used for the analyses were obtained from dbGaP accession number phs000424.v2.p1 in Jan. 2015. TPMs were measured using kallisto[3] on the following samples: Adipose-Subcutaneous: SRR1081567; AdrenalGland: SRR1120913; Artery-Tibial: SRR817094; Bladder: SRR1086236; Brain-Amygdala: SRR1085015; Brain-AnteriorCingulateCortex: SRR814989; Brain-CaudateBasalGanglia: SRR657731; Brain-CerebellarHemisphere: SRR1098519; Brain-Cerebellum: SRR627299; Brain-Cortex: SRR816770; Brain-FrontalCortex: SRR657777; Brain-Hippocampus: SRR614814; Brain-Hypothalamus: SRR661179; Brain-NucleusAccumben: SRR602808; Brain-SpinalCord: SRR613807; Brain-SubstantiaNigra: SRR662138; Breast-MammaryTissue: SRR1084674; Cervix: SRR1096057; Colon: SRR1091524; Esophagus: SRR1085211; FallopianTube: SRR1082520; Heart-LeftVentricle: SRR815517; Kidney-Cortex: SRR809943; Liver: SRR1090556; Lung: SRR1081283; MinorSalivaryGland: SRR1081589; Muscle-Skeletal: SRR820907; Nerve-Tibial: SRR612911; Ovary: SRR1102005; Pancreas: SRR1081259; Pituitary: SRR1077968; Prostate: SRR1099402; Skin: SRR807775; SmallIntestine: SRR1093314; Spleen: SRR1085087; Stomach: SRR814268; Testis: SRR1081449; Thyroid: SRR808886; Uterus: SRR820026; Vagina: SRR1095599.

Most RBPs showed low specificity with broad expression across tissues (Supplementary Fig. 1a), with the 10 least specific including many ubiquitously expressed basic RNA processing factors (e.g. ribosomal proteins RPS11, RPS24, and RPL23A, translation machinery factors EIF4H and EEF2, and broad regulators of RNA processing including HNRNPC and HNRNPA2B1) (Supplementary Fig. 1b, left). However, a small subset showed highly specific expression, including some with greatly increased expression in the K562 and HepG2 cancer

cell lines used for this study (including cancer-associated LIN28B, IGF2BP1, and IGF2BP3) (Supplementary Fig. 1b, right).

Identification of RBP-dependent gene expression and splicing changes

For analyses in this manuscript, each knockdown/RNA-seq dataset was compared against a non-target shRNA control infected and with library preparation performed within the same experimental batch to minimize potential batch effects in single-RBP comparisons.

Single-RBP Gene Expression Analysis

Significant gene expression changes were determined using DESeq and Cuffdiff as described in Methods, and yielded as few as 1 to as many as 5905 significantly altered genes (Supplementary Fig. 2-3, left & Supplementary Data 5). Defining the magnitude of differential expression as strong (fold-change ≥ 4), moderate (2 < fold-change < 4) and weak (fold-change ≤ 2), we observed that while the majority of changes were weak, some knockdowns lead to a thousand or more changes of at least 2-fold. On average, a similar number of up-regulated and down-regulated changes were observed, with occasional outliers (Supplementary Fig. 2-3, center). The number of genes altered did not typically correlate with knockdown efficiency (either by Western or qPCR) (Supplementary Fig. 2-3, right).

Single-RBP Alternative Splicing Analysis

Significant alternative splicing changes were determined using rMATS as described in Methods, and yielded as few as 3 to as many as 35,000 (the RNA helicase and spliceosomal protein AQR[4] in K562 cells) significantly altered alternative splicing events (Supplementary Fig. 4-5, left & Supplementary Data 5). Defining the magnitudes of differential splicing as weak (change in Percent Spliced In ($|\Delta\Psi|$) = 5% - 15%), moderate ($|\Delta\Psi|$ = 15% - 30%) or strong ($|\Delta\Psi|$ >= 30%, we observed a wide variance of the degree of altered splicing. Separating by alternative event types (SE: skipped exon, MXE: mutually exclusive exons, A5SS: alternative 5′ splice sites, A3SS: alternative 3′ splice sites, RI: retained intron, and TANDEMUTR: tandem 3′UTR), we observed that SE and TANDEMUTR events dominated the overall counts, but there was large variation with some datasets containing mostly SE and others mostly TANDEMUTR (Supplementary Fig. 4-5, center). We did not observe these results to correlate to either protein or RNA knockdown rate (Supplementary Fig. 4-5, right).

Batch correction for global analysis of knockdown/RNA-seq datasets

As alterations in gene expression or splicing patterns due to experimental batches are a common problem in large-scale genomics efforts, to enable further integrated analyses we further performed the following batch correction methods to enable cross-RBP studies of the entire knockdown/RNA-seq data resource:

Batch Correction for Gene Expression Analysis

Gene expression batch effects were reduced with ComBat[5]. The HepG2 and K562 samples were normalized separately. Genes whose expected counts were 0 on more than 80% samples of the set were filtered out prior to normalization. After ComBat normalization and Quantile Normalization, the normalized values were rounded to integers and flatted to zeros if the values were less than zero. Instead of the original control samples, two “virtual” control replicates were created by averaging the normalized expression values of all rep-1 control samples or all rep-2 control samples for each gene. Then, DESeq was used to quantitate differential expression between the normalized knockdown samples and the virtual control samples (Supplementary Fig. 6a,c). The batch normalized gene expression results are available at www.encodeproject.org (See Supplementary Data 2 for accession identifiers).

Batch Correction for Splicing Analysis

ComBat[5] was also used to reduce batch effects for alternative splicing analysis. The HepG2 and K562 samples were normalized separately. The inclusion junction counts and skipping junction counts of all samples were collected from rMATS temporary files and used to form a table with each sample in columns. “Noise” junction counts were filtered out if their values were 0 on more than 80% samples. After ComBat batch normalization and Quantile Normalization on the filtered datasets, the normalized values were rounded to integers and flatted to zeros if the values were less than zero. Next, normalized rMATS temporary files were formed using the normalized junction counts and the “noise” junction counts. Instead of the original control samples, two “virtual” control replicates were created by averaging the normalized or “noise” junction counts of all rep-1 control samples or all rep-2 control samples for each event. rMATS was then resumed on the normalized knockdown samples and the virtual control samples to detect differential alternative splicing events (Supplementary Fig. 6b,d). The batch normalized splicing results are available at www.encodeproject.org (See Supplementary Data 2 for accession identifiers).

Supplementary Discussion

Experimental quality control of eCLIP experiments

Library complexity, defined as the number of unique molecules recovered during preparation of high-throughput sequencing library, is a critical measurement of experimental quality that effects many aspects of data quality and analysis, determines whether the resulting read density is quantitative, and whether PCR biases and other artifacts will be introduced. Although library complexity is easily empirically calculated after sequencing and data processing, a quantitative metric for library complexity that can be applied prior to sequencing would enable rapid culling of poor quality experiments and could help guide a desired sequencing depth by estimating an upper bound on the number of recovered RNA fragments. We previously introduced the extrapolated CT (eCT) metric that estimates the number of PCR cycles needed to obtain sufficient material for sequencing. This metric had appealing characteristics, as it was RBP-specific, showed high correlation with PCR duplication rate, and could be directly compared against eCLIP experiments performed with IgG isotype controls or antibodies in null cell lines[6,7].

However, although the initial eCT calculation assumed an idealized 2-fold amplification rate per PCR cycle, we observed that this rate is frequently lower in practice. To properly estimate PCR efficiency during eCLIP, we noted that at our standard sequencing depths some experiments had saturated the discovery of unique fragments, which enabled us to accurately estimate the total number of pre-PCR unique fragments for these datasets. Using 6 datasets with a PCR duplication rate of greater than 90%, we observed that the best fit between the number of observed unique fragments and the estimated number of unique fragments occurred at a PCR efficiency of 1.84 (Supplementary Fig. 8a-b). We therefore defined an accurate-eCT (a-eCT) as the eCT calculated with 1.84-fold amplification per cycle instead of 2-fold.

To validate the a-eCT metric, we considered datasets that were beginning to saturate (PCR duplication rate greater than 60%). We observed that a-eCT showed strong predictive power for the number of unique RNA fragments observed ($R^2 = 0.46$, $p < 7.1 \times 10^{-38}$) (Supplementary Fig. 8c), an improvement on the prior eCT metric (MSE 0.19 versus 0.86), confirming that a-eCT provides a robust estimate of library complexity (Supplementary Fig. 8d). Thus, a-eCT enables prediction of unique fragments prior to sequencing and indicates that eCLIP of distinct RBPs can yield a range from hundreds of thousands to billions of unique fragments (Supplementary Fig. 8e).

Next, we compared a-eCT against a manual annotation of experiment quality. We observed that experiments that pass manual quality assessment had significantly lower a-eCT values than experiments that failed manual quality assessment with mean a-eCTs of 13.3 versus 14.4 respectively (Supplementary Fig. 8f, students two-sided t-test; $p < 10^{-7}$). Low a-eCT (corresponding to a highly complex library) did not always indicate high-quality eCLIP datasets, with failures due to poor reproducibility, lack of significant binding signal, and other failure modes. However, a high a-eCT value was a strong predictor of failure, typically due to a lack of the required number of unique fragments to produce reproducible peaks. To establish a maximum a-eCT threshold beyond which data are unreliable, we observed that the mean a-eCT for IgG control eCLIP experiments (which only immunoprecipitate background RNA) was 19.6. With that threshold applied, 21 out of 24 datasets with an a-eCT > 19.6 also independently failed manual QC. In all datasets examined no successful experiment had an a-eCT > 20.7, while there were still 9 experiments that did not pass manual quality control that had a higher a-eCT (Supplementary Fig. 8f).

In total, 331 out of 400 (83%) experiments had higher yield than this IgG-only value in both replicates, indicating successful immunoprecipitation of protein-bound RNA in the majority of experiments (Supplementary Fig. 8g). As we did observe a small number of high-quality datasets with a-eCT values above this cutoff (typically RBPs with high specificity for a single or small number of RNA transcripts, such as SLBP for histone RNAs), in later experiments we queried those with high a-eCT values with low-depth sequencing prior to full analysis and abandoned 36 such experiments which showed no observable binding specificity, leaving 349 datasets for analysis (Extended Data Fig. 1c).

eCLIP - data processing and peak identification

Primary data analysis of eCLIP data (including adapter trimming, read mapping, cluster identification, and input normalization) was performed as previously published[6] and is provided (including description of steps as well as commands run) as a 'Pipeline Protocol' attached to each eCLIP dataset available on the ENCODE website at https://www.encodeproject.org/documents/3b1b2762-269a-4978-902e-0e1f91615782/@@download/attachment/eCLIP_analysisSOP_v2.0.pdf) (Supplementary Fig. 9a). Briefly, sequencing reads are first demultiplexed using dual indices with standard tools provided by Illumina. Next, reads were further demultiplexed based on in-line barcodes (present in read 1) (Supplementary Data 13). At this step, a unique molecular identifier (either N_5 or N_{10}) was removed from the beginning of read 2 and saved for use at the later PCR duplicate removal

step. Next, potential adapter sequences were removed using cutadapt (v1.8.1), performed in two steps to properly remove non-full length adapter sequences we observed to drive artifact peak identification. At this step, reads with less than 18 bases were removed from further analysis. Next, we mapped reads using STAR (v2.4.0i)[8] against a database of repetitive elements (derived from RepBase (v18.05)[9] with the addition of elements including the 45S ribosomal RNA precursor), and removed reads with identified mapping (an independent method was derived to quantify mapping to repetitive elements, described in the next section). Reads were then mapped against the human genome using STAR (v2.4.0i), requiring unique mapping (all analyses described in this manuscript used mapping to GRCh37 and GENCODE v19 annotations, but mapping to GRCh38 and GENCODE v24 annotations were also deposited at the ENCODE portal). PCR duplicate reads were then identified as those with the same mapped start position and unique molecular identifier and were removed using custom scripts to obtain unique fragments. Read clusters were identified using CLIPper[10], which applies spine-fitting to identify clusters of enriched read density above local, transcript (both pre-mRNA and mRNA), and whole-genome background. Whole reads were used for this broad analysis of RBPs as the use of read 3′ ends only can cause decreased signal-to-noise for the subset of RBPs that do not crosslink in close proximity to their RNA motif[11]. However, we note that future re-analyses restricting to only read 3′ ends likely will provide increased resolution for motif and splicing regulatory map analyses for some RBPs. Finally, clusters identified in IP samples were compared against paired size-matched input to obtain significantly enriched peaks using a Fisher's Exact test (or Yates' Chi-Square test if all observed and expected values were above 5), with p-values reported not corrected for multiple hypothesis testing. An average of 6.9% of clusters were significantly enriched, although this was highly variable across the 223 datasets (Extended Data Fig. 1f). The number of significantly enriched peaks was highly correlated between replicates, indicating the capture of RBP-specific biological signal (Supplementary Fig. 9b) (Supplementary Data 4).

To identify reproducible and significantly enriched peaks across biological replicates, we used a modified Irreproducible Discovery Rate (IDR) method (Supplementary Fig. 9c). IDR requires that peaks are ranked by an appropriate metric, but we found undesirable results ranking peaks by either significance (due to the dependence on underlying expression) or fold-enrichment (due to the large variance of fold-enrichment when few reads are observed in input). Thus, we adapted relative entropy to better estimate the strength of binding in IP relative to input by defining the relative information content of a peak as $p_i \times log_2(\frac{p_i}{q_i})$, where p_i and q_i are the fraction of total reads in IP and input respectively that map to peak *i*. To confirm that this

metric captures true binding signal, we considered the RBFOX2 eCLIP dataset in HepG2. We observed 14,595 reproducible clusters when we ranked by fold enrichment, whereas 32,431 clusters were reproducible when we ranked by information content (Supplementary Fig. 9d). Given the increased number of reproducible clusters detected, we used information content to perform standard IDR analysis to identify reproducibly bound regions[12]. We then identified the set of non-overlapping peaks from both replicates that maximized information content to define a final set of reproducibly enriched peaks that corresponded to CLIPper-identified regions (Supplementary Fig. 9c). We defined reproducible and significant peaks to be the set of replicate-merged peaks that met an IDR cutoff of 0.01 as well as p-value ≤ 0.001 and fold-enrichment ≥ 8 (using the geometric mean of $\log_2$(fold-enrichment) and the least significant p-value between the two biological replicates). This method revealed that an average of 53.1% of peaks identified as significantly enriched in individual replicates were significant and reproducible, indicating high reproducibility for most experiments (Extended Data Fig. 1g). Furthermore, the number of reproducible peaks identified upon profiling the same RBP in K562 and HepG2 cells was highly correlated, providing further validation that this approach reproducibly captures RBP-specific signal (Extended Data Fig. 1h).

Automated QC Metrics to verify data quality

We next developed a set of metrics to assess the quality of ENCODE eCLIP experiments in an automated manner. We ultimately arrived at two metrics for individual replicates (a minimal unique fragment cutoff, and a “total information in peaks” cutoff) as well as a third metric to assess reproducibility across the two biological replicates (Extended Data Fig. 1d). To evaluate these metrics, we used manual quality assessment of datasets to define a reference set of high- and low-quality eCLIP datasets.

The number of unique fragments per dataset varies widely, depending on library complexity and sequencing depth (as described above). We observed that a required number of 1.5M unique fragments maximized the predictive power for datasets passing manual quality assessment (f-score = 0.79) (Supplementary Fig. 10a). Only 7 of 446 manually passed datasets do not meet this threshold: two (TBRG4, PABPC4) are not yet saturated and thus would likely pass this criteria if re-sequenced, whereas the 5 other datasets (one replicate of SLBP, two replicates of SF3B1, and SUPV3L1) are already highly saturated, but were considered high quality due to presence of signal at a small number of specific RNAs matching previous studies of these RBPs (histones, the U2 snRNP, and mitochondrial RNA respectively)[13-15]. Although the classification power of this model is low (AUC = 0.57), datasets not meeting this threshold were

more than 7-fold more likely to fail manual quality assessment (Supplementary Fig. 10b). Conversely, 30 of 222 manually failed datasets do not meet the criteria (Supplementary Fig. 10c).

Next, we considered a metric based on whether the dataset contained significant binding signal. As described above, we observed that the relative information of a peak better captures the binding information of peaks across genes with widely varying expression levels. Thus, to validate that a dataset contains significant binding information, we calculated the sum of relative information across all peaks in the dataset. We observed that this total information content score maximized the f-score of manually annotated high- and low-quality datasets at a total information content of 0.042 bits (f-score = 0.81) (Supplementary Fig. 10d). The information content model was more accurate (AUC = 0.71) (Supplementary Fig. 10e, accurately classifying 63% of ENCODE datasets with 0.36 specificity and 0.93 sensitivity (Supplementary Fig. 10f).

Next, we developed criteria to assay biological reproducibility, using two metrics based upon the Irreproducible Discovery Rate (IDR) approach that has previously been used to assay reproducibility of ChIP-seq peaks: reproducibility between real and pseudo-replicates (Rescue Ratio) and confirmation that the number of reproducible peaks between both replicates is similar (Self-Consistency Ratio)[16]. We found that cutoffs previously used for ChIP-seq data could be similarly applied to eCLIP[16], and observed that 81.9% of experiments have a passing rescue ratio of <2 (Supplementary Fig. 10g) and 71.1% of experiments have a passing self-constancy ratio of <2 (Supplementary Fig. 10h). 223 experiments pass both thresholds, while 88 are borderline (passing one of the two thresholds), and 38 fail both thresholds (Supplementary Fig. 10i). Notably, these IDR metrics have high specificity, as 151 out of 196 (77%) of experiments that meet unique fragment and total information content cutoffs and were manually judged to be high quality passed this IDR criteria. In contrast, IDR detects potential false positives by correctly failing 9 out of 56 (16%) datasets that met read depth and information content metrics, but failed manual inspection (Supplementary Fig. 10j).

Finally, we combined these metrics into one overall automated quality call requiring that each experiment passes minimum read and information content cutoffs as well as either being classified as passing or borderline based on IDR metrics (Supplementary Fig. 10k). Overall our model accurately classified 77% of eCLIP datasets with a sensitivity of 0.84 and a specificity of 0.62 (Extended Data Fig. 1e), better than any individual classification scheme.

Effect of sequencing depth on eCLIP peak identification

How deeply to sequence a CLIP-seq dataset is a major consideration (particularly at large scale), as samples must be sequenced sufficiently to robustly detect true binding signals while balancing experimental cost. To query whether the ENCODE eCLIP datasets were sequenced to sufficient depth, we considered two questions: first, how does sequencing depth affect identification of true binding sites, and second, how many reads are required to detect binding sites in any gene when accounting for variability in gene expression.

First, we asked whether peaks discovered at deeper sequencing depths were still likely to be biologically relevant. To do this we looked at RBFOX2, which is known to bind to the GCAUG motif. Overall, we observed significant enrichment for RBFOX2 binding to its motif, with 36% of RBFOX2 peaks overlapping the motif versus a mean of 6% of peaks overlapping the motif in all other datasets (Supplementary Fig. 11a). We then down-sampled the unique genomic fragments, re-called peaks, and asked how many peaks discovered at each down-sampling step overlapped the RBFOX2 motif. We observed that peaks discovered using only 10% of unique genomic fragments showed the highest motif overlap (38% on average), whereas peaks that were only discovered when going from 90% to 100% of unique genomic fragments were less likely to contain GCAUG (27% on average) (Supplementary Fig. 11b). Although this suggests that signal to noise is highest among the most abundantly covered peaks, we note that later discovered peaks were still 3.0- to 7.4-fold enriched above non-RBFOX2 datasets, indicating they still contain true binding signal (Supplementary Fig. 11b). Supporting this, we observed that conservation of later-discovered peaks was similar to those discovered earlier with a mean phastcons conservation score of 0.136 versus 0.132 (Supplementary Fig. 11c). Considering an independent dataset, PRPF8, we observed similar results when testing its known association with the 5′ splice site: although peaks discovered at low sequencing depth were less enriched for true signal, we continued to see true positive signal throughout the range of down-sampling, indicating that it is true that deeper sequencing allows for the continued discovery of high quality peaks (Supplementary Fig. 11d-e).

Second, we considered the identification of peaks as a function of transcript abundance. To explore if there was a correlation between sequencing depth and the discovery of peaks in lowly expressed genes, we calculated the correlation between gene expression and the number of reads in each peak for RBFOX2. We observed that lowly expressed genes had fewer reads per peak (as expected), whereas highly expressed genes displayed a large variation in the number of reads per peak, with only a weak correlation overall for both RBFOX2 (R^2 = 0.03) (Supplementary Fig. 11f). All other RBPs showed a similar week correlation (mean R^2 = 0.13) (Supplementary Fig. 11g). Next, we asked whether peaks at lowly expressed genes could be

detected at standard sequencing depths. Surprisingly, we found that lowly expressed genes (defined as those with TPM < 1) need on average only 670,000 unique genomic fragments to allow for detection of a peak in the gene, and this estimate was similar when varying the fraction of peaks required to be discovered or TPM thresholds (Supplementary Fig. 11h-j). As ENCODE eCLIP datasets have a mean sequencing depth of over 4.3 million unique genomic fragments, these results suggest that an inability to detect peaks on lowly expressed genes is not a major concern in eCLIP data sequenced to standard depths.

Our analysis above indicates that continued sequencing until fragment saturation can recover true peaks even at extremely high read depths. However, sequencing until fragment saturation is not typically economically reasonable. Thus, we set out to quantify diminishing returns upon deeper sequencing to identify whether we were observing saturation of detected peaks at typical eCLIP sequencing depths (Supplementary Fig. 11k-m). First, we developed a metric to quantify the diminishing returns of deeper sequencing in eCLIP datasets. Considering the discovery of significant peaks, we queried how many peaks were newly discovered when comparing peaks observed when 90% or 100% of fragments in a dataset were used to identify peaks. We observed that 67% of experiments passing manual QC saturated the discovery of significant peaks (defined as the discovery of fewer than 5% new peaks in the above metric), suggesting that simple peak detection was saturating for most but not all high-quality datasets (Supplementary Fig. 11k).

Next, we considered whether binding information by total information content was saturating even when peak discovery was not. Summing the total information content across all peaks, we observed that information recovered saturated for 97% of manually accepted datasets (using the same 5% or less discovery metric between 90% and 100% of fragments used to call peaks in a dataset) (Supplementary Fig. 11k). Exploring downsampling experiments further, we found that 90% of all eCLIP datasets that passed manual quality assessment had saturated information discovery by 8.5M unique fragments (corresponding to 4.3M unique genomic fragments) (Supplementary Fig. 11l-m). Thus, these results suggest that although additional peaks can be identified, the majority of peak information content is already captured at current sequencing depths for the majority of eCLIP experiments described here.

Transcriptome coverage of eCLIP and knockdown/RNA-seq events

Considering the total coverage of gene expression changes across all 472 knockdown/RNA-seq datasets, observed that 20,542 genes were differentially expressed in at least one knockdown experiment, including 92.1% of genes expressed in both cell types and 91.8% of those

expressed in at least one of the two (Extended Data Fig. 2g & Supplementary Fig. 12a-b). Similarly, 17,839 genes had a peak in at least one eCLIP dataset, including 84.2% of genes expressed in both cell types and 92.0% of those expressed in at least one. However, only 4,889 genes had eCLIP peaks from and were responsive to knockdown of the same RBP (Extended Fig. 2g), consistent with previous observations that only a relatively minor subset of RBPs affect RNA stability.

Considering eCLIP alone, 25.8 Mb (2.6%) of annotated pre-mRNA transcripts were covered by at least one reproducible eCLIP peak, representing 10.2 Mb (18.5%) of exonic and 15.6 Mb (1.7%) of intronic sequence (Supplementary Fig. 12c). Restricting our analysis to genes expressed (TPM>1) in both cell types, 3.4% of intronic sequence (2.4% of distal intronic, 4.3% of proximal intronic, and 17.9% of splice site), and 33.5% of exonic sequences (39.0% of 5′UTR, 40.6% of CDS, and 23.3% of 3′UTR, respectively) were covered by at least one peak (Extended Data Fig. 2i & Supplementary Fig. 12d). While profiling a new RBP identified more novel sites than re-profiling the same RBP in the other (K562 or HepG2) cell type, re-profiling the same RBP in a more distinct cell type (H1 or H9 stem cells) yielded even greater increases, suggesting that many additional RBP binding sites remain to be detected (Supplementary Fig. 12e-g). While these results are consistent with previous work suggesting that RNAs are often densely coated by RBPs[17], we note that many of the RBPs with large numbers of peaks include proteins that coat or transiently interact with RNAs as part of basic RNA processing functions, such as interaction of spliceosomal components (e.g. U2AF1/2, SF3B4) with the 5′ and 3′ splice sites, RNA Polymerase II component POLR2G with pre-mRNAs, ribosomal subunit RPS3 with translating RNAs, and various others, rather than likely marking alternative RNA processing regulatory sites.

Analysis of eCLIP correlation with knockdown-perturbed splicing changes (splicing maps)

Splicing maps depicting differential eCLIP enrichment at stereotypical positions relative to RBP knockdown-altered splicing changes were generated using the RBP-Maps methodology[11]. First, the set of differentially alternatively spliced events of the desired type (cassette/skipped exons (SE), alternative 5′ splice site (A5SS), or alternative 3′ splice site (A3SS) events were identified (Supplementary Fig. 13a), requiring rMATS p-value < 0.05, FDR < 0.1, and $|\Delta\Psi| > 0.05$ in knockdown versus control RNA-seq. To eliminate potential double counting of CLIP densities, overlapping AS events were additionally filtered to choose only the

events containing the highest average inclusion junction count (IJC) among all replicates (using the bedtools v2.26 command merge (-o collapse -c 4) and pybedtools 0.7.9).

Next, for each splicing event, per-position input probability densities were subtracted from IP probability densities to attain position-level enrichment or depletion, for regions extending 50nt into each exon and 300nt into each intron composing the event. Subtracted read densities were then normalized to sum to 1 across each event in order to equally weigh each event, creating tracks referred to as 'Normalized eCLIP enrichment' (Supplementary Fig. 13b). For shorter exons (<100 nt) and introns (<600nt), densities were only counted until the boundary of the neighboring feature. Skipped exon (SE) maps were plotted using eCLIP densities overlapping the following 4 regions around AS events: 3′ end of the upstream exon, 5′ end of the cassette, 3′ end of the cassette, and 5′ end of the downstream exon. Alternative 3′ splice site (A3SS) maps were defined with three regions: 3′ end of the upstream exon, 5′ end of the longer transcript, and the 5′ end of the shorter transcript. Alternative 5′ splice site (A5SS) maps were defined with three regions: 3′ end of the shorter transcript, 3′ end of the longer transcript, and the 5′ end of the downstream exon.

Plots of eCLIP signal enrichment (referred to as 'splicing maps') were then created by calculating the mean and standard error of the mean over all events after removing the highest (2.5%) and lowest (2.5%) outlying signal at each position, referred to as 'Average eCLIP enrichment' (Supplementary Fig. 13c). Splicing maps were only considered for RBPs with 100 or more altered cassette exon events, or 50 or more alternative 5′ or 3′ splice site events, considering knockdown-included and knockdown-excluded events separately. Out of a total of 203 pairings of eCLIP and knockdown/RNA-seq in the same cell type (covering 139 RBPs), this left 92 pairings (72 RBPs) for cassette exons, 27 pairings (22 RBPs) for A3SS, and 20 pairings (18 RBPs) for A5SS. As a background reference for cassette exon comparisons, sets of 1,805 (HepG2) and 2,222 (K562) 'native' cassette exons were identified which had $0.05 < \Psi < 0.95$ in at least half of control shRNA RNA-seq datasets for that cell type. Similar sets of 202 (K562) and 159 (HepG2) native alternative 5′ splice site and 389 (K562) and 352 (HepG2) native alternative 3′ splice site events were identified that had $0.05 < \Psi < 0.95$ in at least half of control shRNA RNA-seq datasets for that cell type. RBP-responsive event eCLIP enrichment was then calculated as eCLIP signal enrichment at RBP-regulated events minus eCLIP signal enrichment at native control events, referred to as 'Enrichment relative to control events' (Supplementary Fig. 13d). To calculate significance, 1000 random samplings were performed from the native cassette exon set using the number of events in the knockdown-included (or excluded), and

significance was set as being either lower than the 0.5^{th} or higher than the 99.5^{th} percentile for each position.

Supplementary Data

Supplementary Data 1. Data summary and manual annotation of RBP functions. For each RBP profiled by at least one assay in this study, table includes experiments performed, RNA localization observed, presence of annotated RNA binding domains, and manual literature-based annotation of RBP function.

Supplementary Data 2. ENCODE accession identifiers of datasets used. Tabs contain accession identifiers (for the ENCODE Data Coordination Center, https://www.encodeproject.org/) for all datasets generated in this study.

Supplementary Data 3. RBP gene expression in ENCODE cell lines and tissues. Contains RNA expression (in transcripts per million reads) for all RBPs profiled in this study for K562 cells, HepG2 cells, and 40 human tissues profiled by the GTEx consortium.

Supplementary Data 4. Summary information for eCLIP experiments. Contains summary information for all eCLIP experiments, including antibodies (accession identifiers, catalog, and lot numbers) as well as general sequencing library information (number of input reads, PCR duplication rate, and number of significantly enriched peaks).

Supplementary Data 5. Summary information for RNA-seq experiments. Contains summary information for RBP knockdown/RNA-seq experiments, including number of differentially expressed genes and alternative splicing events.

Supplementary Data 6. Summary information for RBNS experiments. Contains summary information for RNA Bind-N-Seq (RBNS) experiments, including reaction conditions (temperature and read length) as well as primary analysis results (enriched consensus motifs).

Supplementary Data 7. Summary information for ChIP-seq experiments. Contains summary information of ChIP-seq experiments, including number of usable reads and significantly enriched peaks, as well as experimental quality assessment metrics (PCR bottleneck coefficient, normalized and relative strand cross-correlation, and IDR reproducibility).

Supplementary Data 8. Automated and manual quality assessment of eCLIP datasets. Contains results from automated eCLIP quality assessment pipeline, including read number, total relative information, and IDR rescue and self-consistency ratio metrics. Tabs include 223 released datasets, 76 rejected datasets, and 50 datasets with reproducible signal of questionable reliability made available on the Gene Expression Omnibus.

Supplementary Data 9. Summary information for questionable quality eCLIP experiments. Contains summary information for 50 eCLIP experiments with reproducible signal of questionable reliability, including antibodies (accession identifiers, catalog, and lot numbers) as well as general sequencing library information (number of input reads, PCR duplication rate, and number of significantly enriched peaks).

Supplementary Data 10. Summary information for eCLIP experiments failing quality assessment. Contains summary information for 76 eCLIP experiments which failed manual quality assessment, including antibodies (accession identifiers, catalog, and lot numbers) as well as general sequencing library information (number of input reads, PCR duplication rate, and number of significantly enriched peaks).

Supplementary Data 11. eCLIP blacklist regions. 56 regions which showed consistent artifact signal across many eCLIP experiments and were excluded from analyses.

Supplementary Data 12. Overlap between eCLIP and ChIP-seq peaks. Contains statistics of overlap between eCLIP and ChIP-seq peaks.

Supplementary Data 13. eCLIP adapters used. Contains sequences of in-line barcoded RNA adapters used for eCLIP experiments.

Supplementary Figures

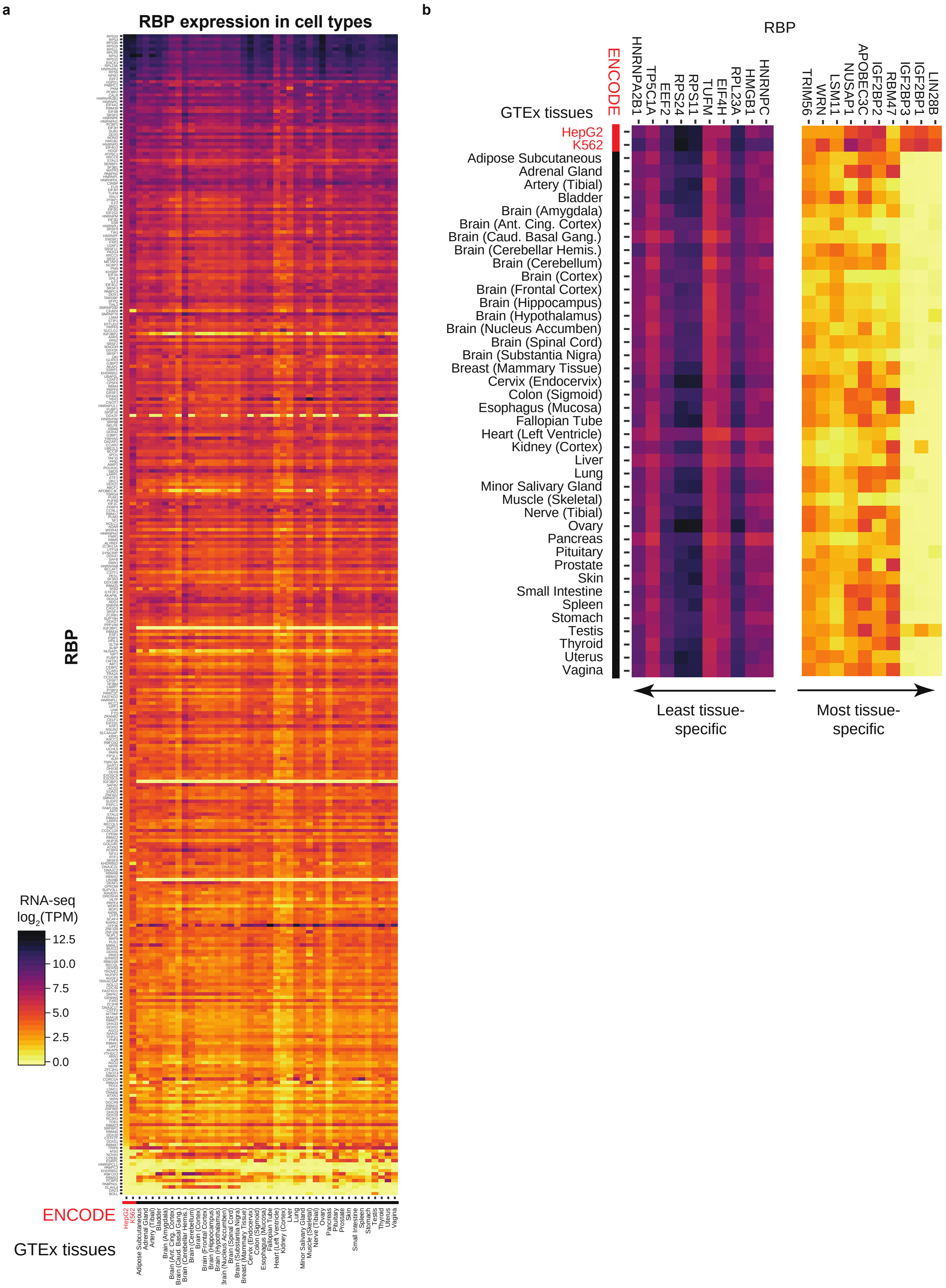
a
RBP expression in cell types
RBP
RNA-seq
log2(TPM)
12.5
10.0
7.5
5.0
2.5
0.0
ENCODE
GTEx tissues
b
RBP
ENCODE
GTEx tissues
HNRNPA2B1
TP53
C1A
EEF2
RPS24
RPS11
TUFM
EIF4H
RPL23A
HMGB1
HNRNPC
TRIM56
WRN
LSM11
NUSAP1
APOBEC3C
IGF2BP2
RBM47
IGF2BP3
IGF2BP1
LIN28B
HepG2
K562
Adipose Subcutaneous
Adrenal Gland
Artery (Tibial)
Bladder
Brain (Amygdala)
Brain (Ant. Cing. Cortex)
Brain (Caud. Basal Gang.)
Brain (Cerebellar Hemis.)
Brain (Cerebellum)
Brain (Cortex)
Brain (Frontal Cortex)
Brain (Hippocampus)
Brain (Hypothalamus)
Brain (Nucleus Accumben)
Brain (Spinal Cord)
Brain (Substantia Nigra)
Breast (Mammary Tissue)
Cervix (Endocervix)
Colon (Sigmoid)
Esophagus (Mucosa)
Fallopian Tube
Heart (Left Ventricle)
Kidney (Cortex)
Liver
Lung
Minor Salivary Gland
Muscle (Skeletal)
Nerve (Tibial)
Ovary
Pancreas
Pituitary
Prostate
Skin
Small Intestine
Spleen
Stomach
Testis
Thyroid
Uterus
Vagina
Least tissue-specific
Most tissue-specific

Supplementary Figure 1 | Expression of RBPs across tissues and cell types.

(a) Expression of the 356 RBPs (in Transcripts Per Million) investigated in this study in ENCODE cell lines HepG2 and K562 as well as 40 human tissues measured by the GTEx project. RBPs sorted by decreasing expression in HepG2.

(b) Expression of the 10 RBPs with the highest and lowest tissue-specificity across the two ENCODE cell lines and 40 human tissues.

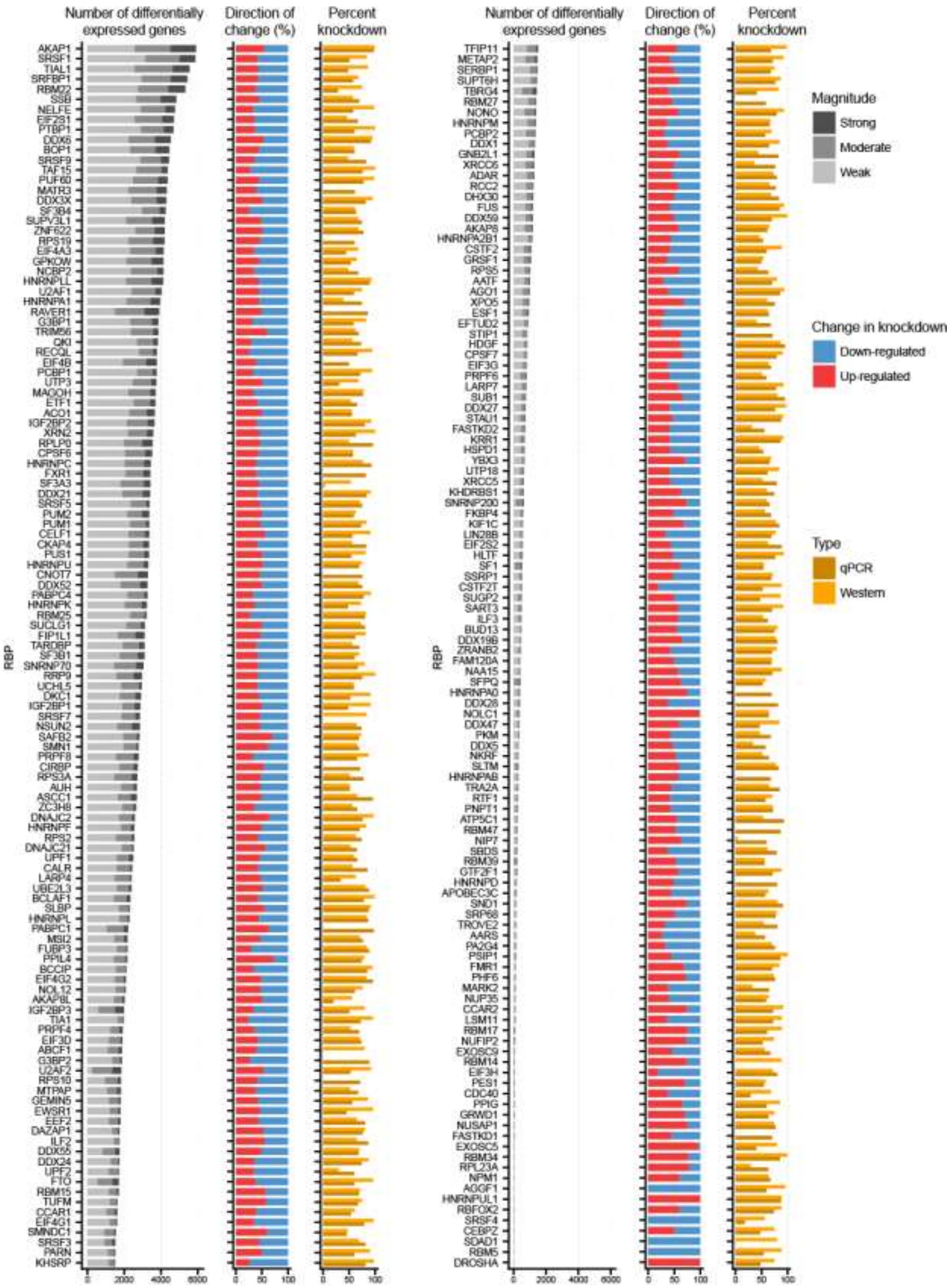

Number of differentially expressed genes
Direction of change (%)
Percent knockdown
RBP
AKAP1
SRSF1
TIAL1
SRFBP1
RBM22
SSB
NELFE
EIF2S1
PTBP1
DDX6
BOP1
SRSF9
TAF15
PUF60
MATR3
DDX3X
SF3B4
SUPV3L1
ZNF622
RPS19
EIF4A3
GPKOW
NCBP2
HNRNPLL
U2AF1
HNRNPA1
RAVER1
G3BP1
TRIM56
QKI
RECQL
EIF4B
PCBP1
UTP3
MAGOH
ETF1
ACO1
IGF2BP2
XRN2
RPLP0
CPSF6
HNRNPC
FXR1
SF3A3
DDX21
SRSF5
PUM2
PUM1
CELF1
CKAP4
PUS1
HNRNPU
CNOT7
DDX52
PABPC4
HNRNPK
RBM25
SUCLG1
FIP1L1
TARDBP
SF3B1
SNRNP70
RRP9
UCHL5
DKC1
IGF2BP1
SRSF7
NSUN2
SAFB2
SMN1
PRPF8
CIRBP
RPS3A
AUH
ASCC1
ZC3H8
DNAJC2
HNRNPF
RPS2
DNAJC21
UPF1
CALR
LARP4
UBE2L3
BCLAF1
SLBP
HNRNPL
PABPC1
MSI2
FUBP3
PPIL4
BCCIP
EIF4G2
NOL12
AKAP8L
IGF2BP3
TIA1
PRPF4
EIF3D
ABCF1
G3BP2
U2AF2
RPS10
MTPAP
GEMIN5
EWSR1
EEF2
DAZAP1
ILF2
DDX55
DDX24
UPF2
FTO
RBM15
TUFM
CCAR1
EIF4G1
SMNDC1
SRSF3
PARN
KHSRP
0
2000
4000
6000
0
50
100
0
50
100
Number of differentially expressed genes
Direction of change (%)
Percent knockdown
RBP
TFIP11
METAP2
SERBP1
SUPT6H
TBRG4
RBM27
NONO
HNRNPM
PCBP2
DDX1
GNB2L1
XRCC6
ADAR
RCC2
DHX30
FUS
DDX59
AKAP8
HNRNPA2B1
CSTF2
GRSF1
RPS5
AATF
AGO1
XPO5
ESF1
EFTUD2
STIP1
HDGF
CPSF7
EIF3G
PRPF6
LARP7
SUB1
DDX27
STAU1
FASTKD2
KRR1
HSPD1
YBX3
UTP18
XRCC5
KHDRBS1
SNRNP200
FKBP4
KIF1C
LIN28B
EIF2S2
HLTF
SF1
SSRP1
CSTF2T
SUGP2
SART3
ILF3
BUD13
DDX19B
ZRANB2
FAM120A
NAA15
SFPQ
HNRNPA0
DDX28
NOLC1
DDX47
PKM
DDX5
NKRF
SLTM
HNRNPAB
TRA2A
RTF1
PNPT1
ATP5C1
RBM47
NIP7
SBDS
RBM39
GTF2F1
HNRNPD
APOBEC3C
SND1
SRP68
TROVE2
AARS
PA2G4
PSIP1
FMR1
PHF6
MARK2
NUP35
CCAR2
LSM11
RBM17
NUFIP2
EXOSC9
RBM14
EIF3H
PES1
CDC40
PPIG
GRWD1
NUSAP1
FASTKD1
EXOSC5
RBM34
RPL23A
NPM1
AGGF1
HNRNPUL1
RBFOX2
SRSF4
CEBPZ
SDAD1
RBM5
DROSHA
0
2000
4000
6000
0
50
100
0
50
100
Magnitude
Strong
Moderate
Weak
Change in knockdown
Down-regulated
Up-regulated
Type
qPCR
Western

Supplementary Figure 2 | Gene expression changes upon RBP knockdown in HepG2 cells.

Each row indicates summary statistics for an RBP knockdown followed by RNA-seq dataset in HepG2 cells. Bars indicate (left) the number and magnitude of differentially expressed genes, (center) the type of regulation and (right) the knockdown level of the targeted RBP protein and/or mRNA. The magnitudes of differential expression were defined as strong (fold-change ≥ 4), moderate (2 < fold-change < 4) and weak (fold-change ≤ 2). (center) Bars indicate the fraction of differentially expressed genes (red) increased or (blue) decreased upon RBP knockdown. (right) Bars indicate the percent knockdown of the RBP mRNA observed by qPCR and protein observed by Western blot analysis.

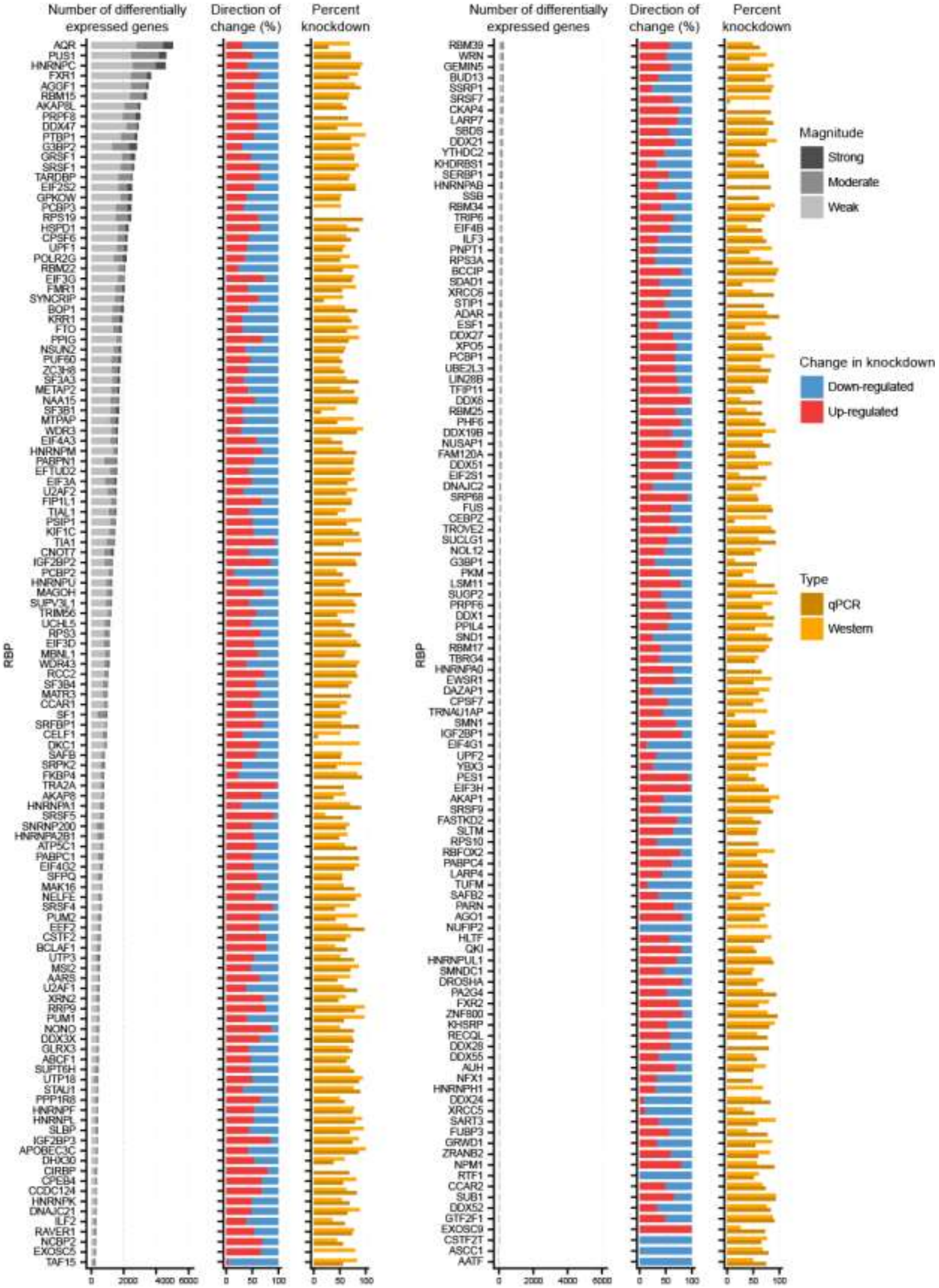
Number of differentially expressed genes
Direction of change (%)
Percent knockdown
RBP
AQR
PUS1
HNRNPC
FXR1
AGGF1
RBM15
AKAP8L
PRPF8
DDX47
PTBP1
G3BP2
GRSF1
SRSF1
TARDBP
EIF2S2
GPKOW
PCBP3
RPS19
HSPD1
CPSF6
UPF1
POLR2G
RBM22
EIF3G
FMR1
SYNCRIP
BOP1
KRR1
FTO
PPIG
NSUN2
PUF60
ZC3H8
SF3A3
METAP2
NAA15
SF3B1
MTPAP
WDR3
EIF4A3
HNRNPM
PABPN1
EFTUD2
EIF3A
U2AF2
FIP1L1
TIAL1
PSIP1
KIF1C
TIA1
CNOT7
IGF2BP2
PCBP2
HNRNPU
MAGOH
SUPV3L1
TRIM56
UCHL5
RPS3
EIF3D
MBNL1
WDR43
RCC2
SF3B4
MATR3
CCAR1
SF1
SRFBP1
CELF1
DKC1
SAFB
SRPK2
FKBP4
TRA2A
AKAP8
HNRNPA1
SRSF5
SNRNP200
HNRNPA2B1
ATP5C1
PABPC1
EIF4G2
SFPQ
MAK16
NELFE
SRSF4
PUM2
EEF2
CSTF2
BCLAF1
UTP3
MSI2
AARS
U2AF1
XRN2
RRP9
PUM1
NONO
DDX3X
GLRX3
ABCF1
SUPT6H
UTP18
STAU1
PPP1R8
HNRNPF
HNRNPL
SLBP
IGF2BP3
APOBEC3C
DHX30
CIRBP
CPEB4
CCDC124
HNRNPK
DNAJC21
ILF2
RAVER1
NCBP2
EXOSC5
TAF15
0
2000
4000
6000
0
50
100
0
50
100
Number of differentially expressed genes
Direction of change (%)
Percent knockdown
RBP
RBM39
WRN
GEMIN5
BUD13
SSRP1
SRSF7
CKAP4
LARP7
SBDS
DDX21
YTHDC2
KHDRBS1
SERBP1
HNRNPAB
SSB
RBM34
TRIP6
EIF4B
ILF3
PNPT1
RPS3A
BCCIP
SDAD1
XRCC6
STIP1
ADAR
ESF1
DDX27
XPO5
PCBP1
UBE2L3
LIN28B
TFIP11
DDX6
RBM25
PHF6
DDX19B
NUSAP1
FAM120A
DDX51
EIF2S1
DNAJC2
SRP68
FUS
CEBPZ
TROVE2
SUCLG1
NOL12
G3BP1
PKM
LSM11
SUGP2
PRPF6
DDX1
PPIL4
SND1
RBM17
TBRG4
HNRNPA0
EWSR1
DAZAP1
CPSF7
TRNAU1AP
SMN1
IGF2BP1
EIF4G1
UPF2
YBX3
PES1
EIF3H
AKAP1
SRSF9
FASTKD2
SLTM
RPS10
RBFOX2
PABPC4
LARP4
TUFM
SAFB2
PARN
AGO1
NUFIP2
HLTF
QKI
HNRNPUL1
SMNDC1
DROSHA
PA2G4
FXR2
ZNF800
KHSRP
RECQL
DDX28
DDX55
AUH
NFX1
HNRNPH1
DDX24
XRCC5
SART3
FUBP3
GRWD1
ZRANB2
NPM1
RTF1
CCAR2
SUB1
DDX52
GTF2F1
EXOSC9
CSTF2T
ASCC1
AATF
0
2000
4000
6000
0
50
100
0
50
100
Magnitude
Strong
Moderate
Weak
Change in knockdown
Down-regulated
Up-regulated
Type
qPCR
Western

Supplementary Figure 3 | Gene expression changes upon RBP knockdown in K562 cells. Each row indicates summary statistics for an RBP knockdown followed by RNA-seq dataset in K562 cells. Bars indicate (left) the number and magnitude of differentially expressed genes, (center) the type of regulation and (right) the knockdown level of the targeted RBP protein and/or mRNA. The magnitudes of differential expression were defined as strong (fold-change ≥ 4), Moderate (2 < fold-change < 4) and weak (fold-change ≤ 2). (center) Bars indicate the fraction of differentially expressed genes (red) increased or (blue) decreased upon RBP knockdown. (right) Bars indicate the percent knockdown of the RBP mRNA observed by qPCR and protein observed by Western blot analysis.

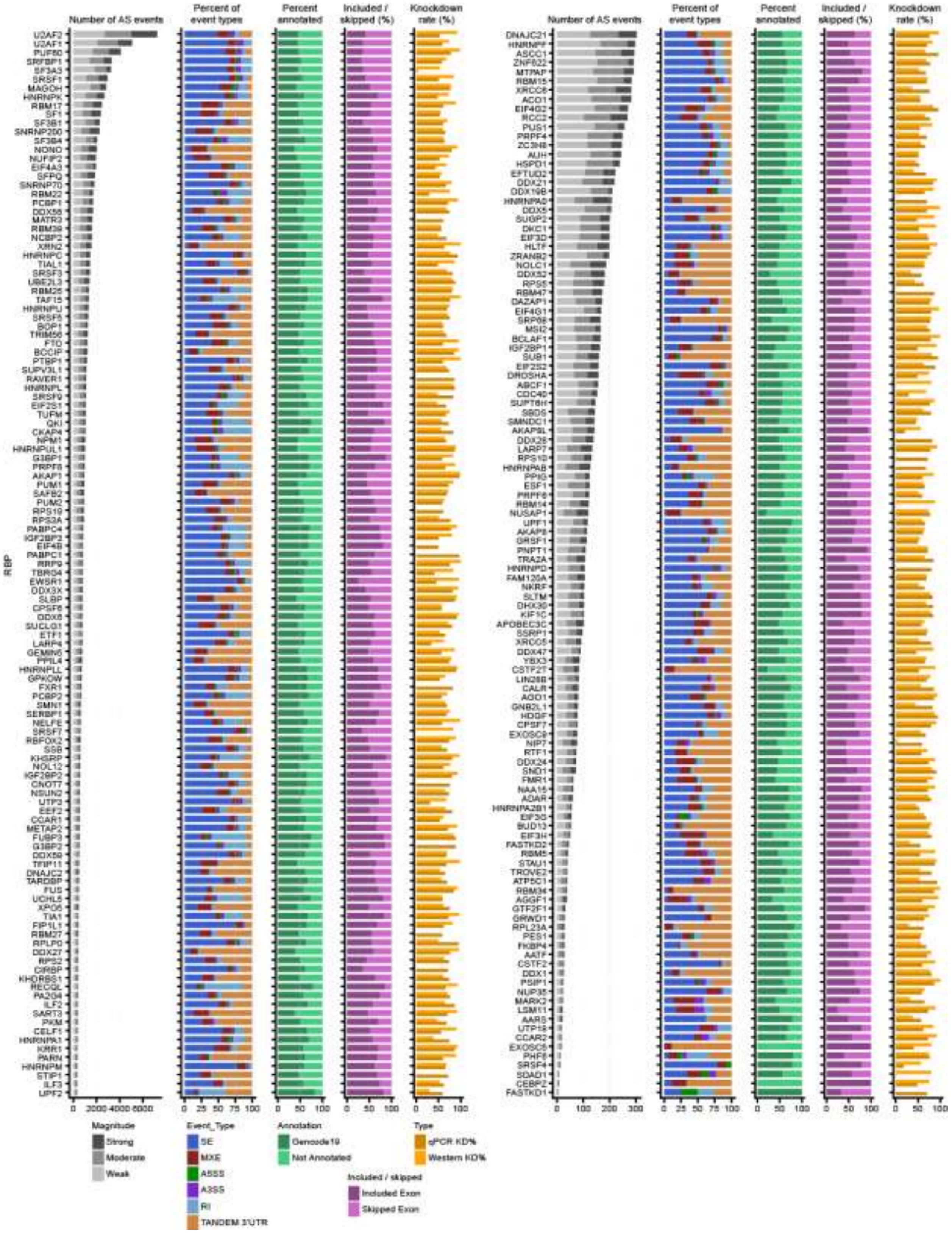

Supplementary Figure 4 | Alternative splicing changes upon RBP knockdown RNA-seq in HepG2 cells.

Each row indicates summary alternative splicing statistics for an RBP knockdown followed by RNA-seq dataset in HepG2 cells. Bars indicate (left) the number and magnitude of differentially spliced events, (center-left) the fraction of each type of alternative splicing event, (center) the percent of events observed that are present in GENCODE v19, (center-right) the fraction of cassette exons that are either differentially included or excluded, and (right) the knockdown level of the targeted RBP mRNA and protein by qPCR and Western blot analysis. The magnitudes of differential splicing were defined as weak ($|\Delta\Psi|$ = 5% - 15%), moderate ($|\Delta\Psi|$ = 15% - 30%) or strong ($|\Delta\Psi|$ >= 30. The affected alternative event types are SE (skipped exon), MXE (mutually exclusive exons), A5SS (alternative 5′ splice site), A3SS (alternative 3′ splice site), RI (retained intron) and TANDEMUTR (tandem 3′UTR).

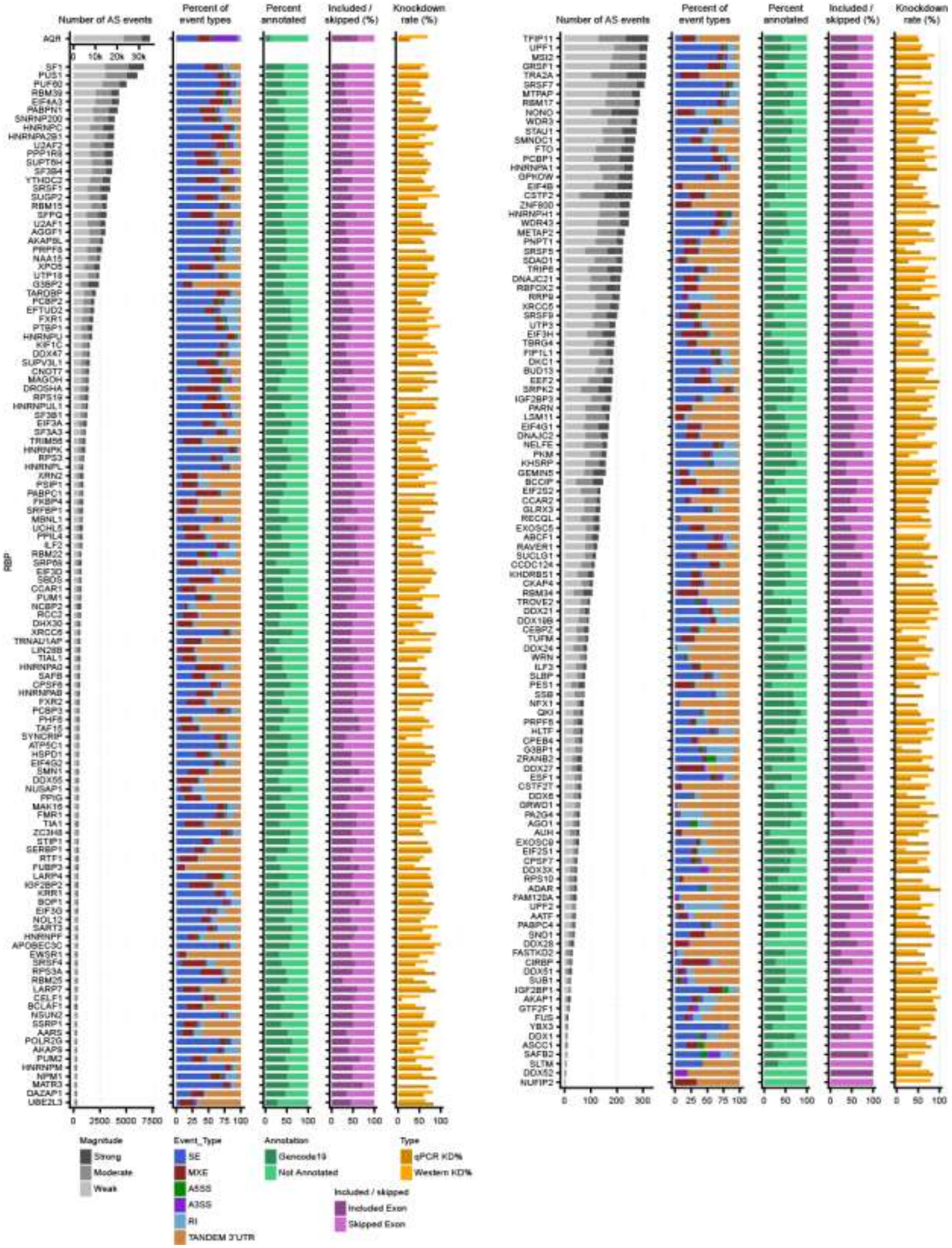
Number of AS events
Percent of event types
Percent annotated
Included / skipped (%)
Knockdown rate (%)
RBP
Magnitude
Strong
Moderate
Weak
Event_Type
SE
MXE
A5SS
A3SS
RI
TANDEM 3'UTR
Annotation
Gencode19
Not Annotated
Included / skipped
Included Exon
Skipped Exon
Type
qPCR KD%
Western KD%

Supplementary Figure 5 | Alternative splicing changes upon RBP knockdown RNA-seq in K562 cells.

Each row indicates summary alternative splicing statistics for an RBP knockdown followed by RNA-seq dataset in K562 cells. Bars indicate (left) the number and magnitude of differentially spliced events, (center-left) the fraction of each type of alternative splicing event, (center) the percent of events observed that are present in GENCODE v19, (center-right) the fraction of cassette exons that are either included or excluded, and (right) the knockdown level of the targeted RBP mRNA and protein by qPCR and Western blot analysis. The magnitudes of differential splicing were defined as weak ($|\Delta\Psi|$ = 5% - 15%), moderate ($|\Delta\Psi|$ = 15% - 30%) or strong ($|\Delta\Psi| >= 30$). The affected alternative event types are SE (skipped exon), MXE (mutually exclusive exons), A5SS (alternative 5′ splice site), A3SS (alternative 3′ splice site), RI (retained intron) and TANDEMUTR (tandem 3′UTR).

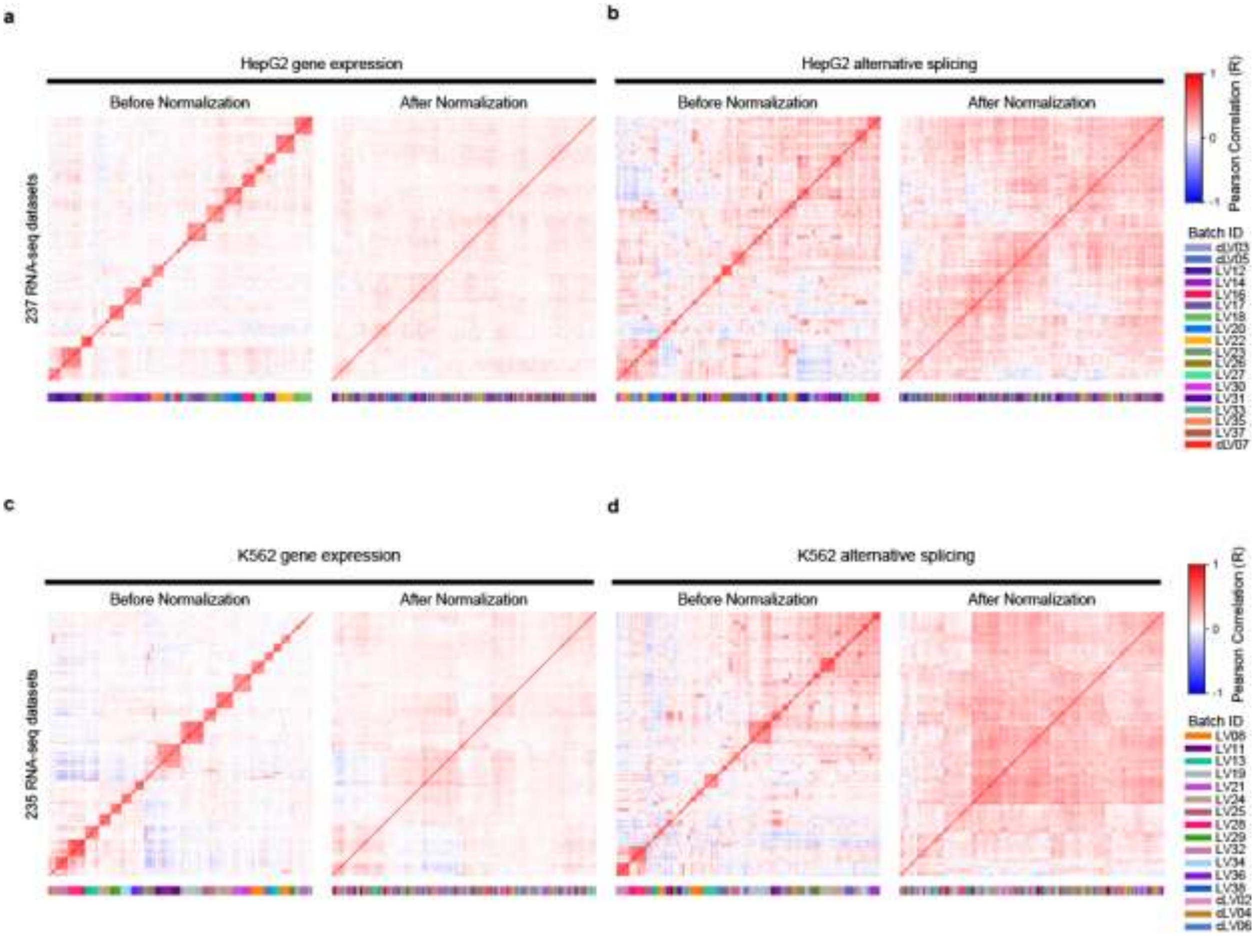

Supplementary Figure 6 | Batch correction of RBP knockdown RNA-seq datasets

(a-d) Heatmaps show Pearson correlation between all RNA-seq datasets before and after normalization to remove batch effects, followed by hierarchical clustering. Analysis was performed separately for (a-b) 266 datasets in HepG2 and (c-d) 264 datasets in K562 cell lines. (a,c) For gene expression, correlation was determined between gene expression fold-change values (log_2) from comparison of (left) RBP knockdown versus paired control or (right) performing batch correction on all datasets followed by comparing RBP knockdown replicates versus a ‘virtual control’ defined as the average of all replicate 1 or replicate 2 control experiments respectively (as described in Methods). (b,d) For splicing, correlation was calculated between change in exon inclusion values between (left) RBP knockdown and within-batch control experiments, and (right) RBP knockdown versus a ‘virtual control’ defined as the average of all replicate 1 or replicate 2 control samples respectively following batch correction of junction read counts as described in Methods. For all, colors below indicate experimental batches.

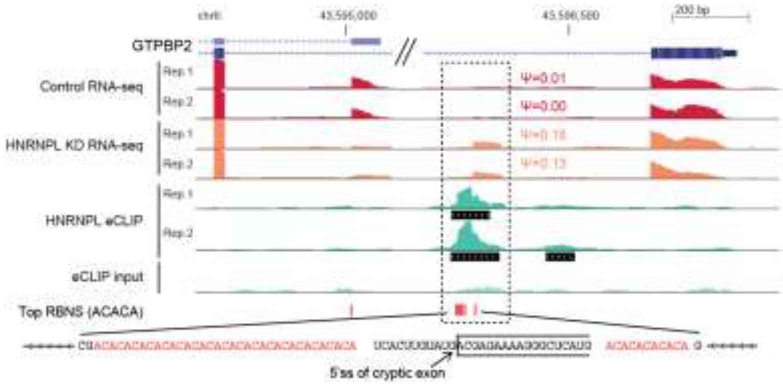

Supplementary Figure 7 | HNRNPL regulation of GTPBP2 cryptic exon

Shown is RNA-seq read density (reads per million), eCLIP read density (reads per million), and RBNS motif presence proximal to a 73-nt cryptic exon expected to induce nonsense-mediated decay (NMD) of GTP Binding Protein 2 (a ribosome rescue factor whose loss induces neurodegeneration in certain genetic backgrounds[18]). eCLIP indicates that HNRNPL binds over the cryptic exon 5′ splice site in a sequence-specific manner to a region rich in the top RBNS 5mer, ACACA.

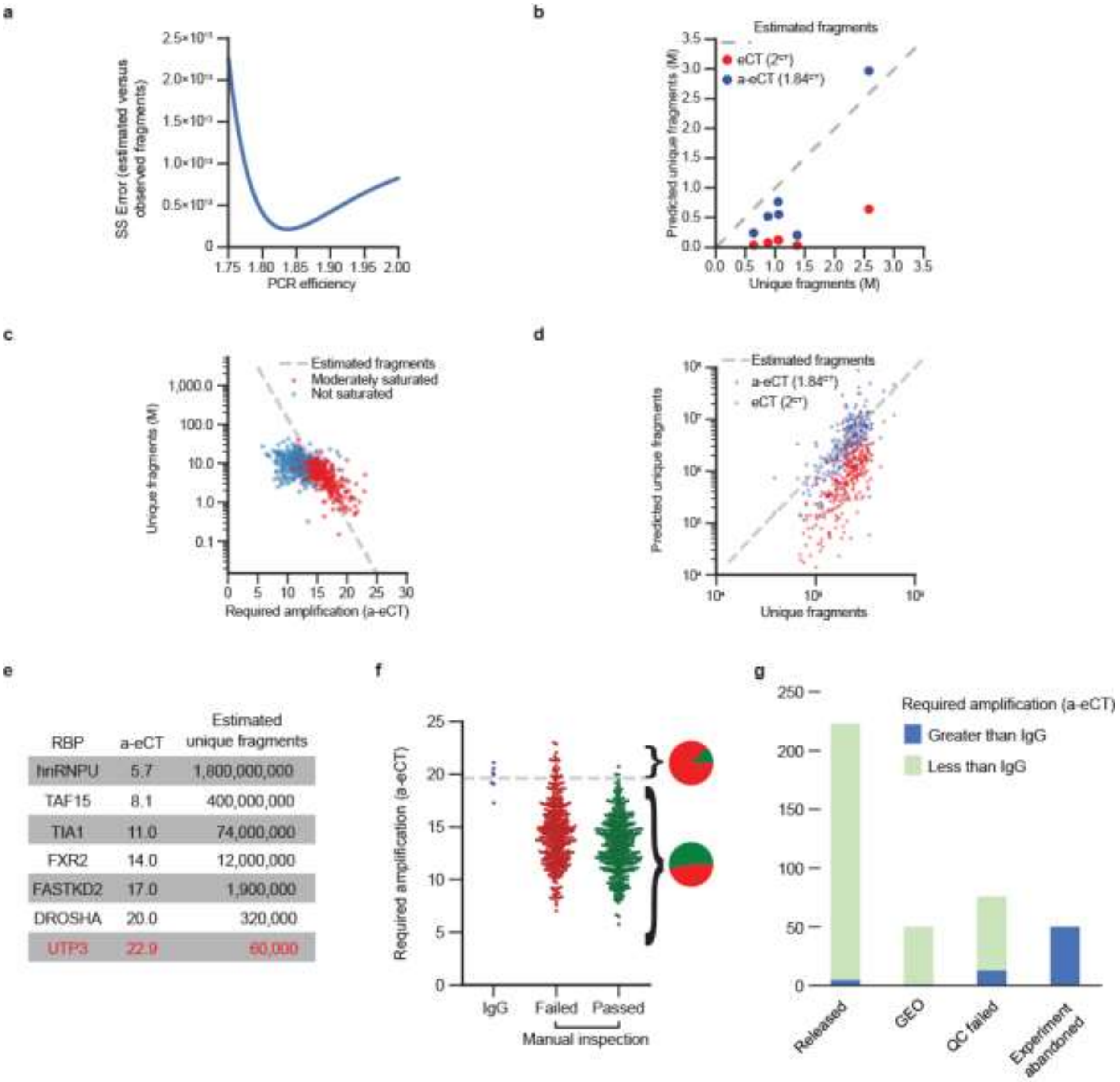

Supplementary Figure 8 | Estimation of unique RNA molecules present in eCLIP libraries

(a) Plot indicates sum of squared error for varying PCR efficiency when comparing true observed number of unique molecules to estimated number of unique molecules for six highly saturated (>90% PCR duplicated) experiments.

(b) Scatter plot of estimated unique molecules at two estimates of PCR efficiency, (red) 2 and (blue) 1.84, versus unique fragments obtained after sequencing for six highly saturated (>90% PCR duplicated) experiments.

(c) Scatter plot indicates accurate-eCT (a-eCT) (see Methods) versus unique fragments observed (including non-PCR duplicate reads mapped either to unique genomic loci or repetitive elements, in millions of reads mapped) for all ENCODE eCLIP experiments. Non-saturated

(<60% PCR duplicates) datasets are indicated in blue, and 276 moderately saturated (>60% PCR duplicates) datasets are indicated in red. Dashed line indicates the number of unique molecules expected based on a-eCT.

(d) Scatter plot of estimated unique molecules at two estimates of PCR efficiency, (red) 2 and (blue) 1.84, versus unique fragments obtained after sequencing. Shown are 276 moderately saturated experiments (>60% PCR duplicated).

(e) Representative RBPs are listed along with their a-eCT and corresponding estimate of the number of unique RNA molecules isolated in eCLIP. UTP3 (in red) did not pass quality control metrics.

(f) Points indicate the a-eCT value of all ENCODE eCLIP experiments, separated into (blue) IgG controls, (red) datasets that failed manual quality assessment, and (green) datasets passing manual assessment. Dotted line indicates average a-eCT of IgG control experiments (19.6).

(g) Bars indicate the distribution of eCLIP datasets (separated into classes as described in (Extended Data Fig. 1c)) with respect to required amplification (a-eCT) relative to IgG controls.

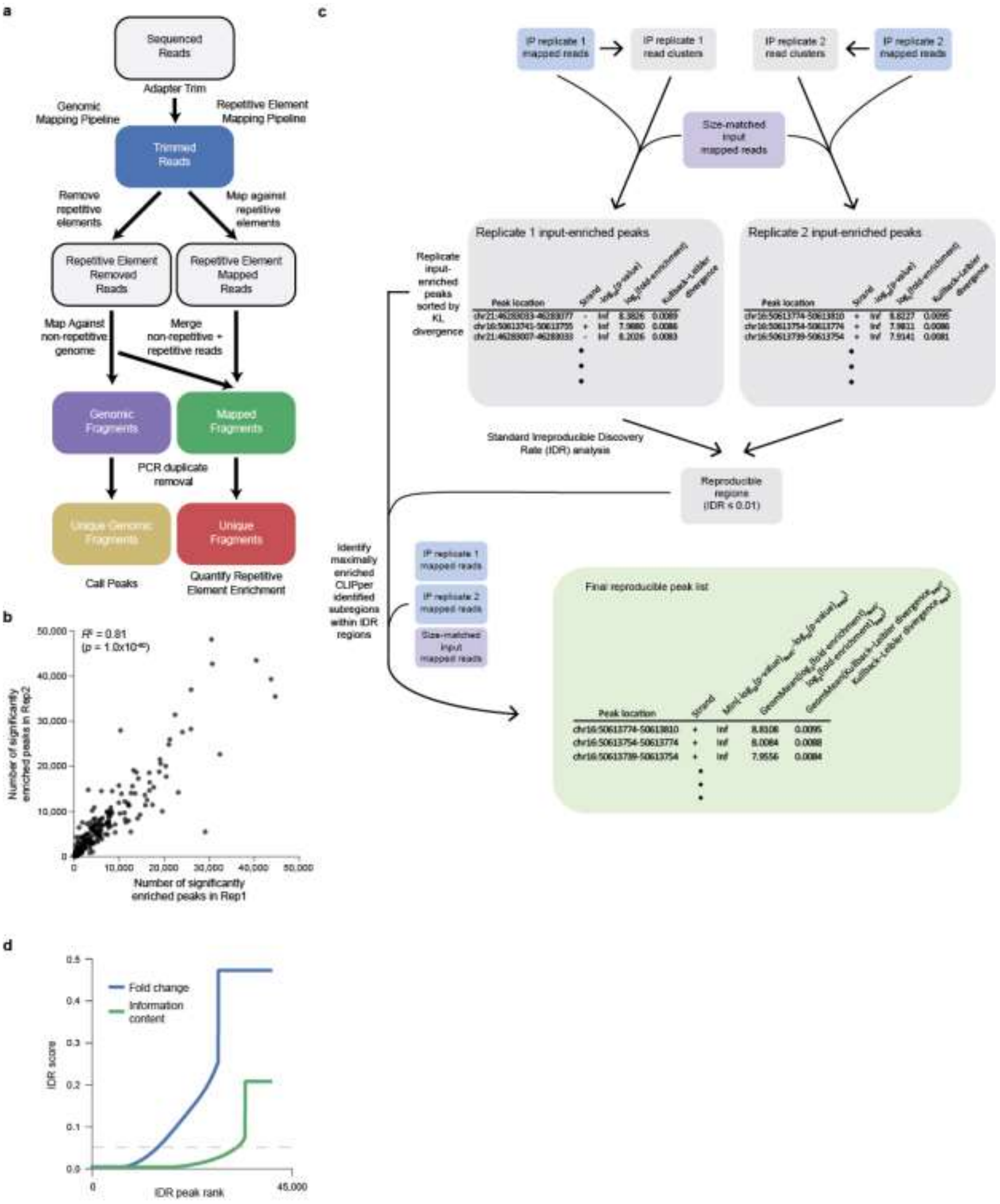

Supplementary Figure 9 | Identification of reproducible eCLIP peaks

(a) Schematic of eCLIP processing for both unique genomic mapping and repetitive element mapping.

(b) Points indicate the number of significantly enriched peaks (fold-enrichment ≥ 8 and $p \leq 0.001$ from two-sided Fisher's Exact Test (or Yates' Chi-Square test where appropriate) with no hypothesis testing correction, as described in Methods) identified in replicate 1 versus replicate 2 for each of 223 high-quality eCLIP experiments. Pearson correlation and significance were determined in MATLAB.

(c) Schematic of adaption of Irreproducible Discovery Rate (IDR) analysis to identification of reproducible eCLIP peaks. (top) First, input-normalized clusters are identified separately for two biological replicates. (center) Next, these peaks are ranked by relative information content, defined as $I_i = p_i \times log_2(\frac{p_i}{q_i})$, for proportion of IP reads within peak *i* represented by p_i and fraction of input reads within the peak as q_i. Next, standard IDR analysis is performed on the ranked peak lists to identify reproducible regions at IDR cutoff of 0.01. (bottom) Finally, we considered all CLIPper-identified subregions within these IDR regions, and calculated the fold-enrichment in IP versus input for each subregion in each replicate. Subregions were ranked by the geometric mean of fold-enrichment between the two replicates, and the set of non-overlapping subregions that were significantly enriched ($p \leq 0.001$ in both replicates) with geometric mean of fold-enrichment ≥ 8 in both replicates were obtained as the set of reproducible peaks

(d) Plot indicates each peak ranked by IDR score, when IDR score is calculated by ranking peaks based on (blue) fold-enrichment above input or (green) information content.

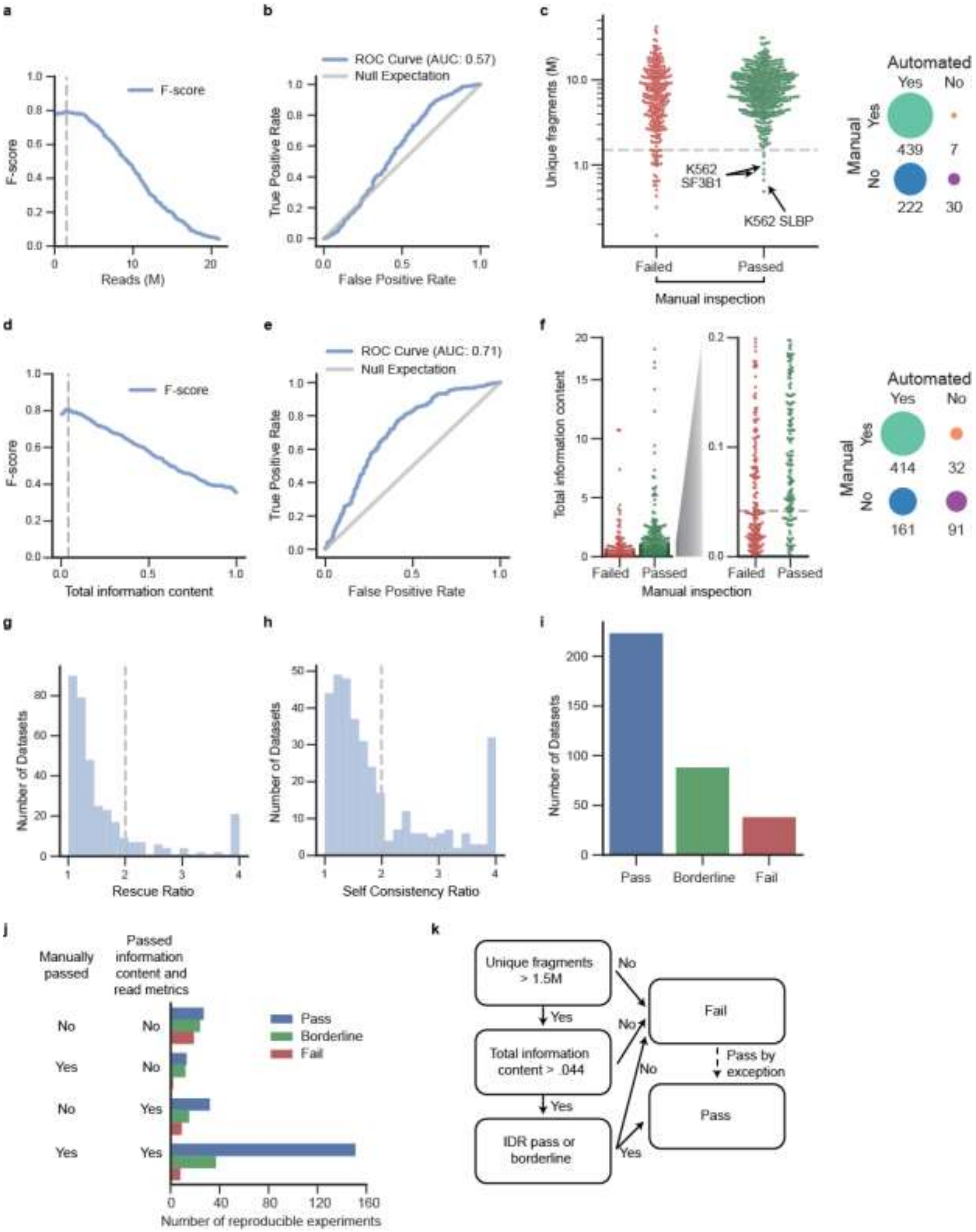

Supplementary Figure 10 | Quality metrics to assay eCLIP data quality and reproducibility

(a) Plot indicates f-score for classification of datasets relative to manual quality assessment based on unique fragments present. Maximal classification of datasets was obtained at a cutoff of 1.5 million unique fragments.

(b) ROC curve for classifying datasets based upon varying minimum unique fragment thresholds for 698 total eCLIP datasets.

(c) Swarm plot indicates number of unique fragments observed in each eCLIP dataset separated by (red) failing or (green) passing manual quality inspection. Dashed line indicates 1.5 million read quality threshold that maximizes predictive power on manual classification (as shown in (a)) and inset indicates confusion matrix for this threshold versus manual inspection. Three datasets judged to be high quality despite low unique fragment number are indicated.

(d) Plot indicates f-score for classification of datasets based on the total information content in all significantly enriched peaks. Only datasets passing the unique fragment cutoff in (a-c) were considered.

(e) ROC curve for classifying datasets based upon varying total information in peak cutoff for 698 total eCLIP datasets.

(f) Swarm plot indicates the total information content across all peaks in each eCLIP dataset that passes the unique fragment threshold in (c), separated by (red) failing or (green) passing manual quality inspection. Dashed line indicates the information content threshold that maximizes predictive power on manual classification (as shown in (d)) and inset indicates confusion matrix for this threshold versus manual inspection.

(g) Bar plot indicates IDR rescue ratio for all ENCODE eCLIP experiments. Dashed line indicates a cutoff of 2 previously used for ChIP-seq analysis.

(h) Bar plot indicates IDR self-consistency ratio for all ENCODE eCLIP experiments. Dashed line indicates a cutoff of 2 previously used for ChIP-seq analysis.

(i) Bars indicate the number of ENCODE eCLIP experiments that either (pass, in blue) pass both rescue ratio and self-consistency ratio, (borderline, in green) passed just one of the two tests, or (fail, in red) failed both tests.

(j) Bar chart indicates the count of all ENCODE experiments that pass or fail manual or automated QC approaches, broken into three groups based on their IDR thresholding metric status: (blue) passed, (green) borderline, and (red) failed.

(k) Schematic detailing final recommended quality assessment decision flowchart.

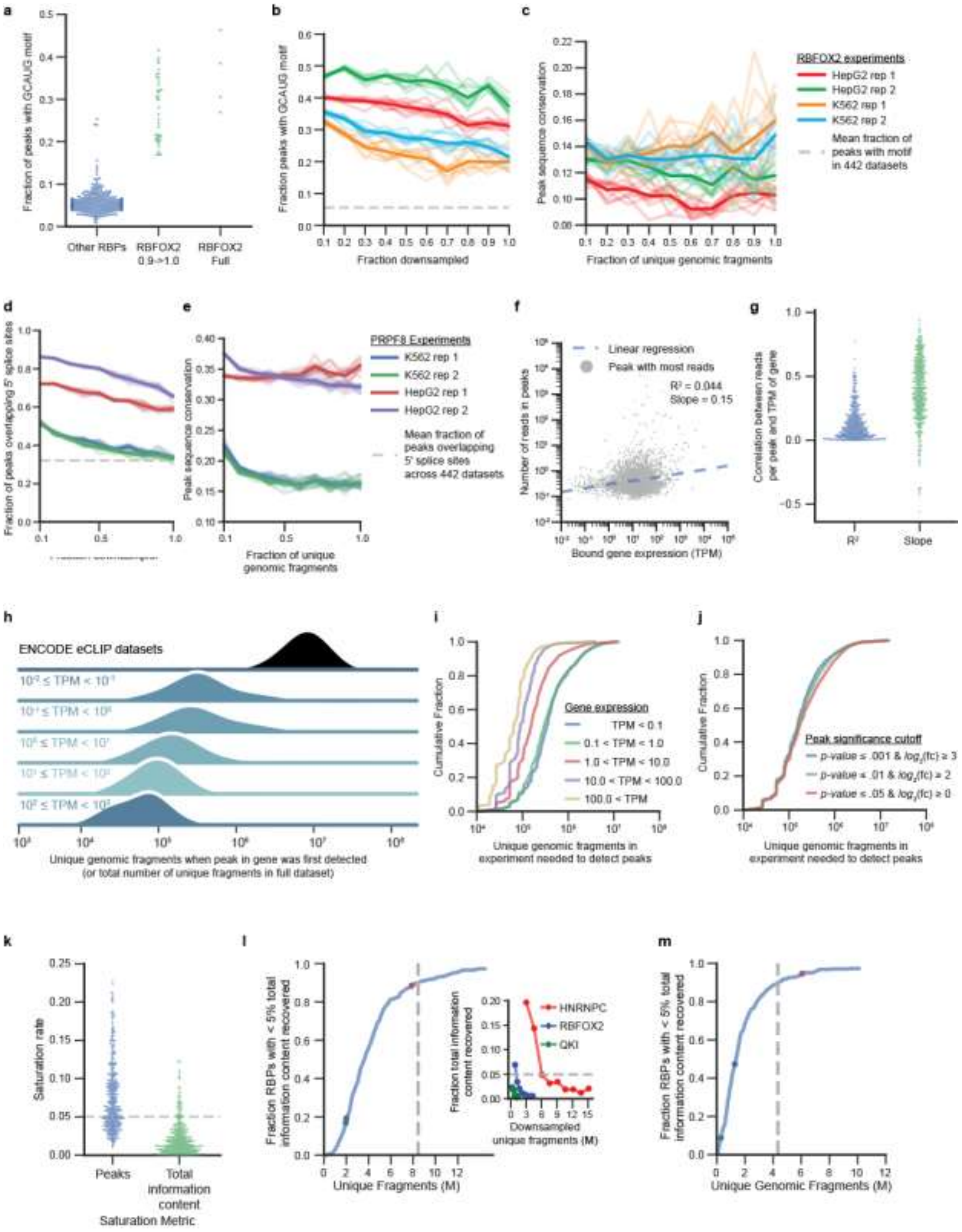

Supplementary Figure 11 | Analysis of eCLIP signal detection based on sequencing depth

(a) Points indicate the fraction of significant peaks that contain a GCAUG motif. Shown are (blue) all RBPs except RBFOX2, (green) peaks newly identified when comparing the 90% subsampled to full RBFOX2 dataset (for 10 random subsamplings), and (red) the full RBFOX2 datasets (2 replicates each for K562 and HepG2 cells).

(b) Plot indicates the fraction of peaks containing a GCAUG motif for peaks identified in a series of subsamples of eCLIP unique genomic fragments for RBFOX2 in HepG2 and K562. Shown are RBFOX2 HepG2 (red) replicate 1 and (green) replicate 2, and RBFOX2 K562 (orange) replicate 1 and (blue) replicate 2. The dashed grey line indicates the mean fraction of GCAUG-containing peaks observed across all 223 released eCLIP datasets. Dark lines indicate mean across 10 downsampling iterations.

(c) Plot indicates mean mammalian phastons conservation for all peaks newly discovered in each downsampled subsample for RBFOX2 eCLIP in HepG2 (red) replicate 1 and (green) replicate 2 and K562 (orange) replicate 1 and (blue) replicate 2. Dark lines indicate mean across 10 downsampling iterations.

(d-e) Downsampling analysis for PRPF8 eCLIP in HepG2 (blue) replicate 1 and (green) replicate 2, and K562 (red) replicate 1 and (purple) replicate 2. Dark lines indicate mean across 10 downsampling iterations. (d) Plot indicates the fraction of peaks newly discovered in each downsampled subsample that overlap the 5′ splice site. The dashed grey line indicates mean fraction overlap with 5′ splice sites for all 512 non-PRPF8 released ENCODE datasets. (e) Lines indicate the average conservation for newly discovered peaks at the indicated downsampling fraction.

(f) One point for each gene (n = 2816) indicates the TPM (Transcripts Per Million reads) of the gene (x-axis) and the number of reads (normalized by peak size) in the peak with the highest number of reads for RBFOX2 in HepG2. Dashed line indicates simple linear regression, with Pearson correlation calculated in Python.

(g) Points indicate the Pearson correlation coefficient (R^2) (blue) and slope (green) for the linear regression between gene TPM and maximum peak read density for all released ENCODE eCLIP experiments. Each point represents an individual dataset as shown in (f).

(h) Joy plot indicates (top) the distribution of unique genomic fragment values for released ENCODE eCLIP experiments, versus (bottom) the distribution of total eCLIP unique genomic fragments in the downsampled subsample where the first peak was identified in each gene, separated into bins by gene TPM.

(i) Cumulative distribution function plot indicates the number of reads needed to first detect peaks for the set of genes in indicated bins separated by gene TPM: (blue) TPM < .01, (green) .01 ≤ TPM < 1.0, (red) 1.0 ≤ TPM < 10.0, (purple) 10.0 ≤ TPM < 100.0, and (gold) 100.0 ≤ TPM.

(j) Plots indicate the cumulative fraction of genes with peaks discovered at given experimental sequencing depth, for the indicated cutoffs for peak enrichment in IP versus input: (blue) p-value ≤ .001 and fold-enrichment ≥ 8, (green) p-value ≤ .01 and fold-enrichment ≥ 4, and (red) p-value ≤ .05 and fold-enrichment ≥ 0. Peak significance was determined from two-sided Fisher's Exact Test (or Yates' Chi-Square test where appropriate) with no hypothesis testing correction, as described in Methods.

(k) Points indicate saturation rate for peak or total information content between the 90% subsampled fraction retained and 100% (full dataset) for all 223 high quality ENCODE eCLIP experiments. Grey dashed line is 5% saturation cutoff.

(l) (right) Lines show percent of additional information recovered when adding 10% additional reads for (red) HNRNPC, (blue) RBFOX2, and (green) QKI in HepG2, with number of unique (non-PCR duplicate) fragments indicated by the x-axis. Dotted line indicates the 'saturation' point at which less that 5% additional information is gained. (left) Cumulative fraction plot indicates the distribution of unique fragments when each eCLIP dataset reaches saturation. Colored points indicate depth of sequencing when HNRNPC, RBFOX2 and QKI saturate.

(m) As in (l), but points are now plotted relative to unique genomic-mapped non-PCR duplicate fragments only.

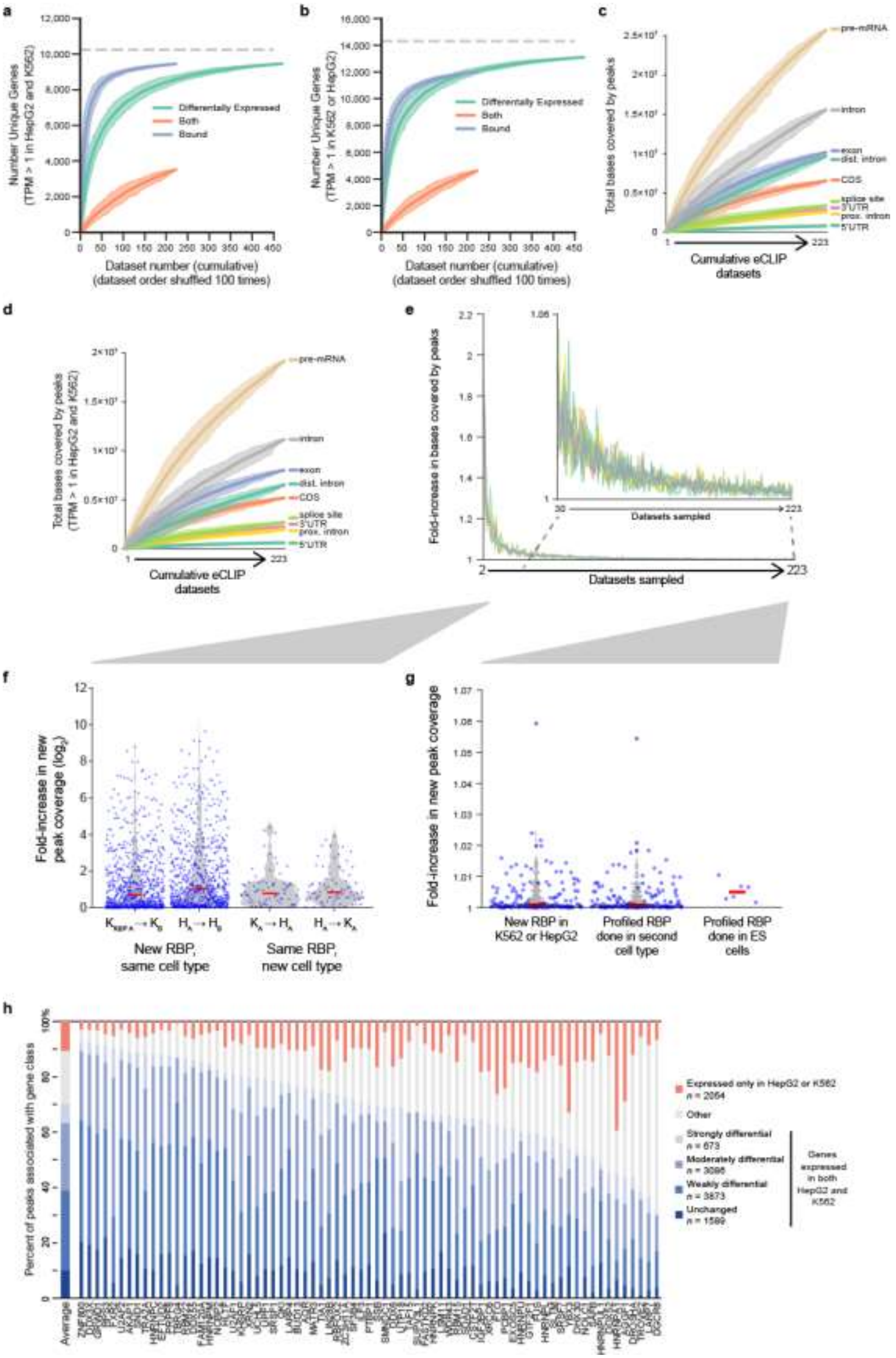
a
b
c
d
e
f
g
h
Number Unique Genes (TPM > 1 in HepG2 and K562)
Number Unique Genes (TPM > 1 in K562 or HepG2)
Differentially Expressed
Both
Bound
Dataset number (cumulative) (dataset order shuffled 100 times)
Total bases covered by peaks
Total bases covered by peaks (TPM > 1 in HepG2 and K562)
pre-mRNA
intron
exon
dist. intron
CDS
splice site
3'UTR
prox. intron
5'UTR
Cumulative eCLIP datasets
Fold-increase in bases covered by peaks
Datasets sampled
Fold-increase in new peak coverage (log2)
New RBP, same cell type
Same RBP, new cell type
Fold-increase in new peak coverage
New RBP in K562 or HepG2
Profiled RBP done in second cell type
Profiled RBP done in ES cells
Percent of peaks associated with gene class
Average
Expressed only in HepG2 or K562
n = 2064
Other
Strongly differential
n = 673
Moderately differential
n = 3096
Weakly differential
n = 3873
Unchanged
n = 1589
Genes expressed in both HepG2 and K562

Supplementary Figure 12 | Saturation of RBP binding and regulation in the transcriptome

(a-b) Lines indicate the mean of 100 random orderings of each data type for the number of genes that are (green) differentially expressed from all 472 knockdown-RNA-seq datasets (requiring FDR < 0.05 and *p*-value < 0.05, from DEseq analysis as described in Methods), (blue) bound in 223 eCLIP datasets (overlapped by a IDR-reproducible peak with $p \leq 10^{-3}$ and fold-enrichment ≥ 8 in IP versus input as described in Methods), or (orange) both bound and differentially expressed (considering 203 pairings of eCLIP and knockdown/RNA-seq for an RBP in the same cell type). The set of genes considered was (a) 10,277 genes with TPM > 1 in both HepG2 and K562, or (b) 14,273 genes with TPM > 1 in either K562 or HepG2 (indicated in grey dotted line). Shaded regions indicate tenth to ninetieth percentile.

(c-d) Lines indicate the mean cumulative fraction of bases covered by peaks for 100 random orderings of the 223 eCLIP datasets, separated by transcript regions as indicated, with shaded region indicating tenth and ninetieth percentiles. The set of genes considered was (c) all (58,540) genes in GENCODE v19, or (d) 10,277 genes with TPM > 1 in both K562 and HepG2.

(e) Data and colors as in (d), represented as fold-increase in mean bases covered by peaks from *n* to *n+1* eCLIP datasets.

(f) Points indicate the fold-increase in bases covered by peaks between sampling one or two datasets, separated by whether the second is the same RBP in a new cell type (K_A -> H_A or H_A -> K_A for RBP A profiled in K562 and then HepG2 or HepG2 and then K562 respectively) or a different RBP in the same cell type (K_A -> K_B or H_A -> H_B for RBP A followed by RBP B in either K562 or HepG2 respectively), with kernel smoothed density indicated by the shaded area. Red line indicates median.

(g) Points indicate the fold-increase in bases covered by peaks between sampling all versus leaving one dataset out, separated by whether the RBP is (left) a newly profiled RBP or (center) a previously profiled RBP profiled in a second cell type (of either K562 or HepG2). (right) The fold-increase observed if an independent eCLIP experiment performed in H1 or H9 human embryonic stem cells is added (including RBFOX2, IGF2BP3, and two replicates each for IGF2BP1 and IGF2BP2). Red line indicates median.

(h) Bars indicate the fraction of peaks observed for each RBP within sets of genes separated by their relative expression change between K562 and HepG2: unchanged (fold-difference ≤ 1.2), weakly (1.2 < fold-difference ≤ 2), moderately (2 < fold-difference ≤ 5) or strongly (fold-difference > 5) differential, or cell-type specific genes (TPM < 0.1 in one cell type and TPM ≥ 1 in the other). *n* indicates the number of genes meeting each criteria. For each RBP, the results shown are for the cell type with fewer total peaks.

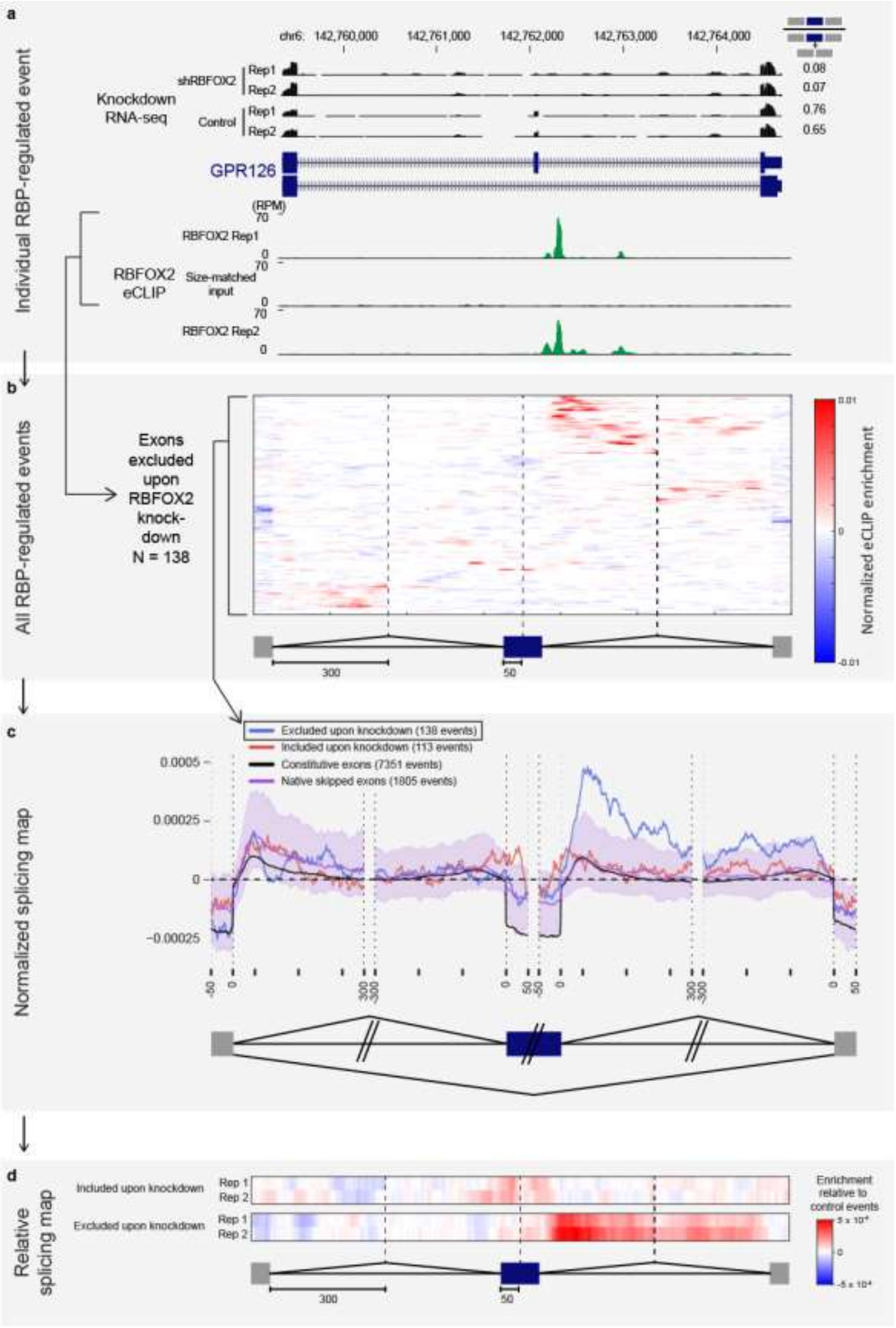
a
Individual RBP-regulated event
chr6: 142,760,000 142,761,000 142,762,000 142,763,000 142,764,000
Knockdown RNA-seq
shRBFOX2 Rep1 Rep2
Control Rep1 Rep2
0.08
0.07
0.76
0.65
GPR126
(RPM) 70
RBFOX2 eCLIP
RBFOX2 Rep1
Size-matched input
RBFOX2 Rep2
b
All RBP-regulated events
Exons excluded upon RBFOX2 knock-down N = 138
300
50
Normalized eCLIP enrichment
0.01
0
-0.01
c
Normalized splicing map
Excluded upon knockdown (138 events)
Included upon knockdown (113 events)
Constitutive exons (7351 events)
Native skipped exons (1805 events)
0.0005
0.00025
0
−0.00025
-50 0 300 -300 0 50 -50 0 300 -300 0 50
d
Relative splicing map
Included upon knockdown
Excluded upon knockdown
Rep 1
Rep 2
Enrichment relative to control events
300
50

Supplementary Figure 13 | Generation of splicing maps for RBFOX2.

(a) First, individual RBP-regulated splicing events are identified from significant changes in knockdown RNA-seq. Genome browser tracks indicate RNA-seq read density (as reads per million (RPM)) and eCLIP read density (RPM) of RBFOX2 in the same cell type, as well as its paired size-matched input. Shown are the two replicates of eCLIP performed for RBFOX2 in HepG2 cells.

(b) Next, each exon is normalized between IP versus input to obtain ‘Normalized eCLIP enrichment’. The heatmap indicates normalized eCLIP enrichment for all exons significantly excluded upon RBFOX2 knockdown.

(c) Next, a ‘splicing map’ is created by calculating the mean and standard error of the mean of normalized eCLIP enrichment for each position across the region, removing the top and bottom 5% outlier values at each position. Lines in splicing map indicate ‘Average eCLIP enrichment’, defined as the mean normalized eCLIP enrichment for exons (red) included or (blue) excluded upon RBFOX2 knockdown. Also plotted are (purple) a control set of skipped / cassette exons (referred to as ‘native' skipped exons) in wild-type HepG2 cells and (black) constitutive exons. Shaded area indicates 0.5^{th} to 99.5^{th} confidence interval obtained by 1000 random samplings of the native cassette exon control set (performed independently using the number of events in either excluded or included sets, and plotting the larger of the two confidence intervals).

(d) A final simplified splicing map vector was calculated by subtracting the normalized eCLIP enrichment of control native cassette exons from that of either included or excluded exons at each position to calculate ‘Enrichment relative to control events’.

Supplementary References

1 Gerstberger, S., Hafner, M. & Tuschl, T. A census of human RNA-binding proteins. *Nature reviews. Genetics* **15**, 829-845, doi:10.1038/nrg3813 (2014).

2 Consortium, G. T. Human genomics. The Genotype-Tissue Expression (GTEx) pilot analysis: multitissue gene regulation in humans. *Science* **348**, 648-660, doi:10.1126/science.1262110 (2015).

3 Bray, N. L., Pimentel, H., Melsted, P. & Pachter, L. Near-optimal probabilistic RNA-seq quantification. *Nature biotechnology* **34**, 525-527, doi:10.1038/nbt.3519 (2016).

4 Zhang, X. *et al.* An Atomic Structure of the Human Spliceosome. *Cell* **169**, 918-929 e914, doi:10.1016/j.cell.2017.04.033 (2017).

5 Johnson, W. E., Li, C. & Rabinovic, A. Adjusting batch effects in microarray expression data using empirical Bayes methods. *Biostatistics* **8**, 118-127, doi:10.1093/biostatistics/kxj037 (2007).

6 Van Nostrand, E. L. *et al.* Robust transcriptome-wide discovery of RNA-binding protein binding sites with enhanced CLIP (eCLIP). *Nature methods* **13**, 508-514, doi:10.1038/nmeth.3810 (2016).

7 Van Nostrand, E. L. *et al.* CRISPR/Cas9-mediated integration enables TAG-eCLIP of endogenously tagged RNA binding proteins. *Methods* **118-119**, 50-59, doi:10.1016/j.ymeth.2016.12.007 (2017).

8 Dobin, A. *et al.* STAR: ultrafast universal RNA-seq aligner. *Bioinformatics* **29**, 15-21, doi:10.1093/bioinformatics/bts635 (2013).

9 Bao, W., Kojima, K. K. & Kohany, O. Repbase Update, a database of repetitive elements in eukaryotic genomes. *Mobile DNA* **6**, 11, doi:10.1186/s13100-015-0041-9 (2015).

10 Lovci, M. T. *et al.* Rbfox proteins regulate alternative mRNA splicing through evolutionarily conserved RNA bridges. *Nature structural & molecular biology* **20**, 1434-1442, doi:10.1038/nsmb.2699 (2013).

11 Yee, B. A., Pratt, G. A., Graveley, B. R., Van Nostrand, E. L. & Yeo, G. W. RBP-Maps enables robust generation of splicing regulatory maps. *Rna* **25**, 193-204, doi:10.1261/rna.069237.118 (2019).

12 Li, Q. H., Brown, J. B., Huang, H. Y. & Bickel, P. J. Measuring Reproducibility of High-Throughput Experiments. *Ann Appl Stat* **5**, 1752-1779, doi:10.1214/11-AOAS466 (2011).

13 Hodges, P. E. & Beggs, J. D. RNA splicing. U2 fulfils a commitment. *Current biology : CB* **4**, 264-267 (1994).

14 Townley-Tilson, W. H., Pendergrass, S. A., Marzluff, W. F. & Whitfield, M. L. Genome-wide analysis of mRNAs bound to the histone stem-loop binding protein. *Rna* **12**, 1853-1867, doi:10.1261/rna.76006 (2006).

15 Borowski, L. S., Dziembowski, A., Hejnowicz, M. S., Stepien, P. P. & Szczesny, R. J. Human mitochondrial RNA decay mediated by PNPase-hSuv3 complex takes place in distinct foci. *Nucleic acids research* **41**, 1223-1240, doi:10.1093/nar/gks1130 (2013).

16 Landt, S. G. *et al.* ChIP-seq guidelines and practices of the ENCODE and modENCODE consortia. *Genome research* **22**, 1813-1831, doi:10.1101/gr.136184.111 (2012).

17 Silverman, I. M. *et al.* RNase-mediated protein footprint sequencing reveals protein-binding sites throughout the human transcriptome. *Genome biology* **15**, R3, doi:10.1186/gb-2014-15-1-r3 (2014).

18 Ishimura, R. *et al.* RNA function. Ribosome stalling induced by mutation of a CNS-specific tRNA causes neurodegeneration. *Science* **345**, 455-459, doi:10.1126/science.1249749 (2014).
