# Supplementary material for: A large-scale binding and functional map of human RNA-binding proteins
Source: Nature. 2020 Jul 29;583(7818):711–9. doi: 10.1038/s41586-020-2077-3 (PMC7410833; doi:10.1038/s41586-020-2077-3)
Supplement: Supplementary file 2 — Reporting Summary [file 41586_2020_2077_MOESM2_ESM.pdf]

nature research

Corresponding author(s): Graveley BR

Last updated by author(s): Apr 16, 2019

Reporting Summary

Nature Research wishes to improve the reproducibility of the work that we publish. This form provides structure for consistency and transparency in reporting. For further information on Nature Research policies, see Authors & Referees and the Editorial Policy Checklist.

Statistics

For all statistical analyses, confirm that the following items are present in the figure legend, table legend, main text, or Methods section.

n/a	Confirmed	
☐	☒	The exact sample size (*n*) for each experimental group/condition, given as a discrete number and unit of measurement
☐	☒	A statement on whether measurements were taken from distinct samples or whether the same sample was measured repeatedly
☐	☒	The statistical test(s) used AND whether they are one- or two-sided<br>*Only common tests should be described solely by name; describe more complex techniques in the Methods section.*
☐	☒	A description of all covariates tested
☐	☒	A description of any assumptions or corrections, such as tests of normality and adjustment for multiple comparisons
☐	☒	A full description of the statistical parameters including central tendency (e.g. means) or other basic estimates (e.g. regression coefficient) AND variation (e.g. standard deviation) or associated estimates of uncertainty (e.g. confidence intervals)
☐	☒	For null hypothesis testing, the test statistic (e.g. *F*, *t*, *r*) with confidence intervals, effect sizes, degrees of freedom and *P* value noted<br>*Give P values as exact values whenever suitable.*
☒	☐	For Bayesian analysis, information on the choice of priors and Markov chain Monte Carlo settings
☒	☐	For hierarchical and complex designs, identification of the appropriate level for tests and full reporting of outcomes
☐	☒	Estimates of effect sizes (e.g. Cohen's *d*, Pearson's *r*), indicating how they were calculated

Our web collection on statistics for biologists contains articles on many of the points above.

Software and code

Policy information about availability of computer code

Data collection	All data was collected from files available at the ENCODE DCC https://www.encodeproject.org (accession identifiers in Supplementary Data 2)
Data analysis	All pipelines are described in documentation at the ENCODE DCC (https://www.encodeproject.org); Custom eCLIP data processing scripts are available at https://github.com/gpratt/gatk/releases/tag/2.3.2; RBNS analysis code is available at https://bitbucket.org/pfreese/rbns_pipeline/ .

For manuscripts utilizing custom algorithms or software that are central to the research but not yet described in published literature, software must be made available to editors/reviewers. We strongly encourage code deposition in a community repository (e.g. GitHub). See the Nature Research guidelines for submitting code & software for further information.

Data

Policy information about availability of data

All manuscripts must include a data availability statement. This statement should provide the following information, where applicable:

- Accession codes, unique identifiers, or web links for publicly available datasets
- A list of figures that have associated raw data
- A description of any restrictions on data availability

All data is publicly available at the ENCODE DCC (https://www.encodeproject.org), with accession identifiers listed in Supplementary Data 2

Field-specific reporting

Please select the one below that is the best fit for your research. If you are not sure, read the appropriate sections before making your selection.

☒ Life sciences ☐ Behavioural & social sciences ☐ Ecological, evolutionary & environmental sciences

For a reference copy of the document with all sections, see nature.com/documents/nr-reporting-summary-flat.pdf

Life sciences study design

All studies must disclose on these points even when the disclosure is negative.

Sample size

eCLIP, ChIP-seq, and knockdown/RNA-seq experiments were performed in biological duplicate (defined as cell lines grown separately according to ENCODE guidelines). RBNS experiments were performed at multiple (typically 5) concentrations.

Data exclusions

Analyses were performed using all datasets deposited at the ENCODE DCC. A set of eCLIP datasets not meeting quality metrics were included in extended data figures describing the development of metrics (and released at the Gene Expression Omnibus), but were not included in other analyses. For ChIP-seq figures, 5 datasets that showed reproducible signal but less than 200 reproducible peaks in the 'optimal' set were released at the ENCODE DCC but not included in further analysis.

Replication

eCLIP, ChIP-seq, and knockdown/RNA-seq experiments were performed in biological duplicate (defined as cell lines grown separately according to ENCODE guidelines). Reproducibility of eCLIP and ChIP-seq peaks across biological replicates was assessed using the Irreproducible Discovery Rate (IDR) methodology (see Methods). Significant changes in gene expression or splicing in knockdown/RNA-seq datasets was assayed across biological replicates using DeSeq2 or rMATS respectively. RBNS experiments were performed at multiple (typically 5) concentrations, with motifs required to reproduce across concentrations.

Randomization

Paired input controls were performed for eCLIP and ChIP-seq and served as normalization controls. For knockdown/RNA-seq, a scrambled control was performed within each experimental batch, which served as a within-batch control to identify significant gene expression and splicing changes. Batch correction was additionally performed to aid in global analyses (Supplementary Fig. 6 and associated methods).

Blinding

Experimenters were generally unaware of expected results for individual proteins, but were aware of antibody and/or shRNA reagent labels.

Reporting for specific materials, systems and methods

We require information from authors about some types of materials, experimental systems and methods used in many studies. Here, indicate whether each material, system or method listed is relevant to your study. If you are not sure if a list item applies to your research, read the appropriate section before selecting a response.

Materials & experimental systems

n/a	Involved in the study	
☐	☒	Antibodies
☐	☒	Eukaryotic cell lines
☒	☐	Palaeontology
☒	☐	Animals and other organisms
☒	☐	Human research participants
☒	☐	Clinical data

Methods

n/a	Involved in the study	
☐	☒	ChIP-seq
☒	☐	Flow cytometry
☒	☐	MRI-based neuroimaging

Antibodies

Antibodies used

Information for each antibody used is linked to each dataset at the ENCODE DCC (https://encodeproject.org). Antibodies used for eCLIP are listed in Supplemental Data 4.

Validation

Each antibody for eCLIP and ChIP-seq was required to meet both primary (IP-western) and secondary (knockdown-western or IP-mass spectrometry) validation. Validation data is available for each antibody at the ENCODE DCC (https://encodeproject.org).

Eukaryotic cell lines

Policy information about cell lines

Cell line source(s)

Cell lines were purchased from ATCC.

Authentication

Cell lines were not formally authenticated, but confirmation of expected gene expression patterns were performed for RNA-seq and eCLIP experiments.

Mycoplasma contamination	Cell lines were routinely (every ~2 months) tested for mycoplasma using MycoAlert (Lonza).
Commonly misidentified lines (See ICLAC register)	None used

ChIP-seq

Data deposition

☒ Confirm that both raw and final processed data have been deposited in a public database such as GEO.

☒ Confirm that you have deposited or provided access to graph files (e.g. BED files) for the called peaks.

Data access links *May remain private before publication.*	All data is available at the ENCODE DCC (https://encodeproject.org). Individual dataset accession identifiers are listed in Supplementary Data 2. All eCLIP (ENCSR456FVU) and ChIP-seq (ENCSR999WIC) datasets are available at the above accession IDs.
Files in database submission	Individual dataset accession identifiers are listed in Supplemental Data 2.
Genome browser session (e.g. UCSC)	UCSC genome browser tracks can be generated via links on the ENCODE DCC for each dataset

Methodology

Replicates	2 biological replicates were performed for each experiment
Sequencing depth	Sequencing depth information is provided in Supplementary Data 4 (eCLIP) and Supplementary Data 7 (ChIP-seq)
Antibodies	Each antibody for eCLIP and ChIP-seq was required to meet both primary (IP-western) and secondary (knockdown-western or IP-mass spectrometry) validation. Catalog and validation data is available for each antibody is linked to each dataset at the ENCODE DCC (https://encodeproject.org).
Peak calling parameters	Primary data analysis of eCLIP data (including adapter trimming, read mapping, cluster identification, and input normalization) was performed as previously published6 and is provided (including description of steps as well as commands run) as a 'Pipeline Protocol' attached to each eCLIP dataset available on the ENCODE website at https://www.encodeproject.org/documents/3b1b2762-269a-4978-902e-0e1f91615782/@@download/attachment/eCLIP_analysisSOP_v2.0.pdf) (Supplementary Fig. 9a). Briefly, sequencing reads are first demultiplexed using dual indices with standard tools provided by Illumina. Next, reads were further demultiplexed based on in-line barcodes (present in read 1) (Supplementary Data 13). At this step, a unique molecular identifier (either N5 or N10) was removed from the beginning of read 2 and saved for use at the later PCR duplicate removal step. Next, potential adapter sequences were removed using cutadapt (v1.8.1), performed in two steps to properly remove non-full length adapter sequences we observed to drive artifact peak identification. At this step, reads with less than 18 bases were removed from further analysis. Next, we mapped reads using STAR (v2.4.0i) against a database of repetitive elements (derived from RepBase (v18.05) with the addition of elements including the 45S ribosomal RNA precursor), and removed reads with identified mapping (an independent method was derived to quantify mapping to repetitive elements, described in the next section). Reads were then mapped against the human genome using STAR (v2.4.0i), requiring unique mapping (all analyses described in this manuscript used mapping to GRCh37 and GENCODE v19 annotations, but mapping to GRCh38 and GENCODE v24 annotations were also deposited at the ENCODE portal). PCR duplicate reads were then identified as those with the same mapped start position and unique molecular identifier and were removed using custom scripts to obtain unique fragments. Read clusters were identified using CLIPper, which applies spine-fitting to identify clusters of enriched read density above local, transcript (both pre-mRNA and mRNA), and whole-genome background. Whole reads were used for this broad analysis of RBPs as the use of read 3' ends only can cause decreased signal-to-noise for the subset of RBPs that do not crosslink in close proximity to their RNA motif. However, we note that future re-analyses restricting to only read 3' ends likely will provide increased resolution for motif and splicing regulatory map analyses for some RBPs. Finally, clusters identified in IP samples were compared against paired size-matched input to obtain significantly enriched peaks using a Fisher's Exact test (or Yates' Chi-Square test if all observed and expected values were above 5), with p-values reported not corrected for multiple hypothesis testing. An average of 6.9% of clusters were significantly enriched, although this was highly variable across the 223 datasets (Extended Data Fig. 1f). The number of significantly enriched peaks was highly correlated between replicates, indicating the capture of RBP-specific biological signal (Supplementary Fig. 9b) (Supplementary Data 4). To identify reproducible and significantly enriched peaks across biological replicates, we used a modified Irreproducible Discovery Rate (IDR) method (Supplementary Fig. 9c).<br><br>RBP ChIP-seq datasets used in this study were processed by ENCODE Data Coordinating Center with the same uniform processing pipelines previously described for transcription factor ChIP-seq (https://www.encodeproject.org/chip-seq/transcription_factor/). After removing low quality and PCR duplicate reads, peaks were identified with SPP and reproducible peaks across biological replicates were identified with the IDR pipeline to yield 2 sets (optimal and conservative) of peaks at IDR threshold=0.05.
Data quality	For eCLIP, we first require successful immunoprecipitation as assayed by IP-western. Next, we require a minimum of 1 million usable (uniquely mapped, non-PCR duplicate) reads and the presence of significant reproducible peak signal (identified using a pipeline we developed applying the Irreproducibility Discovery Rate (IDR) framework previously applied by the ENCODE group for identifying reproducible ChIP-seq peaks). Additionally, we developed an approach to quantify mapping to retrotransposable elements and other multicopy RNAs with many pseudogenes (including rRNA, Y-RNAs, and others), which enabled us to assess quality of RBPs that bind to these elements rather than show peaks from unique genomic mapped reads. In both cases, we allow rare exceptions that show clear biologically relevant signal below these

thresholds.
For ChIP, antibodies were first validated using standard ENCODE procedures, requiring primary (IP-western) and secondary (knockdown-Western) validation. Next, data were processed using the previously described reference ENCODE ChIP-seq pipeline, and assayed by typical quality metrics including read depth and reproducibility by IDR analysis. As previously described for ChIP-seq analysis of transcription factors, we required 'pass' or 'borderline' by standard IDR reproducibility metrics. Next, we generally required >10 million usable reads from each replicate, although a limited set of datasets were exempted if manual inspection indicated significant reproducible signal at lower read depths. Finally, for all analyses only the set of reproducible peaks identified after IDR analysis were used, and only datasets with >200 peaks were included. The quality control metrics of the datasets are summarized in Supplementary Data 7.

Software

ChIP-seq analysis was performed using the publicly described ENCODE pipeline (linked to each dataset on the ENCODE portal and fully described here https://www.encodeproject.org/documents/6f6351d4-9310-4a3b-a3c2-70ecac47b28b/@@download/attachment/ChIP-seq_Mapping_Pipeline_Overview.pdf).
